# Genetic drivers of etiologic heterogeneity in thyroid cancer

**DOI:** 10.1038/s41467-026-75111-8

**Published:** 2026-07-10

**Authors:** Yon Ho Jee, Nikita Pozdeyev, Christopher R. Gignoux, Samantha White, Bryan R. Haugen, Anurag Verma, Raitis Peculis, Vita Rovite, Ashley J. Mulford, Alan R. Sanders, Cari M. Kitahara, Mary Pat Reeve, Peter Kraft, Alicia R. Martin

**Affiliations:** 1 Analytic and Translational Genetics Unit, Massachusetts General Hospital, Boston, MA USA; 2 Stanley Center for Psychiatric Research, Broad Institute of MIT and Harvard, Cambridge, MA USA; 3 Program in Medical and Population Genetics, Broad Institute of MIT and Harvard, Cambridge, MA USA; 4 Department of Biomedical Informatics, University of Colorado Anschutz Medical Campus, Aurora, CO USA; 5 Colorado Center for Personalized Medicine, University of Colorado Anschutz Medical Campus, Aurora, CO USA; 6 Division of Endocrinology, Diabetes and Metabolism, Department of Medicine, University of Colorado Cancer Center, University of Colorado Anschutz Medical Campus, Aurora, CO USA; 7 Human Medical Genetics and Genomics Program, University of Colorado Anschutz Medical Campus, Aurora, CO USA; 8 Division of Translational Medicine and Human Genetics, Department of Medicine, University of Pennsylvania, Philadelphia, PA USA; 9 Latvian Biomedical Research and Study Centre, Riga, Latvia; 10 Genomic Health Initiative, Endeavor Health, Evanston, IL USA; 11 Department of Psychiatry and Behavioral Neuroscience, University of Chicago, Chicago, IL USA; 12 Division of Cancer Epidemiology and Genetics, Radiation Epidemiology Branch, National Cancer Institute, National Institutes of Health, Rockville, MD USA; 13 Institute for Molecular Medicine Finland, University of Helsinki, Helsinki, Finland; 14 Division of Cancer Epidemiology and Genetics,Transdivisional Research Program, National Cancer Institute, National Institutes of Health, Rockville, MD USA

**Keywords:** Cancer, Functional genomics

## Abstract

Thyroid cancer is the most common endocrine malignancy, yet its biological underpinnings remain incompletely understood. Here we show that common risk alleles for thyroid cancer point to distinct biological pathways underlying disease susceptibility. We perform a multi-ancestry genome-wide association meta-analysis of thyroid cancer (16,167 cases and 2,430,374 controls), identifying 51 independent loci, including 21 not previously reported. By integrating these loci with genetic associations for 151 thyroid-cancer-related traits, we identify pleiotropic mechanistic clusters linked to thyroid function, oncogenic pathways, and mixed physiological function. Two thyroid-specific clusters, associated with thyroid stimulating hormone or thyroid growth and function, are enriched in thyroid tissues. Oncogenic clusters include DNA repair (*ATM*, *CHEK2*, *TP53*) and telomere maintenance (*TERT*) genes, implicating shared cancer mechanisms. Cluster-specific polygenic scores are associated with thyroid disease, cancer, and metabolic traits across ancestry groups, suggesting distinct genetic subtypes of thyroid cancer risk and supporting pleiotropy-based approaches to genetic risk stratification.

## Abstract (web summary)

Thyroid cancer risk reflects multiple biological pathways that remain incompletely de-fined. Here, the authors analyze genetic data from over 16,000 thyroid cancer patients and over 2.4 million controls to identify risk variants and group them into clusters linked to thyroid hormone regulation, DNA damage response, and telomere maintenance.

## Introduction

Thyroid cancer is the most prevalent endocrine malignancy, with an incidence rate of 22.8 per 100,000 per year in women and 8.0 per 100,000 per year in men in the U.S. between 2014 and 2018^1^. The vast majority of thyroid cancers are papillary thyroid carcinomas (PTC) (~85%), followed by follicular thyroid carcinomas (FTC) (~10%), while medullary (MTC) (~2–3%) and anaplastic (ATC) thyroid carcinomas (~1–2%) are considerably rarer^2^. Prognosis varies substantially by histologic subtype: PTC and FTC, which together comprise the differentiated thyroid cancers arising from thyroid follicular epithelial cells, are associated with favorable outcomes, with high 10-year survival rate (90 to 95%)^3^; in contrast, ATC is highly aggressive, with a median survival of 3–5 months^4^. MTC, which originates from parafollicular C cells, is frequently associated with germline RET mutations in hereditary syndromes such as multiple endocrine neoplasia type 2^5,6^. Thyroid cancer demonstrates moderate heritability, with estimates suggesting genetic factors account for approximately 53% of the disease risk^7^. Globally, thyroid cancer incidence rates have markedly increased in recent decades^8^. While this rise has been largely attributed to improved detection of small, localized slow-growing tumors with high survival rates, the concurrent increase in advanced-stage tumors and thyroid cancer mortality suggests that additional etiological factors contribute to disease progression^9,10^. Established risk factors include childhood exposure to ionizing radiation, while emerging evidence highlights the role of obesity and endocrine-disrupting chemicals in thyroid carcinogenesis^11–13^. However, the underlying biological mechanisms linking these risk factors to thyroid cancer remain poorly understood.

Thyroid function plays a critical role in metabolic homeostasis, and its dysregulation has been linked to benign thyroid disorders, such as goiter and hyperthyroidism, both of which have been associated with an increased risk of thyroid cancer^14^. Additionally, autoimmune thyroid diseases, including Hashimoto’s thyroiditis and Graves’ disease, have been linked to an elevated risk of thyroid cancer, suggesting a potential immune-mediated component in thyroid tumorigenesis^15,16^. Thyroid-stimulating hormone (TSH), a key regulator of thyroid follicular cell growth, has been implicated in thyroid cancer development^17,18^. Genetic variants associated with TSH levels may exert pleiotropic effects on thyroid function through the negative feedback regulation of thyroid hormones, as well as on thyroid cell proliferation, potentially increasing the risk of both goiter and thyroid cancer^19^. However, studies have reported an inverse causal association between TSH levels and thyroid cancer risk, suggesting a more complex interplay between thyroid function and malignancy that warrants further investigation^19–21^. Beyond thyroid hormones, other endocrine factors, including sex hormones, growth hormones, and stress hormones, have been implicated in thyroid tumorigenesis^22^, although the mechanisms underlying the crosstalk between these hormonal systems and their influence on thyroid cancer progression remain unclear^23^.

As genome-wide association studies (GWAS) expand across multiple traits, new opportunities have emerged to leverage cross-trait genetic association patterns to uncover shared disease mechanisms. Studies have demonstrated widespread pleiotropy across the human genome, revealing that genetic variants often influence multiple diseases through shared molecular mechanisms^24^. Pleiotropy dissection provides a powerful framework to cluster genetic associations into biologically meaningful groups, enabling a deeper understanding of how genetic variation drives disease heterogeneity^25^. Recent applications of this approach in type 2 and gestational diabetes^26–29^, autoimmune hypothyroidism^30^, and lung diseases^31,32^ have demonstrated its utility in identifying genetic subgroups that would otherwise be obscured in single-trait GWAS analyses. However, pleiotropy dissection has not yet been applied to thyroid cancer, limiting our ability to differentiate mechanistically distinct genetic pathways underlying its pathophysiology. Given the substantial overlap in genetic susceptibility loci across thyroid diseases^18^, large-scale pleiotropy-informed analyses of thyroid cancer could provide an opportunity to elucidate the complex underlying biological mechanisms of thyroid dysfunction and oncogenesis.

Here, we present findings from a meta-analysis of thyroid cancer GWAS data comprising over 2.5 million individuals of diverse ancestry, more than doubling the effective sample size of prior efforts^17,33–44^. Using cross-trait associations, we characterize the etiological heterogeneity of thyroid cancer and identify genetic clusters that are enriched for distinct tissue- and cell-specific regulatory elements. Furthermore, we construct partitioned polygenic scores (pPGSs) across multiple ancestry groups and assess their associations with thyroid cancer-related outcomes, advancing our understanding of the genetic architecture underlying thyroid cancer.

## Results

### Discovery of thyroid cancer loci

**Fig. 1:**
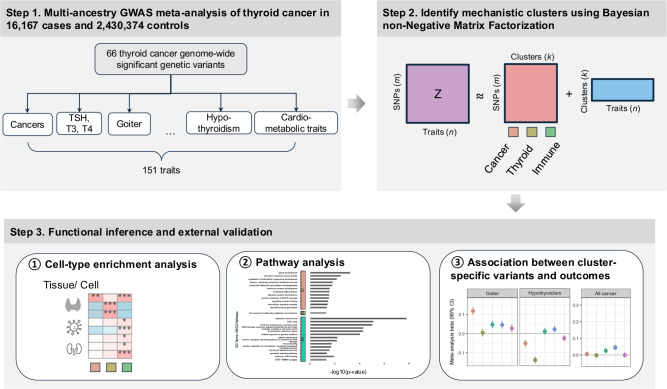
Overview of study design integrating multi-ancestry GWAS, pleiotropy-informed clustering, and functional validation. Schematic of the analytic framework used to identify and interpret germline risk mechanisms in thyroid cancer. Step 1: A multi-ancestry genome-wide association meta-analysis of 16,167 thyroid cancer cases and 2,430,374 controls identified 66 independent genome-wide significant variants. Pleiotropic associations with 151 traits were aggregated across multiple biobanks. Step 2: Bayesian non-negative matrix factorization (bNMF) was applied to the variant–phenotype association matrix to derive mechanistic clusters. Step 3: Functional interpretation of each cluster included (1) cell-type specific enrichment, (2) pathway enrichment analysis, and (3) external replication of cluster-specific genetic risk in the All of Us cohort across disease domains.

We conducted a fixed-effect GWAS meta-analysis of thyroid cancer using inverse-variance weighted data from 18 biobanks across diverse ancestry groups, comprising 16,167 cases and 2,430,374 controls (Fig. 1, Supplementary Data 2, Table 1). After performing QC, we identified 66 independent thyroid cancer association signals (*P* < 5 × 10^−8^), each represented by an independent index variant (Methods). These 66 association signals mapped to 51 loci, of which 21 (41%) loci have not been previously reported in thyroid cancer GWASs (Supplementary Data 2, Table 2).

**Table 1:** Overview of thyroid cancer genetic clusters

Cluster (no. of variants)	Key top-weighted traits^a^	Key top-weighted loci
1. Thyroid Gland Dysfunction (12)	TSH(-), Nontoxic multinodular goitre(+), FT3( + ), FT4( + ), QRS duration(-), Urate(-)	*MAF, CAPZB, VAV3, INSR, MBIP, NRG1, TG, RPSAP52, FOXE1, LINC00609, TGFB2, DIRC3*
2. Thyroid Overactivity (2)	Disorders of the thyroid gland(-), TSH(-), FT4(-), TT3(+), Nontoxic multinodular goitre(-), FT3(+)	*FOXE1, VAV3*
3. DNA Damage Response (9)	Leiomyoma of uterus(+), All cancers(+), SHBG(-), Prostate cancer(+), BMR(+), Skin cancer(+), RBC( + ), MRV(-), Height(+), Polycystic ovarian syndrome(+), PP(-), Platelet crit(+), FT4(+), Breast cancer(+)	*TP53, ATM, CHEK2, TTC28, STN1, TERT, EIF4ENIF1, SORCS3*
4. Telomere Maintenance (10)	Leukocyte telomere length(+), Leiomyoma of uterus(+), MRV(-), Platelet crit(+), RBC(+), Eosinophil percentage(-), Neutrophil percentage(+), Total protein(-), Monocyte percentage(-),	*ACTRT3/TERC, STN1, TERT, TYMS, GPR37, ZNF257, SLK, UCKL1, ENOSF1, LOC100287846*
5. Mixed function (3)	Heel BMD( + ), QRS duration(+), ALP(+), Platelet distribution width(+), P duration(+), RBC distribution width(+), TG(-), Neuroticism score(-), eGFR(+), AST(+), Eosinophil percentage(+), ALT( + ), GGT( + ), MAP(+), Total protein(+)	*PRAG1, CEP120, SMAD3*

^a^+/−: thyroid cancer risk alleles associated with increased or decreased phenotype values.

*ALP* Alkaline phosphatase, *ALT* Alanine aminotransferase, *AST* Aspartate aminotransferase, *BMR* basal metabolic rate, *BP* blood pressure, *BMD* bone mineral density, *eGFR* Estimated glomerular filtration rate, *FT3* free T3, *GGT* Gamma glutamyltransferase, *MAP* Mean arterial pressure, *MRV* Mean reticulocyte volume, *PP* Pulse pressure, *RBC* Red blood cell, *SHBG* sex hormone binding globulin, *TG* Triglycerides, *TSH* thyroid stimulating hormone, *TT3* total T3.

Our top thyroid cancer loci mapped to genes with known functional relevance in thyroid tumorigenesis. The most significant association was observed near *PTCSC2/FOXE1* (rs4273946; beta-C = −0.438, *p* = 6.07 × 10^−235^), a gene previously implicated in thyroid cancer susceptibility^17,45^ (Supplementary Fig. 1a). The second most significant variant was located in the intergenic region of *DIRC3* (rs57481445; beta-A = −0.290, *p* = 1.34 × 10^−117^), supporting prior evidence of its regulatory role in thyroid cancer risk^46^. Additional significant intronic variants included rs4733129 in *NRG1* (beta-T = −0.256, *p* = 3.68 × 10^−100^), rs17020141 in *VAV3* (beta-A = −0.188, *p* = 6.09 × 10^−21^), and rs12463278 in *INSR* (beta-A = −0.134, *p* = 7.23 × 10^−15^). These genes are involved in TSH regulation and hypothyroidism, further supporting their potential contribution to thyroid cancer pathogenesis^18,19,34^.

Several thyroid cancer-associated variants overlapped with well-established cancer predisposition genes, reinforcing their role in oncogenic pathways. Notably, rs78378222 at 3’ UTR of *TP53*, rs186430430 (intronic) in *CHEK2*, rs10069690 (intronic) in *TERT*, and rs72743461 (intronic) in *SMAD3* were significantly associated with both thyroid cancer and other malignancies, particularly breast, colorectal, glioma, and prostate cancers^47–51^. Furthermore, a missense variant in *ATM* (rs1800057; beta-C = −0.252, *p* = 2.89 × 10^−08^, AF = 0.0226) was identified (Supplementary Fig. 1b). ATM plays a critical role in the DNA damage response pathway^52^.

Two additional variants may implicate broader endocrine regulatory mechanisms in thyroid cancer susceptibility. For example, the association between rs2842870 (intronic) and thyroid cancer in *PMF1* (beta-T = −0.085, *p* = 1.63 × 10^−12^) was previously also associated with mosaic loss of the Y chromosome (mLOY) (rs2842873 near *PMF1*: beta-T = −0.056, *p* = 1.5 × 10^−36^ in a cohort of 95,380 Japanese men)^53^. Prior studies have demonstrated that Y-chromosome alterations are linked to increased risk of a range of malignancies that include lung, bladder, and prostate cancers^54^. While thyroid cancer tends to be more aggressive in males^55^, our meta-analysis was not stratified by sex, and further investigation is needed to determine whether sex-specific tumorigenesis mechanisms contribute to risk at this locus. Another intronic variant that is associated with thyroid cancer, rs726686 (non-coding RNA intronic) in *LINC02428* (beta-A = 0.082, *p* = 1.03 × 10^−9^, AF = 0.1812), was previously associated with sex hormone-binding globulin (SHBG) (beta-A = 0.008, *p* = 1.1 × 10^−13^)^56^. SHBG is a key regulator of circulating sex hormones and has been implicated in the etiology of multiple cancers^56,57^; however, its relationship to thyroid cancer risk remains unclear^15,58,59^. Given the pleiotropic nature of SHBG-associated variants^60^ and the lack of consistent directional effects on thyroid cancer risk, this association should be interpreted with caution. Together, these findings suggest broader involvement of endocrine regulatory axes, including thyroidal, gonadal, and adrenal pathways, in thyroid cancer susceptibility^23^.

### Heterogeneity of GWAS loci across thyroid cancer subtypes

**Fig. 2:**
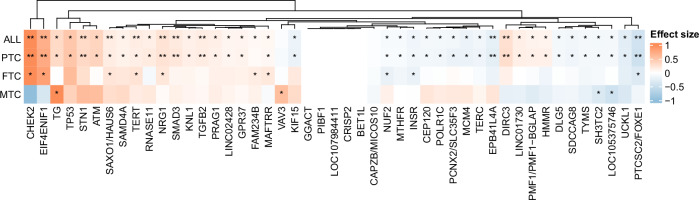
Subtype-specific effect sizes for thyroid cancer risk variants. Heatmap of beta coefficients for 51 loci across thyroid cancer subtypes in FinnGen: papillary (PTC), follicular (FTC), and medullary (MTC). Each column represents a locus, and rows correspond to subtype-specific effect sizes. Variants are clustered by similarity in effect profiles. Effect sizes represent log-odds ratios from two-sided association tests in subtype-specific GWAS analyses. *P < 0.05 (nominal significance); **P < 5 × 10⁻⁸ (genome-wide significance threshold). Source data are provided as a Source Data file.

To characterize the heterogeneity of germline risk across thyroid cancer subtypes, we evaluated 66 lead thyroid cancer variants from the meta-analysis in FinnGen across PTC, FTC, and MTC. In FinnGen, 86% (2534/2963) of thyroid cancers were papillary, 13% (385/2963) follicular, 3% (75/2963) medullary, and 1.7% (49/2963) anaplastic (Supplementary Fig. 2a). The remaining 5.8% were listed generally as thyroid cancer without a particular designation. PTC and FTC, which together comprise the differentiated thyroid cancers arising from thyroid follicular epithelial cells, demonstrated broadly concordant effects across most loci (Fig. 2, Supplementary Data 2, Table 3). Several loci showed strong and consistent effects in PTC and FTC, including *CHEK2* (rs186430430; PTC beta=1.29, *p* = 1.75 × 10^−29^; FTC beta = 1.02, *p* = 0.0020), *EIF4ENIF1* (rs34152346; PTC beta = 0.74, *p* = 9.20 × 10^−12^; FTC beta = 0.83, *p* = 0.03), and *PTCSC2/FOXE1* (rs4273946; PTC beta = −0.46, *p* = 8.36 × 10^−58^; FTC beta = −0.32, *p* = 2.04 × 10^−5^). In contrast, effect sizes in MTC were generally attenuated or absent and should be interpreted as exploratory, given both its distinct cellular origin from parafollicular C cells and the limited number of cases (*n* = 75), which constrains statistical power. Although *VAV3* (rs17020141) appeared nominally associated with MTC (beta = 0.57, *p* = 0.029), this association was not observed in the overall thyroid cancer in FinnGen (beta = −0.04, *p* = 0.34), and was directionally inconsistent with PTC (beta = −0. 077, *p* = 0.092) or FTC (beta = −0.035, *p* = 0.77), suggesting that the signal may reflect noise or cohort-specific variation rather than true MTC-specific susceptibility. Conversely, the *TG* locus (rs78775620) showed robust associations in both PTC (rs78775620 beta=0.33, *p* = 1.86 × 10^−4^) and MTC (beta = 1.23, *p* = 0.031), but not FTC (beta = −0.01, *p* = 0.96), indicating partially overlapping germline influences across histologies. However, given the limited number of MTC cases, these findings should be interpreted with caution and warrant replication in larger, subtype-specific cohorts. In FinnGen, 7.1% (165/2334) of PTC patients had concurrent diagnoses of FTC, and only 0.7% (16/2334) had overlap with MTC (Supplementary Fig. 2a). Sensitivity analyses restricted to histologically exclusive PTC and FTC cases yielded effect estimates highly consistent with the primary analyses, with the same direction and similar magnitudes across loci, indicating that the observed subtype-specific patterns were not driven by co-diagnosed cases (Supplementary Fig. 2b, Supplementary Data 2, Table 3).

### Mechanistic clusters of thyroid cancer index variants

In post-GWAS analysis, we next aimed to identify distinct genetic mechanisms driving thyroid cancer using the study design outlined in Fig. 1. Genetic correlation analysis with 151 thyroid-cancer-related traits (Methods; Supplementary Data 2, Table 4) revealed that thyroid cancer shares significant yet moderate genome-wide correlation with several thyroid disorders, immune-related, and cancer phenotypes (significant *r*_g_ range: −0.24–0.53) (Supplementary Data 2, Table 12, Supplementary Fig. 3). Notably, we observed positive genetic correlations with thyroid diseases such as Graves’ disease (*r*_g_ = 0.212, *p* = 5.43 × 10^−4^), nontoxic goiter (*r*_g_ = 0.427, *p* = 0.007), autoimmune hypothyroidism (AIHT) (*r*_g_ = 0.197, *p* = 0.006), and negative correlations with TSH (*r*_g_ = −0.243, *p* = 3.03 × 10^−4^). Further, moderate correlations were observed with type 2 diabetes (T2D) (*r*_g_ = 0.074, *p* = 0.015), prostate cancer (*r*_g_ =0.120, *p* = 0.009), and breast cancer (*r*_g_ =0.127, *p* = 0.007).

Leveraging pleiotropy across these 151 related traits, we used Bayesian non-negative matrix factorization (bNMF) to classify thyroid cancer-associated variants into biologically meaningful clusters. The bNMF analysis identified five mechanistic clusters as providing the best fit to the data (Table 1). Because bNMF jointly factorizes the variant–trait association matrix into a variant loading matrix (W) and a trait loading matrix (H), each cluster is characterized by a specific pattern of contributions from both genetic loci and phenotypic traits. The loci with the highest weights in the W matrix and the traits with the highest weights in the H matrix define the dominant signals within each cluster and therefore provide insight into the putative underlying biological mechanisms (Supplementary Fig. 4, Supplementary Data 2, Table 5). The resulting five clusters include one thyroid dysfunction cluster, one thyroid overactivity cluster, two cancer clusters, and one mixed function cluster.

Clusters 1 (Thyroid Gland Dysfunction) and 2 (Thyroid Overactivity) reflect mechanisms related to thyroid function. Both clusters include decreased TSH and increased free triiodothyronine (FT3), indicating a hyperthyroid state. Consistent with this pattern, our Mendelian randomization analyses demonstrated an inverse causal association between genetically proxied TSH levels and thyroid cancer risk (β_IVW_ = − 0.53, *p* = 4.79E-11; Supplementary Fig. 5, Supplementary Data 2, Table 19), in line with prior studies^19^, providing an independent line of evidence that the negative contribution of TSH within these clusters reflects biologically meaningful variation rather than reverse causation. Thyroid cancer risk alleles linked to Cluster 1 are positively associated with nontoxic multinodular goiter, whereas risk alleles linked to Cluster 2 show a negative association. Both clusters contain variants near *VAV3* and *FOXE1*, genes with established roles in thyroid carcinogenesis via the MAPK and PI3K pathways^61,62^. Additionally, Cluster 1 includes variants near *NRG1*, *INSR*, and *TGFB2*, suggesting interactions between growth factor signaling and epithelial-mesenchymal transition (EMT) in thyroid tumor progression^63,64^. Interestingly, Cluster 1 is also characterized by shortened QRS duration, which may reflect electrocardiogram features associated with overt hyperthyroidism^65–67^. To evaluate whether these clusters reflect broader thyroid disease liability, we performed follow-up analyses using large-scale GWAS of hyperthyroidism (primarily Graves disease). Of 49 independent hyperthyroidism lead variants, 42 were present in our thyroid cancer meta-analysis, and 9 showed nominal replication (*P* < 0.05) with concordant direction of effect, indicating partial genetic overlap (Supplementary Fig. 6a). However, two-sample Mendelian randomization analyses provided no evidence for a causal effect of genetically predicted hyperthyroidism on thyroid cancer risk, with all estimators yielding non-significant results (β_IVW_ = 0.06, *p* = 0.23; Supplementary Fig. 6b, Supplementary Data 2, Table 19).

Thyroid cancer risk alleles linked to Clusters 3 (DNA Damage Response) and 4 (Telomere Maintenance) are strongly associated with cancers and tumorigenic pathways. Both clusters show a strong correlation with leiomyoma of the uterus, but they differ in other specific cancer associations. Cluster 3 is linked to all-cancer risk, prostate cancer, skin cancer, and breast cancer, while Cluster 4 is associated with higher IGF-1 levels, lower eosinophil percentage, and higher neutrophil count. Cluster 3 (DNA Damage Response) contains variants mapped to key tumor suppressors such as *TP53*, *CHEK2*, and *ATM*, all of which play central roles in DNA damage repair pathways^47,68^. *CHEK2* germline mutations have been implicated in breast, colorectal, and thyroid cancers, and loss of function mutations in *CHEK2* with compromised checkpoint-mediated DNA repair, increasing genomic instability^48^. Similarly, both germline and somatic mutations in *ATM* impair the cell cycle checkpoint response, leading to an increased risk of malignancy^69^. Cluster 4 (Telomere Maintenance) features germline variants near *TERT* and TERC/*ACTRT3*, genes previously linked to telomere length regulation in malignancies^70^. Somatic mutations in the TERT promoter are major determinants of increased telomerase activity, with variants linked to multiple cancers, including thyroid cancer^71,72^. Additionally, the cluster includes *STN1*, a component of the CST (CTC1-STN1-TEN1) complex, which is critical for telomere replication and stability^73^, further supporting the cluster’s role in telomere maintenance and oncogenesis.

Thyroid cancer risk alleles linked to Cluster 5 (Mixed Function) are strongly associated with various sets of traits, including heel bone mineral density, QRS and P duration, alkaline phosphatase, triglycerides, and mean arterial pressure. Although this cluster includes variants near *SMAD3*, which is a key transcription factor in TGF-β signaling, with broad roles in development, fibrosis and cancer progression^74^, the lack of common biology with the other top-weighted genes, such as *PRAG1* and *CEP120*, limits biological interpretation, and the cluster remains mechanistically uncharacterized.

To assess the robustness of these mechanistic clusters, we conducted a sensitivity analysis restricted to 73 expert-curated traits (Methods; Supplementary Data 2, Table 4). The resulting four clusters recapitulated the core biological mechanisms observed in the main analysis, including two thyroid-related clusters, one cancer-associated cluster, and one mixed function cluster, with strong overlap in top-weighted traits and loci (Supplementary Data 2, Table 10). For example, Cluster 1, enriched for TSH, FT3, and nontoxic multinodular goiter, corresponds to the original Thyroid Gland Dysfunction cluster. Similarly, the cancer-focused cluster preserved defining features of the DNA Damage Response and Telomere Maintenance cluster, retaining key loci such as *TP53*, *CHEK2*, and *TERT*. Cluster 4 in the sensitivity analysis mirrors the original Mixed Function cluster, showing enrichment for traits including QRS and P duration, pulse pressure, and mean arterial pressure. The consistent emergence of these clusters across distinct input data support the stability of the underlying biological structure and suggest the clustering results are not overly sensitive to trait selection.

### Regulatory processes underlying clusters

To further assess the mechanistic associations with each cluster, we examined the epigenomic enrichment of top loci using cell-type-specific annotations (Fig. 2, Supplementary Data 2, Table 6). We assessed whether variants within each cluster show significant overlap with genes expressed in specific tissues or with chromatin accessibility markers specific to a particular tissue^75^ (Methods).

**Fig. 3:**
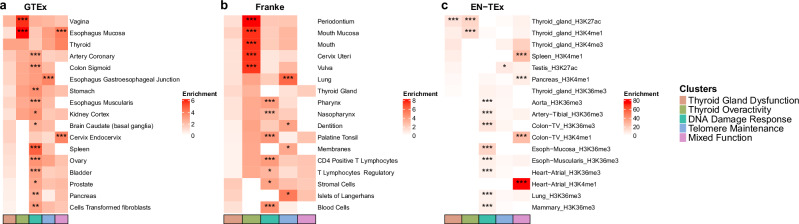
Functional enrichment of cluster-specific thyroid cancer risk loci across tissues, cell types, and biological pathways. Heatmaps display enrichment patterns across top-weighted loci from each cluster across tissues or cell types. Only tissues or cell types with significant enrichment are displayed. For each dataset, the top 17 tissues or cell types are shown, selected based on their average enrichment across clusters with at least one cluster-level enrichment. Statistical significance was assessed using one-tailed permutation tests with 10,000 permutations. Multiple-testing correction was performed using Bonferroni correction within each dataset. **a** Selected tissues from 53 total in GTEx. **b** Selected tissues from 152 total in the Franke dataset. **c** Selected cell-type and histone mark combinations from EN-TEx. *q < 0.1; ***q < 0.001 after Bonferroni correction. Source data are provided as a Source Data file.

The Thyroid Gland Dysfunction cluster showed significant enrichment specifically in thyroid gland chromatin accessibility annotations from the EN-TEx dataset (*P* = 0.019 for Thyroid_gland_H3K27ac)^76^, although there was no significant corresponding enrichment in thyroid-specific gene expression datasets from Franke et al.^77,78^ or GTEx(Fig. 3a–c, Supplementary Data 2, Table 6). The Thyroid Overactivity cluster similarly demonstrated significant enrichment in thyroid gland chromatin features in EN-TEx (*P* < 0.001 for Thyroid_gland_H3K27ac and H3K4me1). Interestingly, while thyroid-specific gene expression enrichment was not observed, this cluster was significantly enriched across multiple non-thyroid tissues, including vagina, esophagus mucosa, and cervix in the GTEx dataset, and periodontal tissue, lung, and cervix uteri in the Franke dataset (Fig. 3a–c).

For the other clusters, enrichment was not restricted to a single tissue type, which is expected given that these pathways regulate cell cycle, metabolic processes, and systemic hormone signaling across multiple tissues. This widespread enrichment pattern suggests that genetic variants in these clusters likely contribute to disease susceptibility through diverse regulatory effects beyond thyroid-specific mechanisms.

Pathway enrichment analysis of genes near index variants in each cluster identified relevant biological pathways (Supplementary Fig. 7, Supplementary Data 2, Table 7). In the Thyroid Gland Dysfunction cluster, key loci, including *FOXE1*, *INSR*, *TGFB2*, *TG*, and *NRG1*, were significantly enriched in pathways related to gland development (*P* = 6.00 × 10^−4^) and regulation of multicellular organismal development (*P* = 0.007). These findings recapitulate their established roles in thyroid proliferation and differentiation^79^. The Thyroid Overactivity cluster demonstrated enrichment for the pathway associated with decreased circulating thyroglobulin concentration, primarily driven by *FOXE1* (*P* = 0.05), further supporting its functional relevance in thyroid hormone biology.

As expected, the DNA Damage Response cluster showed enrichment in pathways associated with DNA damage response, signal transduction by p53 class mediator resulting in cell cycle arrest (*P* = 5.00 × 10^−5^), cellular response to gamma radiation (*P* = 9.76 × 10^−5^), and intrinsic apoptotic signaling pathway (*P* = 0.007), primarily driven by *TP53*, *CHEK2*, and *ATM*^47,68^. Similarly, the Telomere Maintenance cluster was enriched for telomere cap complex (*P* = 0.001), telomeric DNA binding (*P* = 0.013), and TERT-RMRP complex (*P* = 0.05), reinforcing its role in maintaining telomere integrity in tumorigenesis^71^.

For the Mixed function cluster, nuclear mineralocorticoid receptor (MR) binding was the only significantly enriched pathway (*P* = 0.05), driven exclusively by *SMAD3*. Although MR signaling has been implicated in cancer biology, including cellular proliferation, EMT, and metabolic regulation across various cancer types^80^, the enrichment is based on a single gene, and the top-weighted genes in the cluster do not converge on a shared biological pathway. As such, we refrain from assigning a pathway-based label to this cluster. Instead, the cluster appears to be characterized by its leading phenotypic associations, heel bone mineral density, QRS duration, and alkaline phosphatase, without evidence for a shared underlying biological mechanism.

**Fig. 4:**
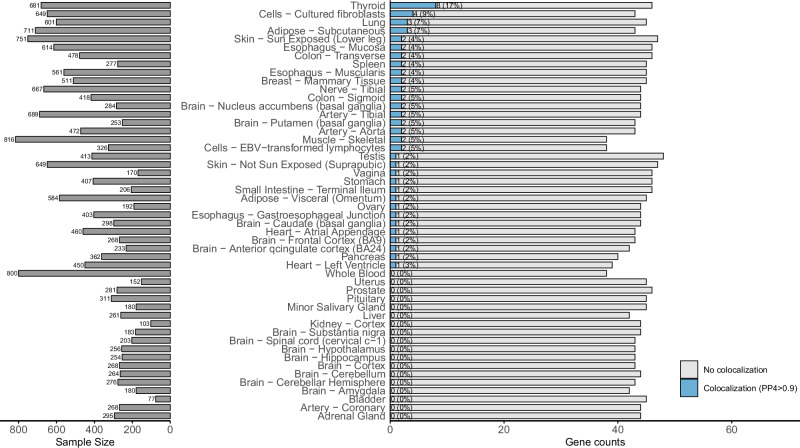
Colocalization analysis of thyroid cancer GWAS loci with GTEx v10 cis-eQTL signals. Left panel: the number of RNA-seq expression samples and genotyped individuals for each tissue in GTEx v10. Right panel: the number of thyroid cancer gene-trait pairs showing strong colocalization evidence, defined as PP4 > 0.9, in each tissue (blue), compared with tested gene-trait pairs without strong colocalization evidence (gray). Percentages in parentheses denote the fraction of tested gene-trait pairs in that tissue showing strong colocalization. For each gene-tissue pair, colocalization analyses included all variants tested within the standard GTEx cis-window, restricted to variants within ±1 Mb of the transcription start site. Source data are provided as a Source Data file.

We investigated whether pleiotropic clusters identified through GWAS represent distinct regulatory mechanisms by evaluating genetic colocalization with tissue-specific eQTL data. Among the 48 unique thyroid cancer genes analyzed, the highest number of colocalized associations was found in thyroid tissue, accounting for 17% of tested gene-trait pairs in thyroid, whereas several other tissues also harbored colocalized eQTLs for a subset of genes (Fig. 4, Supplementary Data 2, Table 13). Genes assigned to specific pleiotropic clusters generally exhibited a more tissue-specific colocalization pattern, predominantly enriched in thyroid, skin, artery, and cultured cells. In contrast, genes not assigned to any specific cluster displayed broader colocalization across multiple tissues (Supplementary Fig. 8). However, differences in PP4 between clustered and unclustered genes were modest and statistically non-significant for most tissues (e.g., thyroid: *p* = 0.052; skin not sun-exposed: *p* = 0.047; cultured fibroblasts: *p* = 0.79; artery-tibial: *p* = 0.20) (Supplementary Fig. 9, Supplementary Data 2, Table 15). Specifically, within the Thyroid Gland Dysfunction and Thyroid Overactivity clusters, 3 out of 9 genes (33%) and 1 out of 2 genes (50%) colocalized specifically with thyroid eQTLs, respectively, whereas no colocalization was observed in adipose-subcutaneous, heart, or nerve-tibial tissues (Supplementary Fig. 10, Supplementary Data 2, Table 14).

### Associations of partitioned polygenic scores (pPGSs) with outcomes

**Fig. 5:**
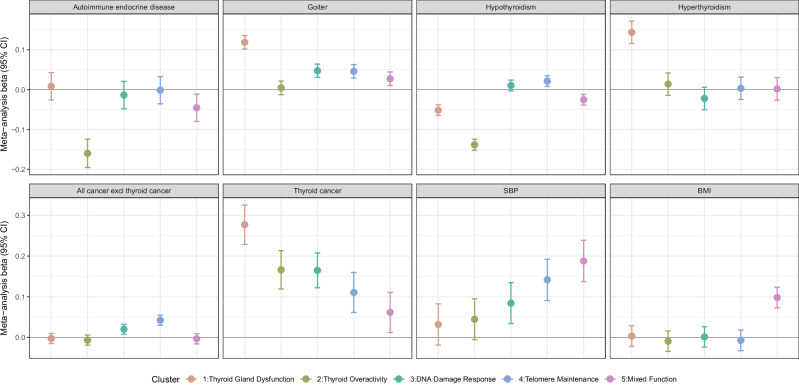
Associations of cluster-specific partitioned polygenic score (pPGS) with thyroid cancer-related outcomes in AllofUs data. Summaries of the associations of each cluster-specific component of the pPGS with autoimmune disease, thyroid disease, cancer, and cardiometabolic traits. Points represent fixed-effects meta-analysis beta estimates per standard deviation increase in the pPGS across ancestry groups, and error bars represent 95% confidence intervals from two-sided association tests. Association testing was performed using logistic regression for binary traits and linear regression for continuous traits, adjusting for age, sex, and the first ten principal components within each ancestry group. Bonferroni correction was applied for the number of outcomes and clusters tested. See full results in Supplementary Fig. 11 and Supplementary Data 2, Tables 8–9. Source data are provided as a Source Data file.

To validate our findings, we examined whether partitioned polygenic scores (pPGS) derived from the top-weighted loci in each cluster were associated with a range of traits in an independent sample. We focused on thyroid cancer-related phenotypes available in the All of Us (AoU) Research Program^81^ (Fig. 5, Supplementary Fig. 11, Supplementary Data 2, Tables 8–9).

The pPGS from the Thyroid Gland Dysfunction cluster showed strongest associations with increased risk of goiter (Beta = 0.119, *P* = 5.91 × 10^−43^), nontoxic goiter (Beta = 0.127, *P* = 4.31 × 10^−39^), and hyperthyroidism (Beta = 0.144, *P* = 9.13 × 10^−24^). Conversely, this cluster was associated with a decreased risk of hypothyroidism (Beta = −0.051, *P* = 1.10 × 10^−13^), consistent with the hyperthyroid phenotype linked to genetic variants within this cluster.

Similarly, the Thyroid Overactivity cluster was significantly associated with a lower risk for hypothyroidism (Beta = −0.138, *P* = 1.79 × 10^−^^85^), autoimmune disease (Beta = −0.042, *P* = 1.02 × 10^−^^6^), and autoimmune endocrine disease (Beta = −0.159, *P* = 1.47 × 10^−18^). In the AoU data browser, Hashimoto thyroiditis comprises the majority of autoimmune endocrine disorder diagnoses.

As expected, our strongest associations that served as a positive control were between all five thyroid cancer clusters and thyroid cancer risk in AoU, with effect estimates ranging from Beta=0.062, *P* = 1.42 × 10^−2^ for the Mixed Function cluster to Beta = 0.277, *P* = 3.30 × 10^−29^ for the Thyroid Gland Dysfunction cluster.

pPGS of cluster-specific variants also exhibit specificity in trait associations; only the DNA Damage Response and Telomere Maintenance clusters were significantly associated with other cancers. These clusters were positively associated with all cancers excluding thyroid cancer (Beta=0.020, *P* = 0.001 for the DNA Damage Response; Beta = 0.043, *P* = 1.87 × 10^−11^ for the Telomere Maintenance cluster). Specific associations were observed for the Telomere Maintenance cluster, which was linked to breast cancer (Beta = 0.049, *P* = 0.0002) and skin cancer (Beta = 0.047, *P* = 5.76 × 10^−6^). These findings suggest that these cancer-related clusters extend beyond thyroid cancer and may influence multiple malignancies.

We next evaluated whether pPGS from thyroid cancer clusters were associated with cardiometabolic traits. We found positive associations between systolic blood pressure and multiple clusters, including the Telomere Maintenance (Beta = 0.142, *P* = 4.59 × 10^−8^) and Mixed function clusters (Beta = 0.188, *P* = 4.06 × 10^−13^). However, only the Mixed Function cluster showed significant associations with various cardiometabolic traits, including diastolic blood pressure (Beta = 0.074, *P* = 2.26 × 10^−5^), BMI (Beta = 0.098, *P* = 2.36 × 10^−14^), obesity (Beta = 0.017, *P* = 6.27 × 10^−4^), type 2 diabetes (T2D) (Beta = 0.024, *P* = 5.74 × 10^−5^), and HbA1c (Beta = 0.010, *P* = 4.85 × 10^−5^). These results suggest that the Mixed Function cluster may reflect shared genetic components between thyroid cancer and metabolic traits, potentially related to obesity-linked pathways, although the precise biological mechanisms remain to be elucidated.

## Discussion

In this study, we conducted a meta-analysis of thyroid cancer GWASs across diverse ancestry groups, identifying 51 genome-wide significant loci, 21 (41%) of which were not previously reported. By increasing effective sample size and incorporating more diverse populations, we identified 57% more loci than the largest prior thyroid cancer GWAS from GBMI^33^. Using pleiotropy dissection, we classified 66 independent thyroid cancer-associated variants across 151 traits into five distinct genetic clusters, each representing biologically meaningful pathways. Our findings suggest that thyroid cancer susceptibility is driven by multiple independent mechanisms, including thyroid gland dysfunction, thyroid overactivity, DNA repair deficiency, telomere maintenance, and mixed physiological function.

Previous studies have demonstrated the utility of pleiotropy dissection in complex diseases by defining genetic subgroups associated with distinct biological mechanisms. Suzuki et al. aggregated T2D GWAS data from over 2.5 million individuals, including >400,000 cases across multiple ancestry groups (~40% non-European), identifying 1289 association signals across 611 loci, including 145 previously unreported loci^28^. Their use of k-means clustering on T2D and 37 cardiometabolic traits revealed eight genetic clusters, enriched in open chromatin regions of pancreatic islets and other cell types, highlighting key regulatory pathways underlying T2D subtypes. Complementary work by Smith et al. applied soft clustering to T2D meta-analysis of 228,000 cases (1.4 million total individuals), integrating 110 T2D-related traits, and identified 12 genetic clusters that largely overlapped with Suzuki et al. clusters and were enriched in specific single-cell regulatory regions^27^. By dissecting pleiotropy among thyroid cancer and related traits for the first time, our study demonstrates that distinct genetic clustering not only captures heterogeneity in thyroid function but also links to broader oncogenic, and metabolic pathways, thereby advancing our understanding of thyroid cancer subtypes and precision medicine approaches.

The Thyroid Overactivity and Thyroid Gland Dysfunction clusters highlight how distinct axes of thyroid function may contribute to thyroid cancer susceptibility through different physiological mechanisms. Both clusters capture TSH-driven thyroid function-related pathways but diverge in their phenotypic associations, particularly with goiter. These clusters recapitulate the potential pleiotropic effects of TSH-associated variants, where Thyroid Overactivity cluster primarily reflects feedback regulation of thyroid hormones on the pituitary, while Thyroid Gland Dysfunction cluster is linked to thyroid growth and an increased risk of thyroid cancer and goiter^19^. Importantly, Mendelian randomization analyses demonstrated a robust inverse association between genetically proxied TSH levels and thyroid cancer risk, consistent with prior studies^19^. This finding provides orthogonal support that the negative loading of TSH within these clusters reflects biologically meaningful variation in thyroid hormone regulation rather than reverse causation driven by disease or treatment effects. In this context, the Thyroid Gland Dysfunction cluster may capture genetic perturbations that alter glandular homeostasis and proliferative signaling under conditions of suppressed TSH, potentially predisposing to neoplastic transformation.

Reeve et al. previously characterized the genetic architecture of AIHT, identifying both thyroid-specific and immune-mediated genetic contributors using linemodels^82^, an alternative method for dissecting pleiotropic effects across pairs of traits into variants that influence both traits from trait-specific effects^30^. Notably, *FOXE1* and *VAV3*, present in both of our thyroid clusters, showed unique effects in AIHT that are absent in broader autoimmune disease in their analysis, consistent with our findings of their thyroid-specificity. In our subtype-specific analysis, *FOXE1* demonstrated consistent associations with differentiated thyroid cancers (PTC and FTC), but not with MTC, whereas *VAV3* was significantly associated only with MTC, likely reflecting limited statistical power. This subtype-specificity may reflect differences in cell-type-specific gene expression and signaling networks, with *FOXE1* crucial for follicular epithelial cell differentiation and function^83^, while *VAV3* is predominantly involved in RET-mediated signaling pathways characteristic of MTC^61,84^. However, further studies are needed to validate the role of these genes across cancer subtypes. Moreover, *FOXE1* and *TG*, two key loci of the Thyroid Gland Dysfunction cluster, overlap with congenital hypothyroidism risk genes identified in the Genomics England clinical panel^85^, reinforcing their roles in thyroid development and thyroid hormone production. Moreover, several genes involved in both AIHT and TSH regulation (*DIRC3*, *NRG1*, *INSR*, and *VAV3*) in Reeve et al align with the Thyroid Gland Dysfunction cluster. These findings suggest that genetic variation in thyroid-related pathways may influence both autoimmune thyroid diseases and thyroid cancer susceptibility, providing insights into shared molecular mechanisms underlying thyroid dysfunction and tumorigenesis.

The Thyroid Gland Dysfunction cluster is also characterized by associations with shortened QRS duration^65–67^. Although even mildly altered thyroid status can affect heart rhythm^86^ and rate^87^, the frequent occurrence of cardiac manifestations in hyperthyroid patients may result from thyrotoxicosis itself, pre-existing heart disease exacerbated by hyperthyroidism, or specific hyperthyroidism-induced cardiac abnormalities^88^. While QRS duration is less sensitive to heart rate than the QT interval^89^, these associations may suggest a potential influence of thyroid hormone dysregulation on cardiac conduction. However, as GWAS of these ECG traits used in this study are not adjusted for heart rate, the mechanisms underlying these associations remain unclear and warrant further investigation. In our evaluation of whether the thyroid-related clusters reflect liability to clinical hyperthyroidism, we observed partial overlap between hyperthyroidism risk loci and thyroid cancer–associated variants; however, Mendelian randomization analyses did not support a causal effect of genetically predicted hyperthyroidism on thyroid cancer risk. These findings suggest that the shared loci likely represent pleiotropic genetic architecture influencing thyroid hormone regulation and cancer susceptibility through partially independent biological pathways, rather than a direct etiologic effect of hyperthyroidism on malignancy.

Beyond TSH-related mechanisms, our discovery of cancer-related clusters (DNA Damage Response and Telomere Maintenance) suggests that thyroid cancer shares fundamental tumorigenic mechanisms with other malignancies. These clusters reflect biologically distinct processes, including genomic instability and replicative immortality, both hallmarks of cancer. The DNA Damage Response cluster includes top loci associated with DNA repair pathways, such as *ATM*, *CHEK2*, and *TP53*. ATM and ATR kinases phosphorylate p53 following DNA damage, regulating cell cycle arrest, DNA repair, and apoptosis; thus, mutations in this cluster may increase genomic instability and predispose individuals to aggressive thyroid cancer subtypes^90^. Similarly, the enrichment of *TERT*-associated loci in the Telomere Maintenance cluster underscores the role of telomere length regulation in thyroid tumorigenesis. Genetic predisposition to telomere maintenance is thought to facilitate continuous cell proliferation and the accumulation of oncogenic mutations, a well-established feature of aggressive thyroid cancers^71^. Notably, these mechanistic overlaps reflect epidemiological patterns of increased co-occurrence between thyroid cancer and other malignancies, particularly breast and kidney cancers, which can be explained by shared genetic and environmental risk factors and medical surveillance^91,92^. While these clusters may not immediately alter clinical management of thyroid cancer, they offer valuable insights into the underlying biological heterogeneity of the disease. Specifically, they may help identify subsets of patients at higher risk of aggressive disease, inform drug repurposing efforts targeting DNA repair or telomerase pathways, and guide future studies on common mechanisms across multiple cancer types. More broadly, these findings illustrate how pleiotropic clustering can reveal latent axes of dysregulation that contribute to disease pathogenesis and may eventually support precision oncology approaches.

The Mixed Function was associated with several cardiometabolic and inflammatory markers, suggesting that pathways related to metabolic dysregulation and inflammation may contribute to thyroid cancer susceptibility. However, pathway enrichment for nuclear MR binding was largely driven by a single implicated gene, and therefore should be interpreted cautiously. MR is expressed in normal thyroid tissues and PTC cells, where its activation by aldosterone induces inflammatory responses such as interleukin-6 expression and modulates thyroid-specific genes^93^. Furthermore, MR expression progressively declines in more aggressive thyroid cancer histotypes, raising the possibility that MR-related signaling may be relevant to disease biology in some contexts^80,93^. Nonetheless, the extent to which this signal reflects a broader cluster-level mechanism versus a locus-specific effect remains unclear. Future work incorporating thyroid-relevant tissue and single-cell functional annotations will be important to determine whether the Mixed Function cluster reflects a shared cardiometabolic/inflammatory mechanism.

Our external validation analysis demonstrated that pPGSs from thyroid, cancer, and cardiometabolic genetic clusters were significantly associated with risks of thyroid disease, cancers, and cardiometabolic conditions, consistent with the key traits identified within each cluster. These findings suggest that genetic clustering may provide a framework for characterizing biological heterogeneity among individuals with genetic predisposition to thyroid cancer. For instance, somatic mutations in the *TERT* promoter are associated with poor prognosis and loss of radioiodine (RAI) avidity when co-occurring with *BRAF V600E* mutations^94,95^. However, it remains unclear whether germline TERT variations confer similar clinical implications. In cases of RAI-refractory differentiated thyroid cancer, multi-kinase inhibitors such as lenvatinib and sorafenib have been approved for treatment. These agents are typically employed in advanced RAI-refractory disease when other options are limited^96–98^. Similarly, *TP53* mutations are frequently observed in ATC, the most aggressive thyroid cancer subtype^99^. While *TP53* mutations are often linked to treatment-resistant cancers, they have also been proposed as potential biomarkers of immunotherapy responsiveness, as p53 loss has been linked to increased tumor mutational burden, which may enhance response to immune checkpoint inhibitors^100–102^. However, the predictive value of *TP53* mutations for immunotherapy response in thyroid cancer remains uncertain^103^. Although our findings support the etiologic relevance of genetically informed subclassification, direct clinical utility for risk stratification, treatment selection, or precision oncology remains to be established. Future studies integrating germline genetic clustering with tumor molecular profiles, treatment exposures, and longitudinal clinical outcomes will be needed to determine whether patients stratified by genetic clusters exhibit differential prognosis or responses to targeted therapies.

There are several limitations to this study. First, we were unable to assess the impact of genetic clusters across thyroid cancer histologic types or by stage at diagnosis, which are associated with prognosis and treatment response. Consequently, some observed associations may reflect vertical pleiotropy and reverse causation. For instance, thyroidectomy, a standard treatment for thyroid cancer, induces hypothyroidism, necessitating levothyroxine therapy to suppress TSH levels, potentially leading to iatrogenic hyperthyroidism. This clinical intervention could influence phenotypic associations captured by our thyroid-related clusters. Second, heterogeneity in screening practices and diagnostic criteria across countries, cohorts, and calendar years^104,105^ may contribute to variability in the direction and magnitude of observed associations. Our findings highlight subtype-specific genetic associations, underscoring the need for subtype-stratified studies to further elucidate these genetic relationships. Third, we did not account for prior thyroid disease diagnoses, hormone replacement therapy, or thyroid cancer treatments, all factors that could impact genetic associations^22^. While our results suggest that certain genetic clusters may inform differential outcomes or treatment strategies, their direct clinical application requires further validation. In parallel to our present study, the GBMI has explored the genetic architecture of thyroid diseases and developed clinically relevant polygenic risk score discriminating thyroid cancer and benign thyroid nodules, which may aid in improving diagnosis accuracy of thyroid cancer^106^. Fourth, results from matrix factorization approaches such as bNMF can be sensitive to analytic design choices, including trait selection, handling of missing data, and normalization of input summary statistics. To evaluate the robustness of our findings, we conducted several complementary sensitivity analyses. Specifically, we assessed (i) the impact of trait selection by repeating clustering using a restricted set of 73 expert-curated traits directly relevant to thyroid biology and cancer, which reproduced the primary thyroid-related, cancer-related, and mixed physiological clusters (Supplementary Data 2, Table 10); (ii) the influence of missing data handling—required because standard bNMF does not accommodate incomplete matrices—by implementing a proxy-based imputation strategy, which yielded highly consistent cluster architecture and locus assignments (Supplementary Data 2, Table 11); and (iii) the effect of alternative normalization procedures for the variant–trait association matrix, observing stable cluster structure across scaling approaches (Supplementary Data 2, Table 16, Supplementary Fig. 12). These analyses indicate that the inferred pleiotropic architecture is robust to key analytic decisions inherent to bNMF-based clustering. Fifth, we used bNMF for variant clustering; however, alternative methods such as independent component analysis^107^ or linemodels^82^ could yield different partitions of genetic signals. Further validation is needed to assess cluster robustness across analytical approaches.

Our findings demonstrate the value of cross-trait genetic associations in elucidating distinct molecular pathways underlying thyroid cancer. Future research should focus on integrating functional genomics, transcriptomics, and clinical interventions to further refine genetically informed strategies for thyroid cancer management and personalized treatment.

## Methods

### Meta-analysis of thyroid cancer

Following analysis plans outlined previously^33^, each study conducted a GWAS of thyroid cancer using logistic mixed model with SAIGE^108^ or whole genome regression with REGENIE^109^ (Supplementary Data 2, Table 1). For the Korean Cancer Prevention Study-II Biobank (KCPS2), we performed GWAS using individual-level genotype and phenotype data. For the other contributing biobanks, we used publicly available GWAS summary statistics or summary statistics generated by the corresponding studies. Phenotype definitions for thyroid cancer varied across contributing biobanks, including diagnoses based on ICD-10 codes, phecodes, or self-reported data (Supplementary Data 2, Table 1). Thus, the analyses included both prevalent and incident thyroid cancer cases, and were not restricted to first cancer occurrences. We conducted a fixed-effect meta-analysis of thyroid cancer using inverse-variance weighting with METAL,^110^ combining GWAS summary statistics from two meta-analyses (the GBMI meta-analysis^33^ and a separate meta-analysis of FinnGen R12^111^ and MVP^37^) and four individual biobanks (KCPS2^112,113^, Latvian National Biobank^114^, Penn Medicine BioBank, and Genomic Health Initiative) (Supplementary Data 2, Table 1). The resulting effective sample size is approximately 64,240, which is more than double that of the next largest prior GWAS of thyroid cancer.^33^ All KCPS2 participants provided written informed consent approved from ethics committees of Yonsei University.

To assess heterogeneity across the six sets of GWAS summary statistics contributing to our meta-analysis, we calculated Cochran’s Q statistics. We retained variants present in at least one of the GWAS in this meta-analysis for locus discovery. Given the limitations of high-resolution fine-mapping methods in the context of meta-analyses particularly when performed using different genotyping and imputation techniques, we adopted a conservative strategy for defining lead variants. Specifically, we flagged only the most significantly associated variant within each confidently defined, linkage disequilibrium (LD)-independent locus. LD independent associations were determined as follows: starting with the most significant association with *p* < 5 × 10^−8^ on each chromosome, we scanned a ± 2 Mb window around each genome-wide significant signal. A dynamic LD threshold was then applied, defined as T = min (0.1, r_5_) where *r*_5_ corresponds to the *r*^2^ value at which the expected residual chi-square statistic equals 5.0. Secondary associations within the window were retained only if they were genome-wide significant and had *r*^2^ < T with any more significant signal. In instances where the lead association was exceptionally strong (resulting in T < 0.02), we excluded secondary signals within ±1 Mb to mitigate the risk of false independence due to instability in low r² estimates. LD was estimated using the Finnish LD reference panel (SISu v4.2), for which the autoreport framework is calibrated and optimized.

To assess robustness to LD reference choice and signal definition strategy, we conducted sensitivity analyses using standard fixed-threshold LD clumping with the 1000 Genomes Phase 3 ALL (cosmopolitan) reference panel under both conventional (r² = 0.1) and stringent (r² = 0.005) thresholds. We additionally reran downstream clustering analyses using lead variants defined under the stringent fixed-threshold approach. These sensitivity analyses yielded qualitatively similar locus-level and clustering results, indicating that our primary conclusions are robust to the choice of LD reference panel and clumping strategy (Supplementary Data 2, Tables 17–18).

A locus was classified as previously reported if it contained a lead variant within a ± 500 kb flanking window of a known association for the same Experimental Factor Ontology (EFO) term in the Open Target Genetics database (release 22.09)^115^. Otherwise, it was categorized as previously unreported (Supplementary Data 2, Table 2). To comprehensively identify prior associations, we performed an exhaustive search in the GWAS Catalog and then excluded loci from the list of previously unreported hits if they were reported in the GWAS catalog but not in OpenTargets. Additionally, as FinnGen R12^111^ and MVP^37^ results were not available in the Open Target Genetics database or the GWAS Catalog at the time of evaluation for previously unreported associations, we additionally excluded loci where a ± 500 kb flanking window contained any previously reported associations from FinnGen or MVP.

To assess heterogeneity of germline risk across thyroid cancer subtypes, we used subtype-specific thyroid cancer GWAS summary statistics from FinnGen R12^111^ to compare effect sizes for 66 lead variants identified in the multi-ancestry meta-analysis. Subtype-specific summary statistics were available for papillary thyroid carcinoma (PTC), follicular thyroid carcinoma (FTC), and medullary thyroid carcinoma (MTC); summary statistics for anaplastic thyroid carcinoma (ATC) were not available, likely due to the small number of cases. Effect sizes (log-odds ratios) were compared across subtypes to evaluate concordance and heterogeneity of genetic associations. We quantified overlap in subtype diagnoses to characterize co-diagnosis across histologies. Given the observed co-diagnoses, we conducted sensitivity analyses restricting to histologically exclusive PTC (*N* = 2284) and FTC cases (*N* = 212), defined as individuals with only a single histological subtype diagnosis (Supplementary Fig. 2).

### Defining clusters of thyroid cancer index variants using bNMF

To classify thyroid cancer-associated variants into biologically meaningful subgroups, we applied Bayesian non-negative matrix factorization (bNMF)^26,116^. The clustering was based on GWAS summary results for the independent thyroid-cancer index variants across 151 phenotypes, including 73 expert curated traits associated with risk of thyroid cancer (Thyroid disease, Autoimmune disease, Cancer, Cardiometabolic, Hormone measurement), alongside 78 additional traits linked to categories associated with thyroid cancer variants (Anthropometry, Lung, Mental disease, Biomarker, Hematological traits, Kidney, Reproductive system, Genomic measurement). We prioritized multi-ancestry GWAS for ancillary trait summary statistics; however, when unavailable for a given trait, European-based GWASs were used. Linkage disequilibrium score regression (LDSC)^117^ was applied to estimate cross-trait genetic correlations (r_g_) between our thyroid cancer meta-analysis and 151 clustering phenotypes.

We applied a modified bNMF pipeline^26,27,118^. Detailed descriptions of the pipeline are provided in Supplementary Data 1. In the NMF literature^118^, latent components are often referred to as ‘features’; here, we use the term ‘cluster’ to reflect the biological interpretation of these components as groups of variants and traits with shared genetic associations. Traits were excluded if their median sample size was below 5000 or if their minimum P value for the final variant set did not meet Bonferroni significance (Pmin > 0.05/66 variants). Because the bNMF framework is designed to identify latent clusters representing distinct patterns of variant-trait associations, inclusion of highly correlated traits can introduce redundancy and effectively over-weight a single underlying biological signal, biasing the factorization. Accordingly, we removed highly correlated traits based on Pearson correlation coefficient (>0.80) calculated from GWAS summary statistics across index variants, retaining the trait with the strongest variant–trait association to preserve biological information, while minimizing redundancy-driven distortion of cluster weights. This resulted in a final set of 100 traits (Supplementary Data 2, Table 5). The cluster membership cutoff (1.057017) was determined using a long-tail (‘elbow’) analysis of clustering weight deltas and was applied consistently across cluster definition, enrichment analysis, and pPGS construction; this threshold affects interpretative labeling of strongly contributing variants but does not alter the underlying bNMF factorization or cluster inference (Supplementary Data 1).

We generated standardized effect sizes for variant trait associations from GWAS by dividing the estimated regression coefficient beta by the standard error, which had been aligned to the thyroid cancer risk-increasing alleles. To account for differences in sample size across studies and enable a more uniform comparison of phenotypes across studies, we scaled the standardized effect sizes by the square root of the study size, as estimated by mean sample size across all SNPs, forming the variant-trait association matrix **Z** (66 variants × 100 traits). This standardization strategy follows established practice in the literature applying NMF to GWAS summary statistics, including the original bNMF application to multi-trait genetic data^26,27^, where sample-size-adjusted Z-scores were used to construct the variant-trait association matrix (Supplementary Data 1). To assess robustness to the choice of normalization, we performed a sensitivity analysis comparing raw Z-scores and sample-size-adjusted Z-scores (Supplementary Data 2, Table 16, Supplementary Fig. 12).

Missing variant–trait associations were replaced with zero. For sensitivity analysis, we imputed missing associations using z-scores from proxies (LD r^2^ > 0.5) where possible and otherwise used the median observed standardized effect size for the trait (Supplementary Data 2, Table 11).

The bNMF algorithm was implemented in RStudio and executed for 10,000 iterations with varying initial conditions. The most frequently observed K across 50 chains of 10,000 iterations was selected as the final model. To assess the robustness of the clustering results, we conducted a sensitivity analysis restricting the phenotypic input to the 73 expert-curated traits (reduced to 50 traits after filtering) (Supplementary Data 2, Table 10). Convergence of the bNMF algorithm was assessed by monitoring the objective function and automatic relevance determination (ARD) relevance weights across iterations. In the primary run, both quantities stabilized well before the pre-specified 10,000-iteration limit, consistent with prior ARD-NMF implementations^116^ (Supplementary Fig. 13a). Across all random initializations, convergence was achieved substantially earlier than the maximum iteration cap, indicating that the 10,000-iteration limit was conservative and sufficient (Supplementary Fig. 13b).

### Enrichment of thyroid cancer associations for cell-type-specific regions of open chromatin within clusters

To assess whether cluster-specific variants exhibit significant overlap with cell-type-specific regulatory elements, we computed excess overlap as defined in a previous study^119^. Excess overlap quantifies whether two binary annotations co-occur more often than expected by chance. Specifically, for two binary annotation vectors (annot1 and annot2) across *M* variants, it is calculated as:

$${excess}\,{overlap}({annot}1,{annot}2)=\frac{\frac{1}{M}{\sum }_{j=1}^{M}{{annot}1}_{j}*{{annot}2}_{j}}{(\frac{1}{M}{\sum }_{j=1}^{M}{{annot}1}_{j})*(\frac{1}{M}{\sum }_{j=1}^{M}{annot}{2}_{j})}$$

For each cell-type annotation, we define annot1 as a binary vector across the *M* variants, where each entry takes the value 1 if the corresponding variant is a thyroid cancer index variant for a given cluster, and 0 otherwise. Index variants were assigned to a cluster if their cluster weight was above 1.057, a threshold determined to maximize the signal-to-noise ratio, as previously described^26^. Similarly, annot2 is a binary vector indicating whether each of the *M* variants is annotated as enriched in the given cell-type (1) or not (0). This calculation is mathematically equivalent (up to a simple transformation) to computing the Pearson correlation between the two binary annotations.

We applied this metric to five binary cell-type annotation datasets used in LD Score regression with Specifically Expressed Genes (LDSC-SEG)^75^. Gene expression-based annotations (GTEx^120^, Franke^77,78^, ImmGen^121^) were generated by identifying genes with highly tissue- or cell-type-specific expressions (top 10% by specificity metrics) and defining SNP annotations as windows (±100 kb) around these genes. Chromatin-based annotations from Roadmap^122,123^ and EN-TEx datasets^76^ were defined using experimentally identified peaks for DNase I hypersensitivity and activating histone marks (H3K27ac, H3K4me3, H3K4me1, H3K9ac and H3K36me3). For each cluster of thyroid cancer index variants, we mapped them to the full set of *M* SNPs covered by each LDSC-SEG annotation dataset and computed the excess overlap with each cell-type-specific annotation.

The statistical significance of annotations in each cluster was assessed using a permutation test. For each cluster, we permuted annot1 values (thyroid cancer index variant indicators) and recalculated the excess overlap using shuffled labels. After 10,000 permutations, we compared the observed excess overlap to the permuted background using a one-tailed test to determine the significance of each annotation. We corrected for multiple tests and considered statistically significant enrichment at q-value thresholds of 0.1 and 0.001, using Bonferroni correction (for 53 tissues in GTEx, 152 tissues in Franke, 292 immune cell types in ImmGen, 396 in Roadmap, 93 in EN-TEx).

### Pathway enrichment analysis

To identify biological pathways enriched in each genetic cluster, we used gProfiler^124^ (g:GOSt) (https://biit.cs.ut.ee/gprofiler/gost) to test for KEGG, WikiPathways, or REACTOME pathway enrichment. g:GOSt conducts functional profiling of gene lists by analyzing various types of biological evidence, performing statistical enrichment analysis to identify over-representation of information from Gene Ontology (GO) terms, biological pathways, regulatory DNA elements, human disease gene annotations, and protein-protein interaction networks.

The functional enrichment of the input gene list is evaluated using a cumulative hypergeometric test, evaluating multiple functional terms simultaneously for each gene list. To control for false positives, we applied g:SCS (Set Counts and Sizes) multiple testing correction, the default method in g:GOSt^125^. g:SCS accounts for overlapping functional terms, making it more conservative than Benjamini-Hochberg False Discovery Rate but less stringent than Bonferroni correction.

### Colocalization analysis of thyroid cancer GWAS loci with eQTLs

For each risk locus identified in the thyroid cancer GWAS, we performed colocalization analyses to assess whether GWAS signals share causal variants with expression quantitative trait loci (eQTL) from GTEx tissues. Bayesian colocalization analyses were conducted using coloc^126^, integrating thyroid cancer GWAS summary statistics with cis-eQTL datasets from GTEx v10 across 50 human tissues. For each gene–tissue pair, we included all variants tested within the standard GTEx cis window, restricting analyses to variants located within ±1 Mb of the transcription start site.

We initially mapped 66 independent thyroid cancer GWAS loci to 53 unique genes, assigning each locus to the gene with the most severe predicted functional consequence based on the Ensembl Variant Effect Predictor (VEP) annotations provided by FinnGen. Specifically, variants located within coding sequences or introns were mapped directly to the corresponding annotated gene, while variants located outside annotated genes were assigned to predicted gene loci (LOC identifiers). Among these 53 genes, three (*LOC105375746, LOC107984003, LOC107984411*) represented predicted genes without corresponding Ensembl annotations, and two (*LINC02428, TERC*), though annotated in the GTEx portal, lacked cis-eQTL associations in all tissues, likely due to low or absent expression. Consequently, 48 genes were carried forward for colocalization testing. For each of these 48 thyroid cancer-associated genes across the 50 GTEx tissues, we calculated posterior probabilities of colocalization (PP4), with PP4 > 0.9 considered strong evidence for shared causal variants influencing both thyroid cancer risk and gene expression. We further summarized colocalization frequencies across five previously defined thyroid cancer mechanistic clusters and performed two-sample Wilcoxon tests to compare PP4 distributions between clustered and unclustered genes within each tissue.

### Cluster-specific partitioned polygenic score analyses in All of Us

To validate the thyroid cancer genetic clusters, we constructed partitioned polygenic scores (pPS) and tested their associations with potential thyroid cancer-related traits in the All of Us Research Program (AoU) database^81^. Phenotypic data were derived from two primary domains in AoU: the conditions domain, which captures medical diagnoses and health outcomes based on standardized clinical codes (e.g., ICD, SNOMED), and the measurement domain, which includes quantitative clinical values resulting from examinations or tests. For the All of Us Research Program, informed consent was obtained for all the participants in person or through an eConsent platform. The protocol was reviewed by the Institutional Review Board of the All of Us Research Program.

The pPGS were calculated as weighted sums of genetic variants within each cluster, with weights derived from the bNMF clustering results. No LD pruning or shrinkage methods were applied during pPGS construction because the goal of the pPGS was not prediction optimization but biological interpretation of cluster-specific genetic profiles. Only variants with cluster weights above 1.057017 were included in the pPGS^26^. The pPGS were standardized to have a mean of zero and unit variance and analyzed separately for four ancestry groups (AMR, AFR, EUR, and EAS) in AoU.

Within each ancestry, association testing was performed using logistic regression for binary traits and linear regression for continuous traits, adjusting for age, sex, and the first ten principal components (PCs). A fixed-effects inverse-variance-weighted meta-analysis was conducted to combine ancestry-specific estimates, with Bonferroni correction applied for the number of outcomes and for both the number of outcomes (20) and the number of clusters examined (5 × 20).

### Mendelian randomization analysis of TSH, hyperthyroidism, and thyroid cancer

We conducted two-sample Mendelian randomization (MR) analyses to evaluate potential causal relationships between thyroid function–related traits and thyroid cancer risk. Thyroid cancer outcome summary statistics were derived from the meta-analysis performed in the present study.

For circulating thyroid-stimulating hormone (TSH), genetic instruments were obtained from the ThyroidOmics consortium^18^, which provides genome-wide association summary statistics for TSH measured in individuals without diagnosed thyroid disease. Restricting to euthyroid individuals reduces potential bias related to disease status or treatment. For hyperthyroidism (E4_THYTOXGOITDIF; primarily Graves disease), summary statistics were obtained from a FinnGen-MVP-UK Biobank meta-analysis^37,111,127^. For both exposures, we used the conditionally independent genome-wide significant lead variants reported in the original GWAS publications as genetic instruments. Variants were harmonized with thyroid cancer summary statistics to ensure consistent allele alignment and effect direction.

Causal estimates were primarily obtained using the inverse-variance weighted (IVW) method under a multiplicative random-effects model. Sensitivity analyses were conducted using MR-Egger regression, weighted median, and weighted mode estimators to assess robustness to potential violations of instrumental variable assumptions. Heterogeneity across instruments was evaluated using Cochran’s Q statistic, and directional pleiotropy was assessed using the MR-Egger intercept test. To further detect and correct for horizontal pleiotropy, we applied the MR-PRESSO framework^128^ to identify outlier variants and repeated analyses after their exclusion. For hyperthyroidism, we additionally evaluated locus-level overlap with thyroid cancer by examining concordance of effect directions and nominal significance of lead variants. Full instrument details and sensitivity results are provided in Supplementary Fig. 5–6 and Supplementary Data 2, Table 19.

### Statistics and reproducibility

All statistical analyses were conducted using established methods described in the corresponding sections of the manuscript. Genome-wide association analyses within contributing biobanks were performed using SAIGE or REGENIE, followed by fixed-effects inverse-variance-weighted meta-analysis. Unless otherwise specified, all statistical tests were two-sided. Multiple-testing correction procedures were applied where appropriate and are described in the relevant Methods and figure legend sections.

No statistical method was used to predetermine sample size. Sample sizes were determined by the availability of existing biobank and consortium datasets included in the meta-analysis and downstream analyses. No data were excluded from the primary analyses unless explicitly stated. Sensitivity analyses using alternative LD clumping strategies, trait selection procedures, and imputation approaches yielded qualitatively similar results.

The experiments were not randomized because this study analyzed existing human genetic association summary statistics and biobank-derived observational data. The investigators were not blinded to allocation during analyses and outcome assessment because no experimental intervention or group allocation was performed.

### Reporting summary

Further information on research design is available in the Nature Portfolio Reporting Summary linked to this article.

## Supplementary information

Supplementary Information

Description of Additional Supplementary Files

Supplementary Data 1

Supplementary Data 2

Reporting Summary

Transparent Peer Review file

## Source data

Source Data

## Data Availability

The meta-analysis GWAS summary statistics are publicly available at 10.5281/zenodo.18932445. Data sources for other publicly available GWAS summary statistics are available in Supplementary Data 2, Table 4. Source data are provided with this paper.

## Code availability

The code used for meta-analysis and pleiotropy dissection has been deposited in Zenodo at 10.5281/zenodo.20433102 and is maintained at https://github.com/yonhojee/thyroid_cancer_bnmf. Analyses were conducted using SAIGE (v0.29.5), METAL (v2020-05-05); autoreporting, https://github.com/FINNGEN/autoreporting (v2020-10-26); LDSC (v2015-02-12); bNMF clustering, https://github.com/gwas-partitioning/bnmf-clustering (v1); g:Profiler; coloc (v6); TwoSampleMR (v0.7); MR-PRESSO; R; and RStudio.
