# Supplementary material for: Genetic drivers of etiologic heterogeneity in thyroid cancer
Source: Nat Commun. 2026 Jul 10;17:8515. doi: 10.1038/s41467-026-75111-8 (PMC13483233; doi:10.1038/s41467-026-75111-8)
Supplement: Supplementary file 1 [file 41467_2026_75111_MOESM1_ESM.pdf]

Supplementary Information

Supplementary Figures

Supplementary Figure 1. Manhattan plot for the meta-analysis GWAS of thyroid cancer **(a)** and comparisons of the frequency of thyroid cancer risk allele versus the effect sizes for all 51 thyroid cancer genetic loci **(b)**. Novel thyroid cancer loci are highlighted in red.

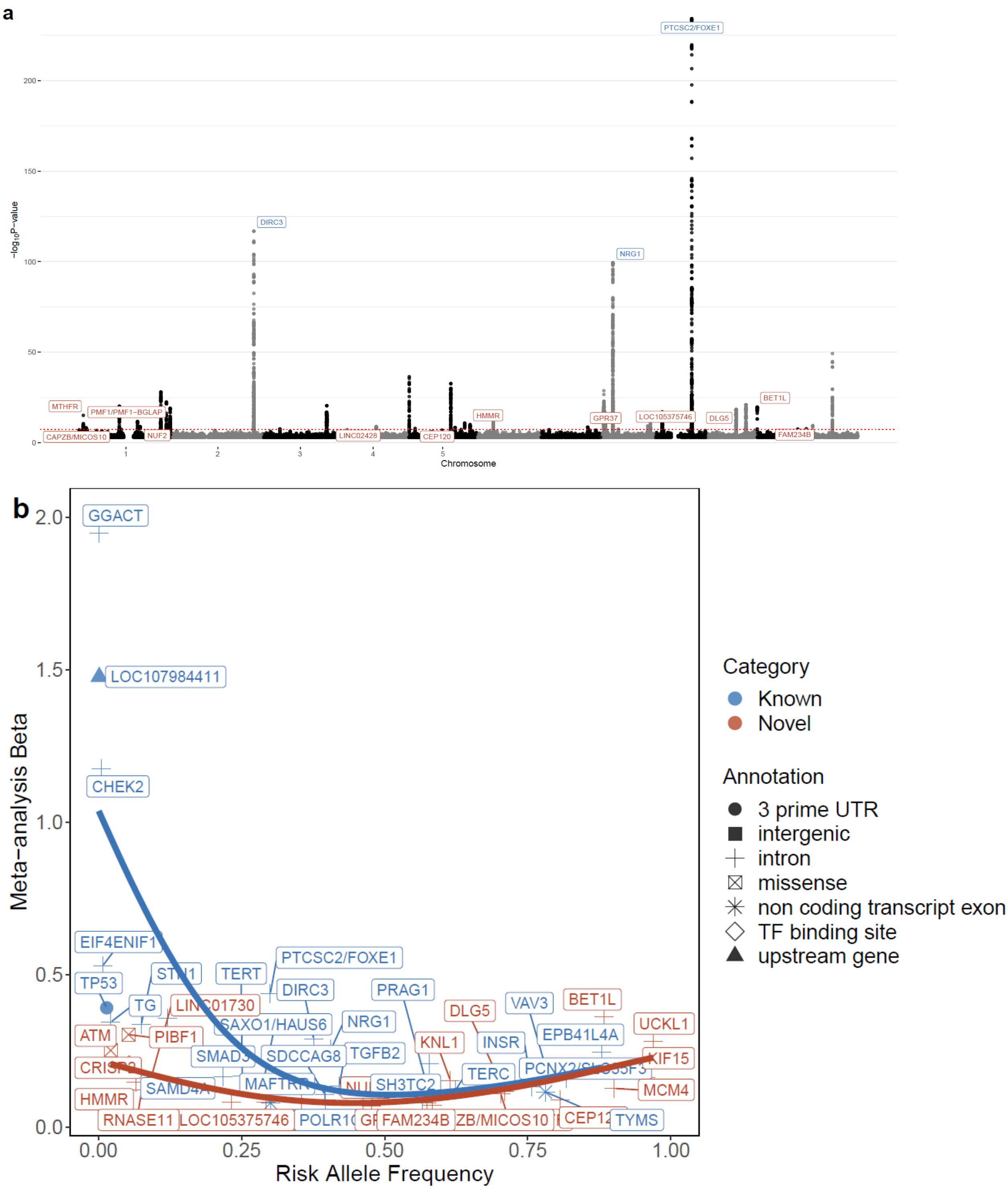

Supplementary Figure 2. Heterogeneity of GWAS Loci Across Thyroid Cancer Subtypes in FinnGen R12. **a.** Number of thyroid cancer cases across subtypes in FinnGen. UpSet plot illustrating the intersection of thyroid cancer subtypes defined in FinnGen. The majority of individuals were diagnosed exclusively with papillary thyroid carcinoma (PTC; n = 2,334), followed by follicular thyroid carcinoma (FTC; n = 385), medullary thyroid carcinoma (MTC; n = 48), and anaplastic thyroid carcinoma (ATC; n = 28). A smaller subset of individuals had multiple subtype diagnoses, including concurrent PTC and FTC (n = 165), PTC and MTC (n = 16), and PTC and ATC (n = 9). **b.** Subtype-specific effect sizes for thyroid cancer risk variants restricted to "pure PTC" (N=2,284) and "pure FTC" cases (N=212) (i.e., excluding individuals with co-diagnoses across histological subtypes) in FinnGen R12. Heatmap of beta coefficients for 51 loci across thyroid cancer subtypes in FinnGen: papillary (PTC), follicular (FTC), and medullary (MTC). Each column represents a locus, and rows correspond to subtype-specific effect sizes. Variants are clustered by similarity in effect profiles. Effect sizes represent log-odds ratios from subtype-specific GWAS analyses in FinnGen. *P < 0.05 (nominal significance); **P < 5×10^{-8} (genome-wide significance threshold).

a

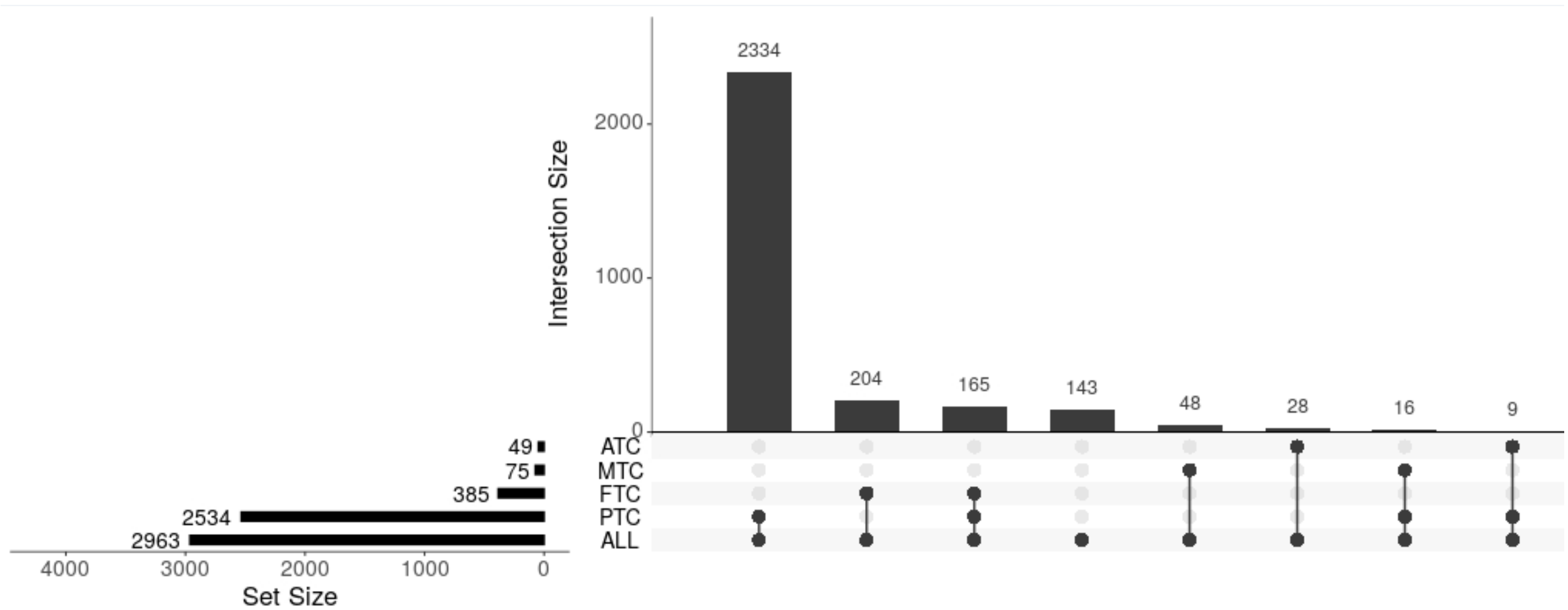

b

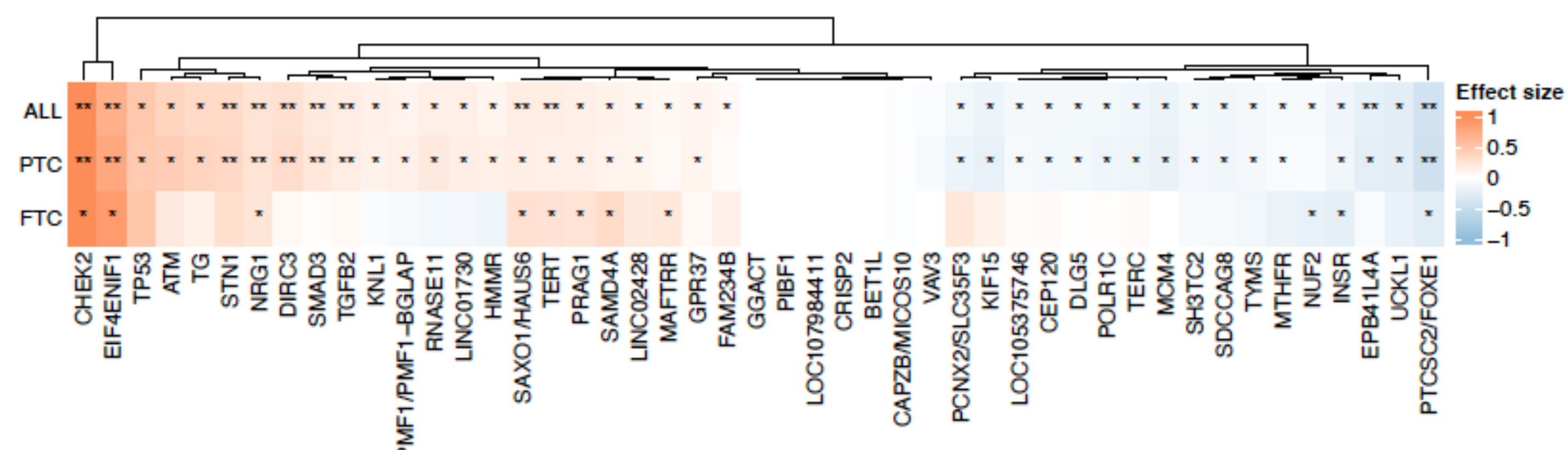

Supplementary Figure 3. Genetic correlation estimates between thyroid cancer and 151 clustering phenotypes. Genetic correlations were estimated using linkage disequilibrium score regression (LDSC). Only correlations reaching nominal significance (two-sided P < 0.05) are displayed. Full genetic correlation results are shown in Supplementary Data 2, Table 12. ThC=thyroid cancer.

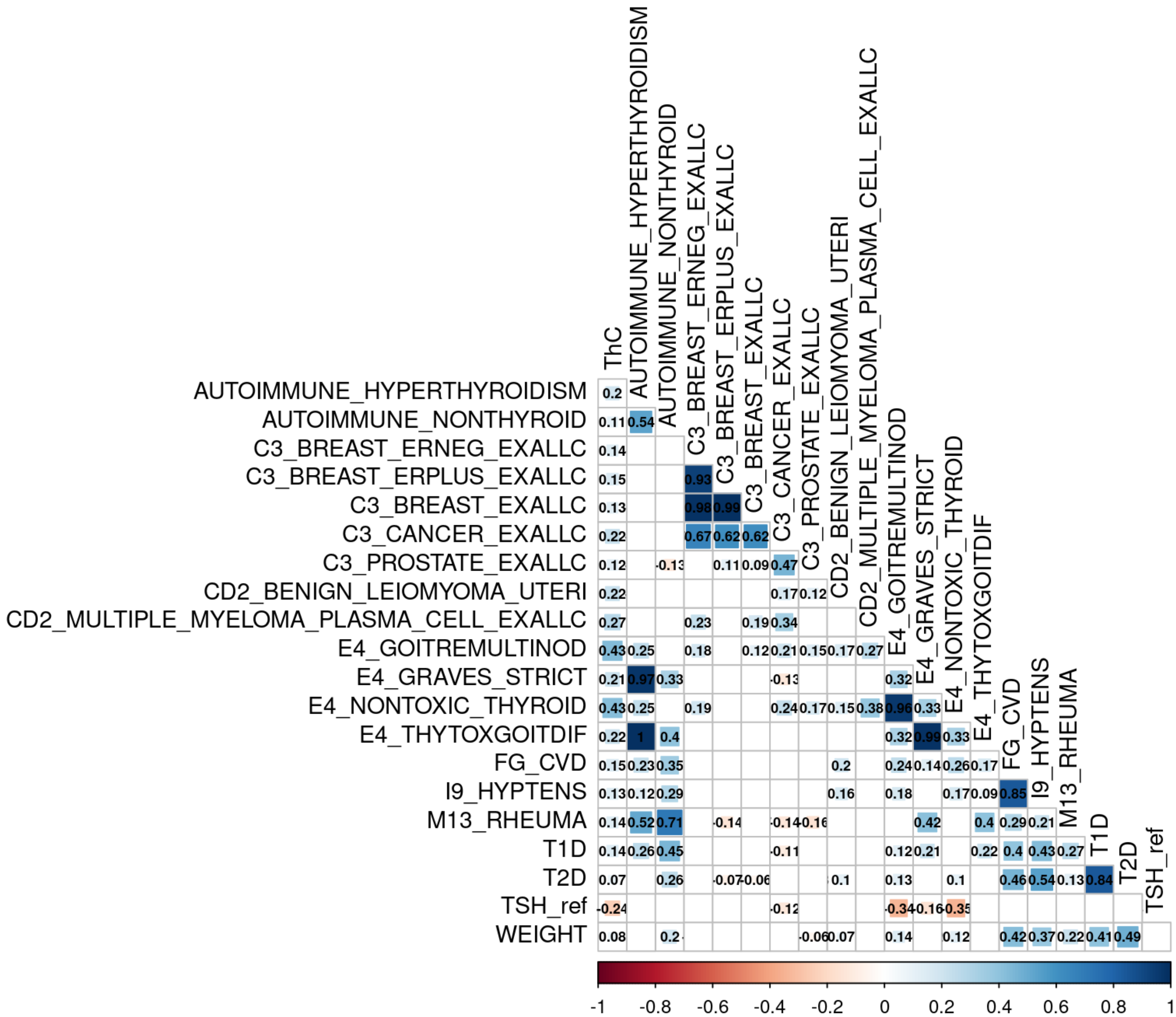

Supplementary Figure 4. Cluster weights and trait filtering results from Bayesian non-negative matrix (bNMF) factorization analysis. **a.** Cluster weights for thyroid cancer (ThC) loci (W matrix). Matrix W contains the locus-to-cluster weights derived from bNMF. Each row corresponds to a ThC susceptibility locus and each column to an inferred cluster. The weights represent the relative contribution of each locus to each cluster, with higher values indicating stronger assignment to a given mechanistic cluster. **b.** Cluster weights for traits (H matrix). Matrix H contains the trait-to-cluster weights. Each row corresponds to a trait included in the pleiotropy analysis and each column to a cluster. The weights quantify the extent to which each trait contributes to a specific cluster. Full cluster weights are shown in Supplementary Data 2, Table 5.

a

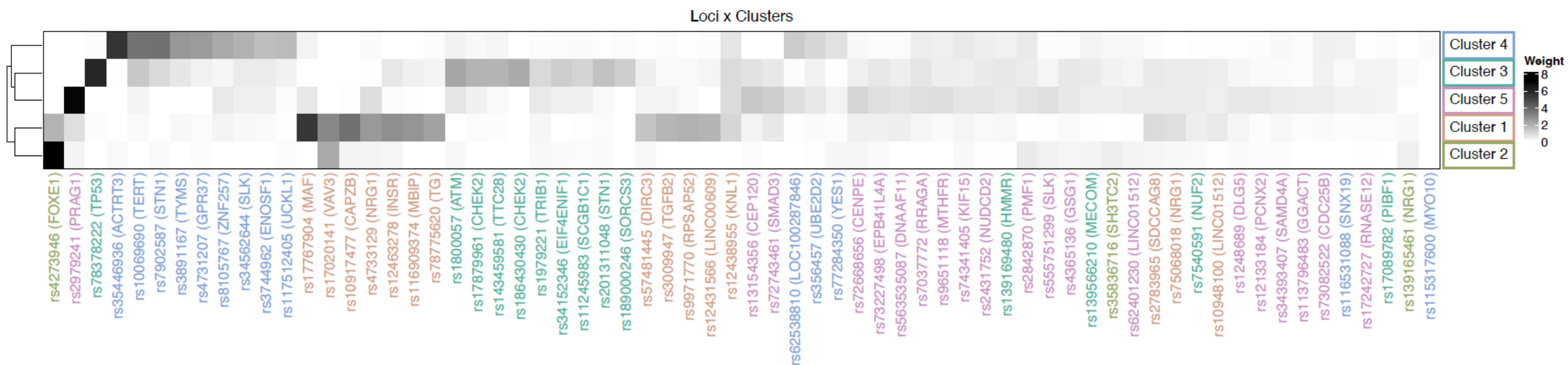

b

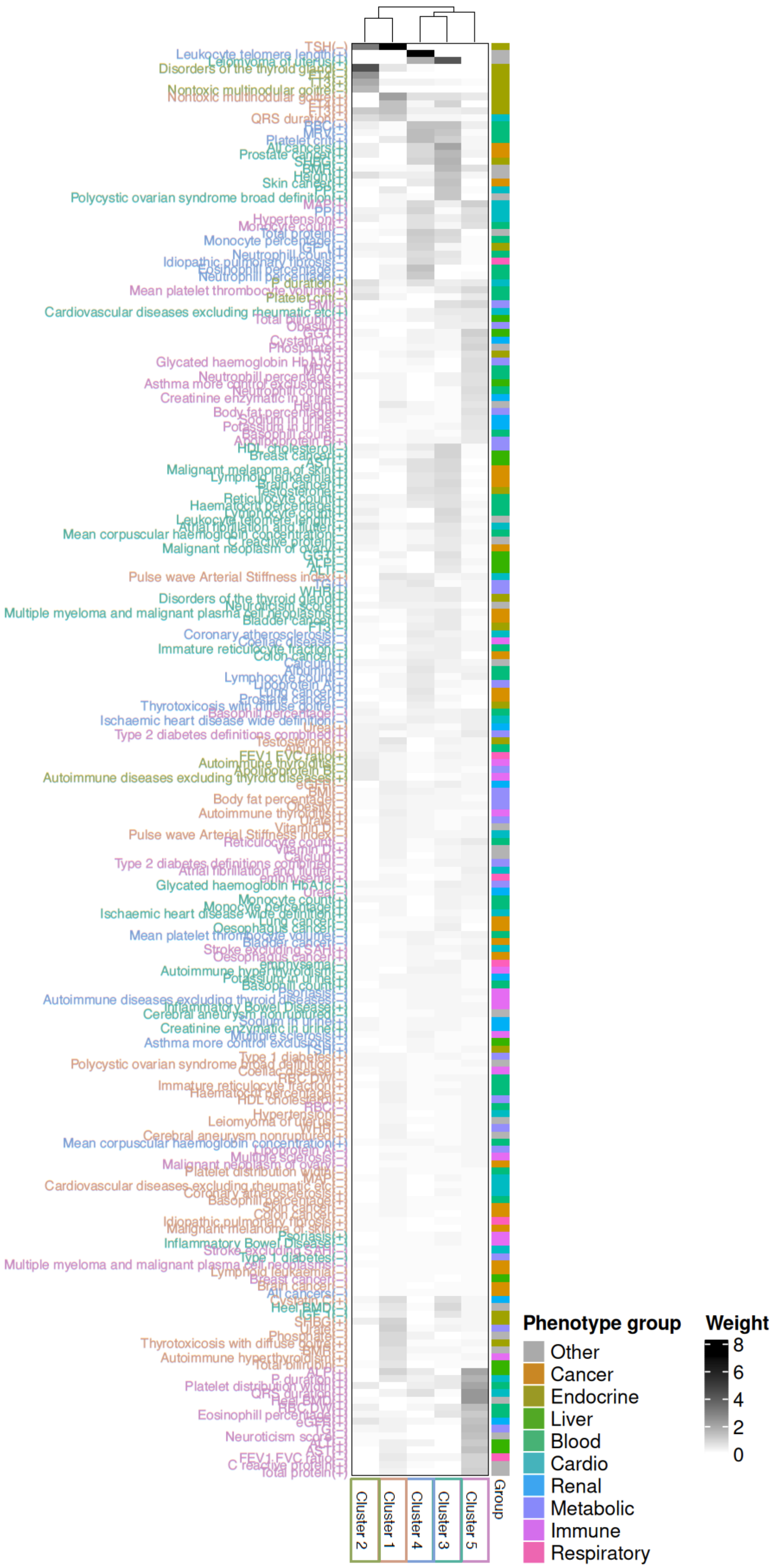
Cluster 2
Cluster 1
Cluster 4
Cluster 3
Cluster 5
Group
Phenotype group
Other
Cancer
Endocrine
Liver
Blood
Cardio
Renal
Metabolic
Immune
Respiratory
Weight
8
6
4
2
0

Supplementary Figure 5. Mendelian randomization analysis of TSH and thyroid cancer. Scatter plot shows the effect size of each variant on the circulating TSH levels derived from the ThyroidOmics consortium (Sterenborg et al., 2024) plotted against its effect size on the thyroid cancer meta-analysis performed in the present study. Causal estimates were obtained using inverse-variance weighted Mendelian randomization, with sensitivity analyses performed using MR-Egger regression, weighted median, and weighted mode methods. Error bars represent standard errors of SNP effect estimates. See corresponding effects in Supplementary Data 2, Table 19.

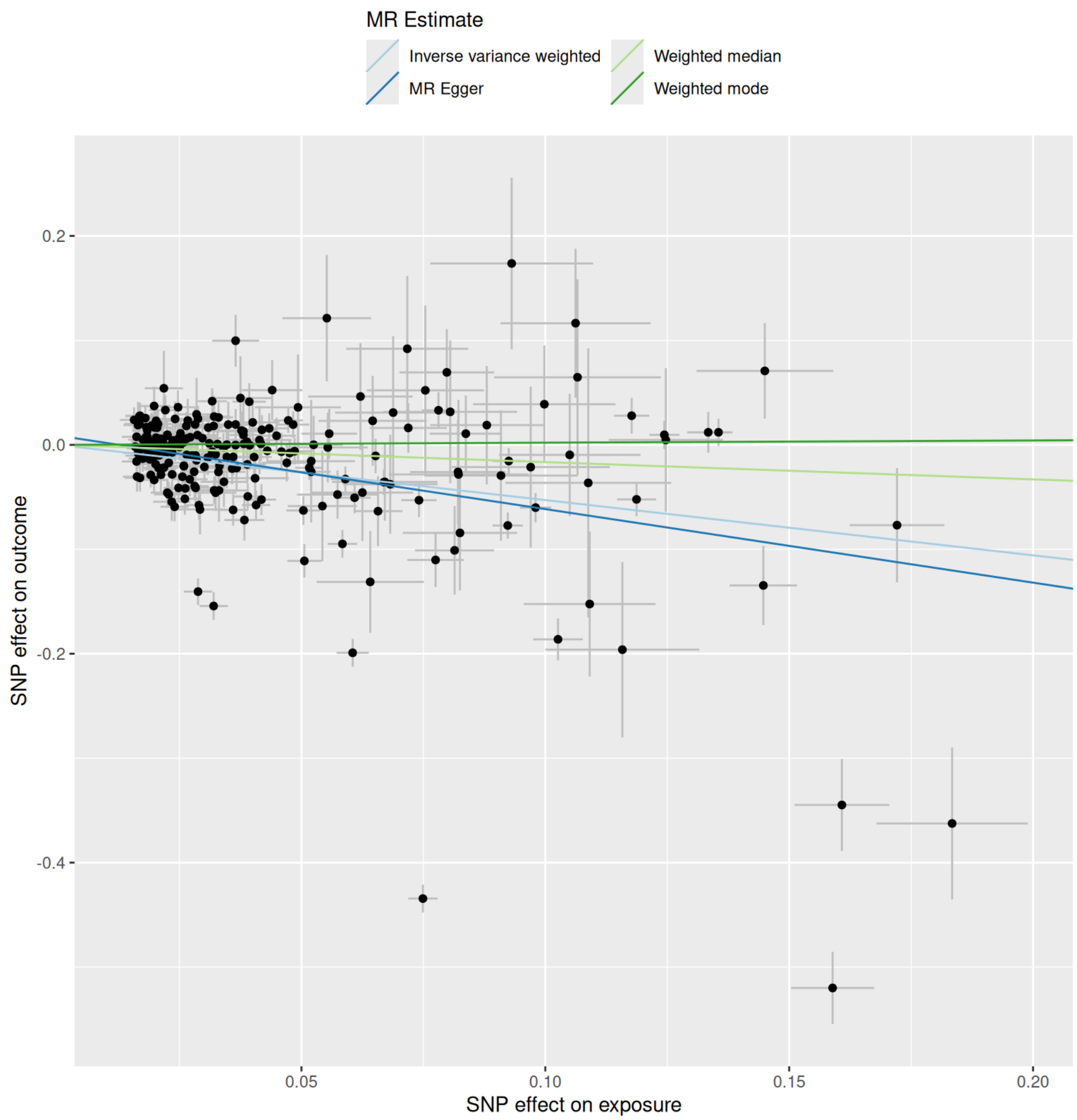

Supplementary Figure 6. Genetic overlap and two-Sample Mendelian randomization analyses between hyperthyroidism and thyroid cancer. **a.** Replication of hyperthyroidism lead SNPs in thyroid cancer GWAS. **b.** Mendelian randomization analysis between hyperthyroidism and thyroid cancer. Causal estimates were obtained using inverse-variance weighted Mendelian randomization, with sensitivity analyses performed using MR-Egger regression, weighted median, and weighted mode methods. Error bars represent standard errors of SNP effect estimates. See corresponding effects in Supplementary Data 2, Table 19. For hyperthyroidism (E4_THYTOXGOITDIF; primarily Graves disease), summary statistics were obtained from a FinnGen-MVP-UK Biobank meta-analysis(Kurki et al. 2023; Verma et al. 2024; Karczewski et al. 2025).

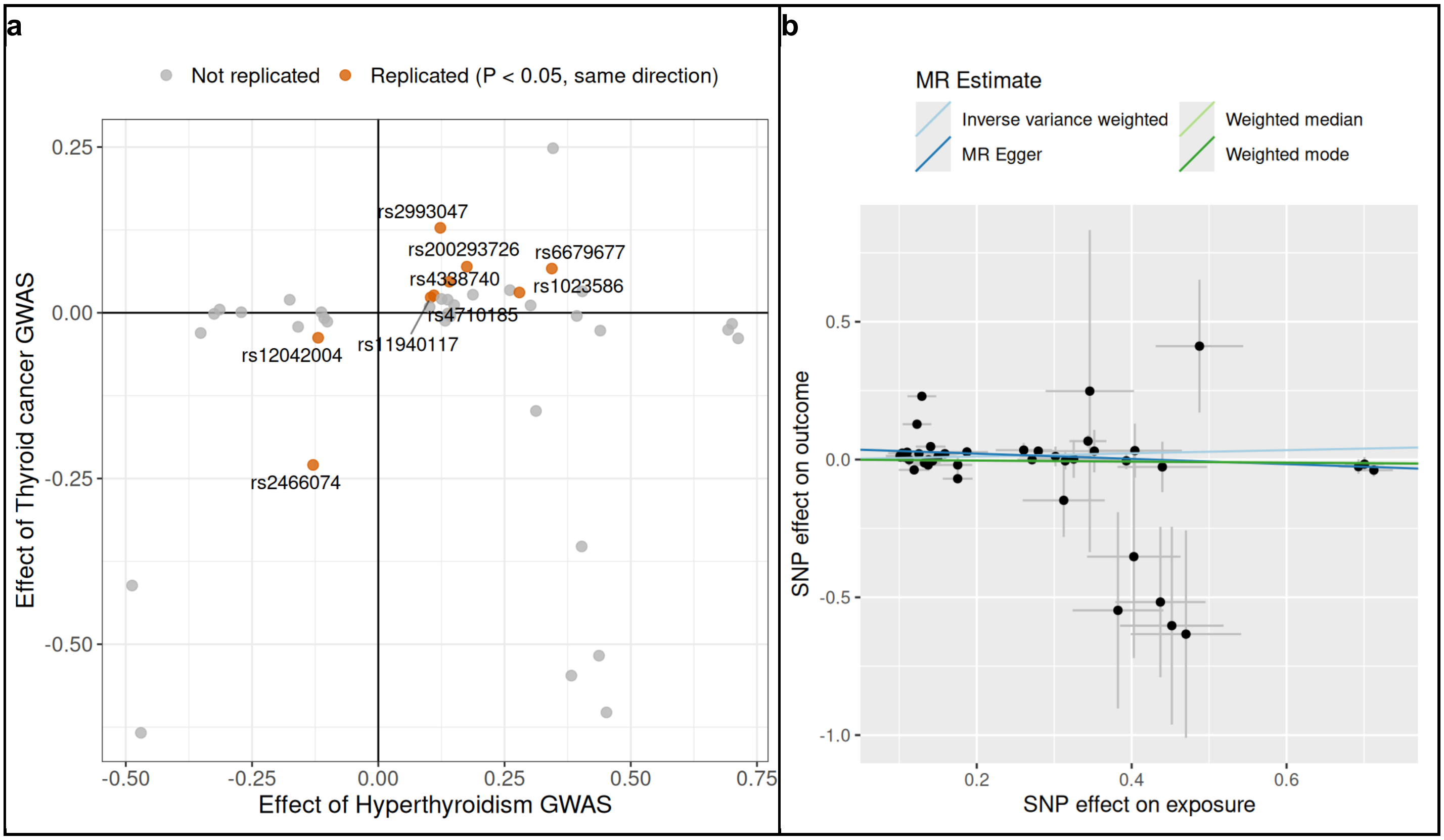

Supplementary Figure 7. Pathway enrichment of the top-weighted loci from each cluster. Left bars represent multiple-testing adjusted p-values of gene set analysis for pathways identified within each cluster. Colored squares represent locus involvement in the corresponding pathway. Statistical significance for pathway enrichment was assessed using cumulative hypergeometric tests implemented in g:Profiler (g:GOSt), with multiple-testing correction performed using the g:SCS method.

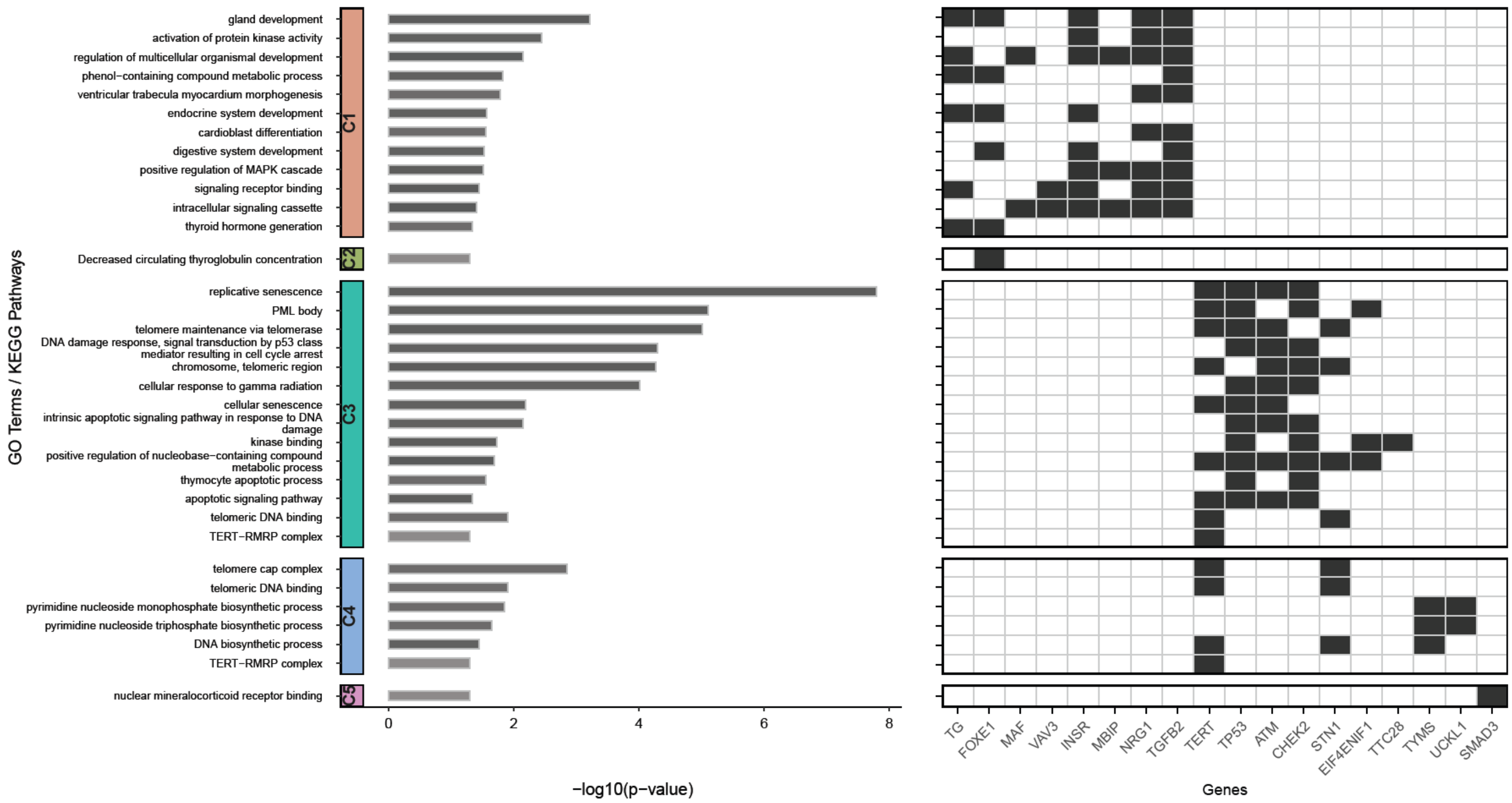

Supplementary Figure 8. A heatmap displaying the colocalization posterior probabilities (PP4) for each of the 53 unique independent thyroid cancer risk loci (rows) across 50 GTEx v10 tissues (columns). Each row is labeled by the locus's candidate gene, colored according to its assigned cluster. Color intensity corresponds to PP4 from the colocalization analysis. Full colocalization results are available in Supplementary Data 2, Table 13.

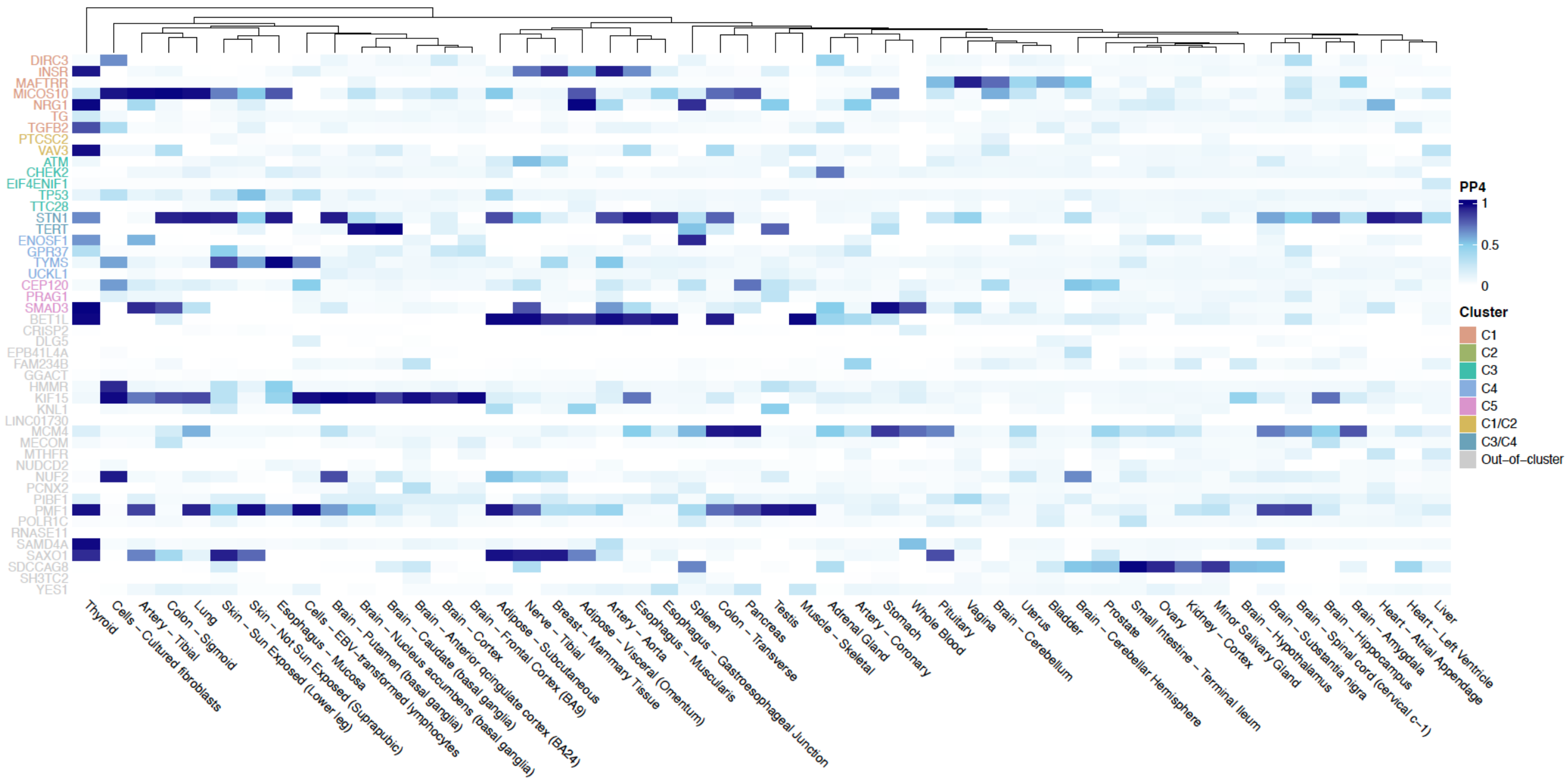

Supplementary Figure 9. Violin plots comparing the distribution of colocalization PP4 values in each tissue for loci belonging to a pleiotropic cluster (teal-blue, "In-Cluster") versus loci not assigned to any cluster (gray, "Out-of-Cluster"). Statistical significance was assessed using two-sided Wilcoxon rank-sum tests. For each of the GTEx v10 tissues analyzed, the violin plots display the distribution density, with center lines indicating medians and boxes indicating interquartile ranges. Full Wilcoxon test results are available in Supplementary Data 2, Table 15.

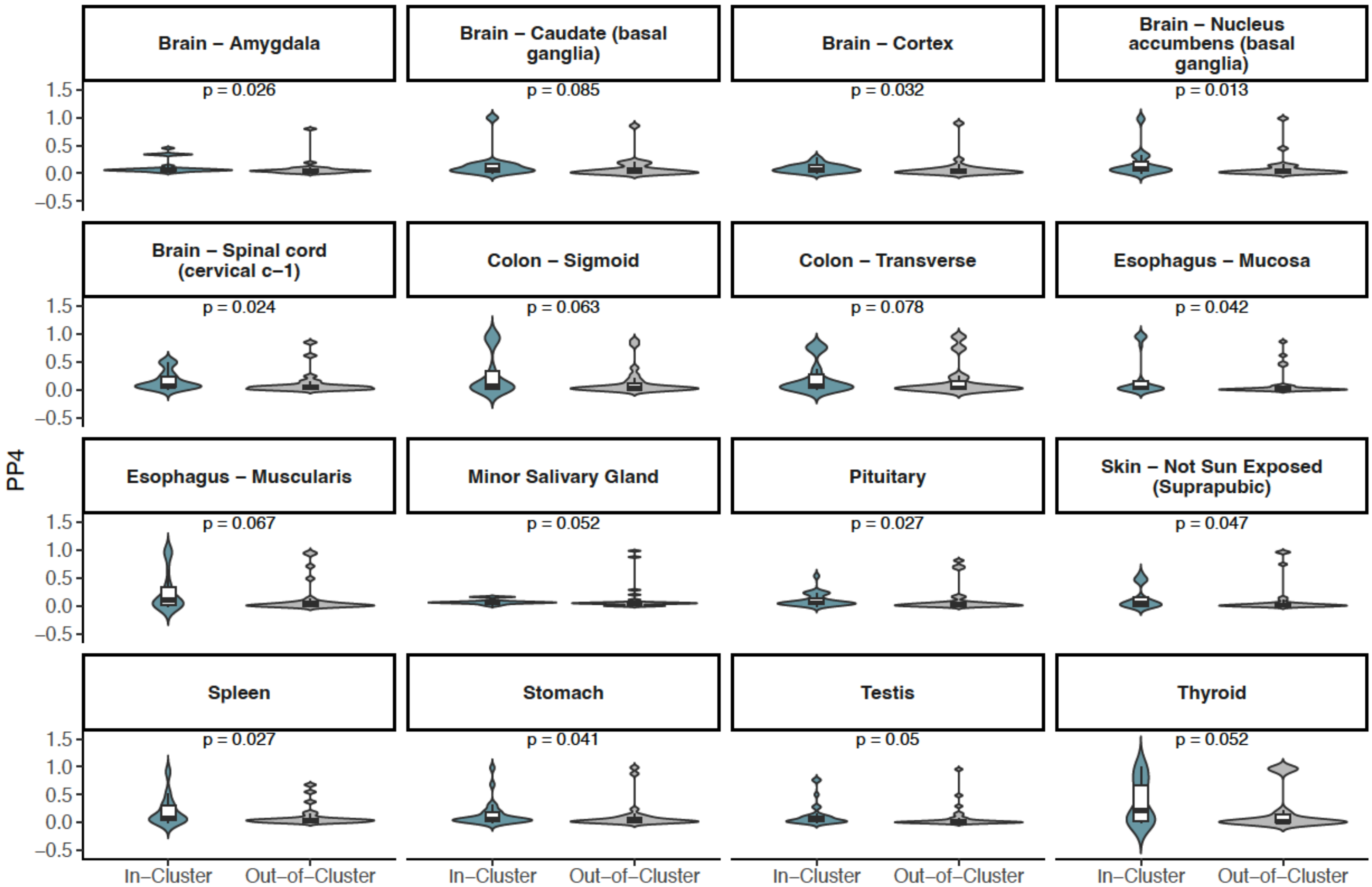

Supplementary Figure 10. Tissue-Specific Colocalization Counts by Cluster. Bar charts summarizing, for each pleiotropic cluster of thyroid cancer loci, how many genes colocalize with eQTLs in each tissue. Within a panel corresponding to GTEx v10 tissue, bars are grouped by cluster (C1-C5), with the colored portion of each bar indicating the number of genes in that cluster that have a colocalized eQTL in the given tissue (PP4 > 0.9), and the gray portion indicating the remaining cluster genes with no colocalization in that tissue. Numeric labels above the colored segments show the count of colocalized genes and, in parentheses, the percentage of the cluster's members that colocalize in that tissue. Full colocalization results are available in Supplementary Data 2, Table 13-14.

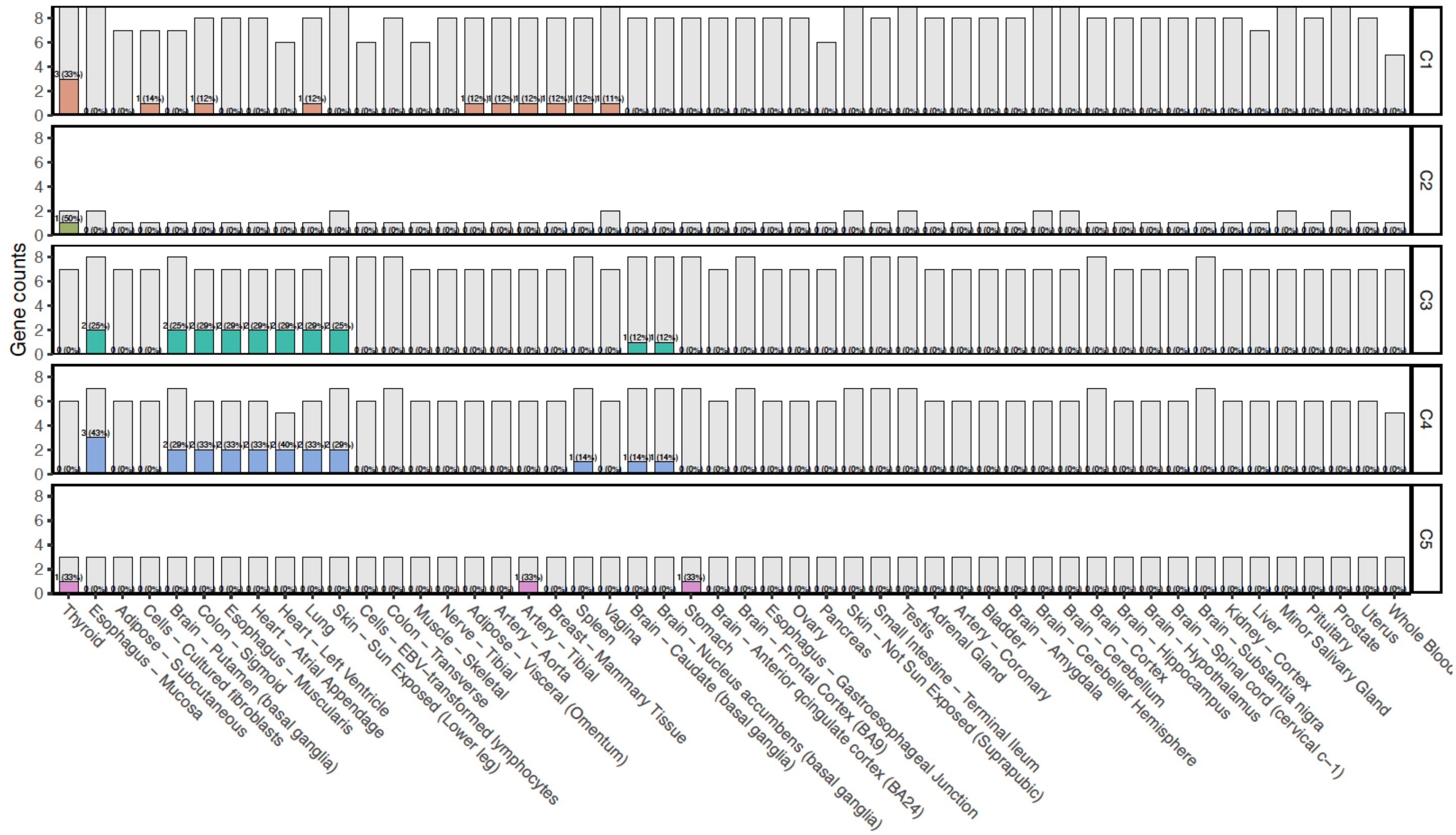

Supplementary Figure 11. Associations of cluster-specific partitioned polygenic score (pPGS) with thyroid cancer-related outcomes in AllofUs data. Summaries of the associations of each cluster-specific component of the pPGS with autoimmune disease, thyroid disease, cancer, and cardiometabolic traits. Points represent fixed-effects meta-analysis beta estimates per standard deviation increase in the pPGS across ancestry groups, and error bars represent 95% confidence intervals from two-sided association tests. Association testing was performed using logistic regression for binary traits and linear regression for continuous traits, adjusting for age, sex, and the first ten principal components within each ancestry group. Bonferroni correction was applied for the number of outcomes and clusters tested. Note: y-axis ranges differ across disease categories to reflect varying effect size distributions. See full results in Supplementary Data 2, Tables 8-9.

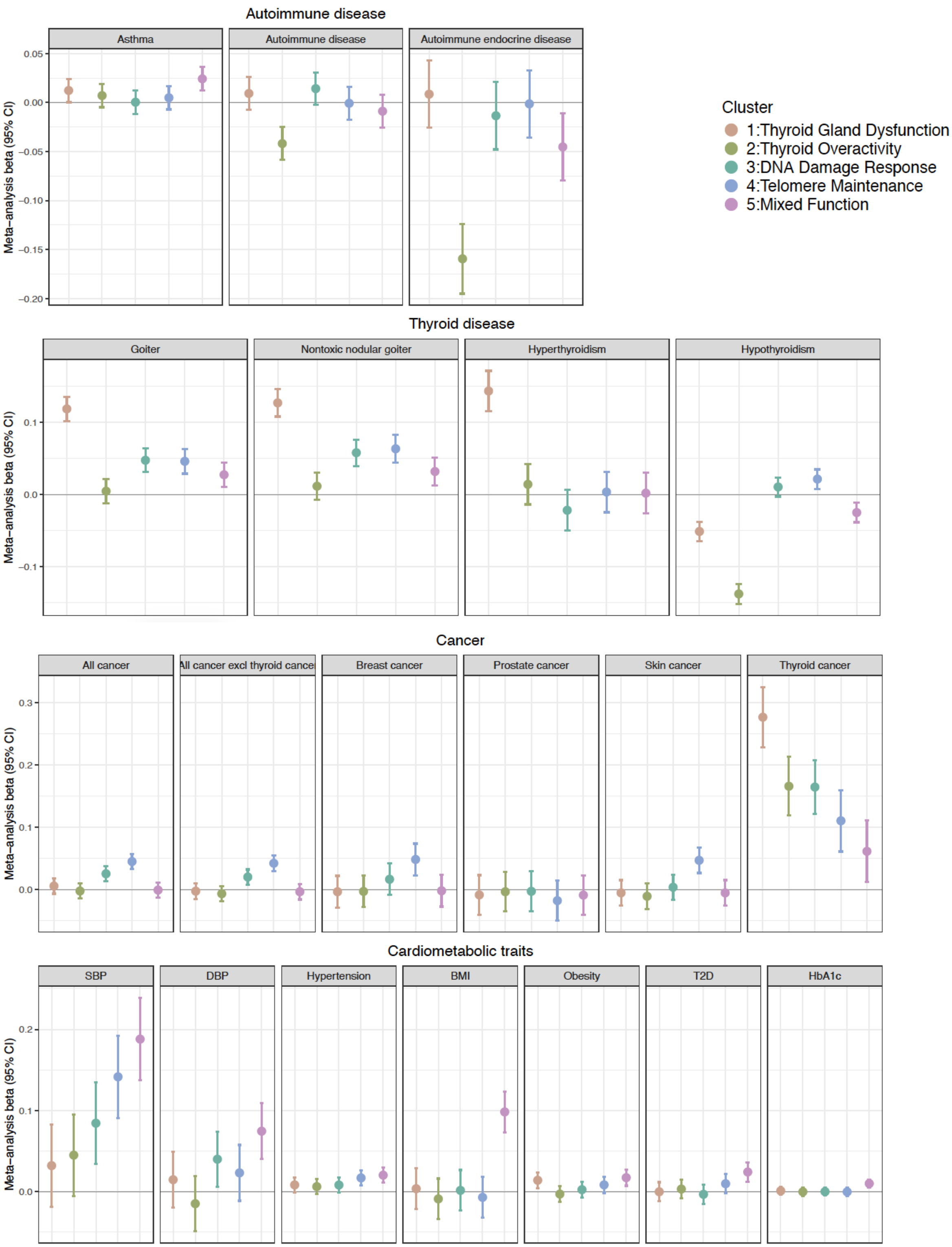

Supplementary Figure 12. Scatter plot comparing raw Z scores and scaled Z scores. Values from 66x105 matrix after removing 46 low p-value/correlated trait values. These two representations were highly correlated (Pearson r > 0.95). The clustering results from bNMF were robust to different normalization choices (Supplementary Data 2, Table 16).

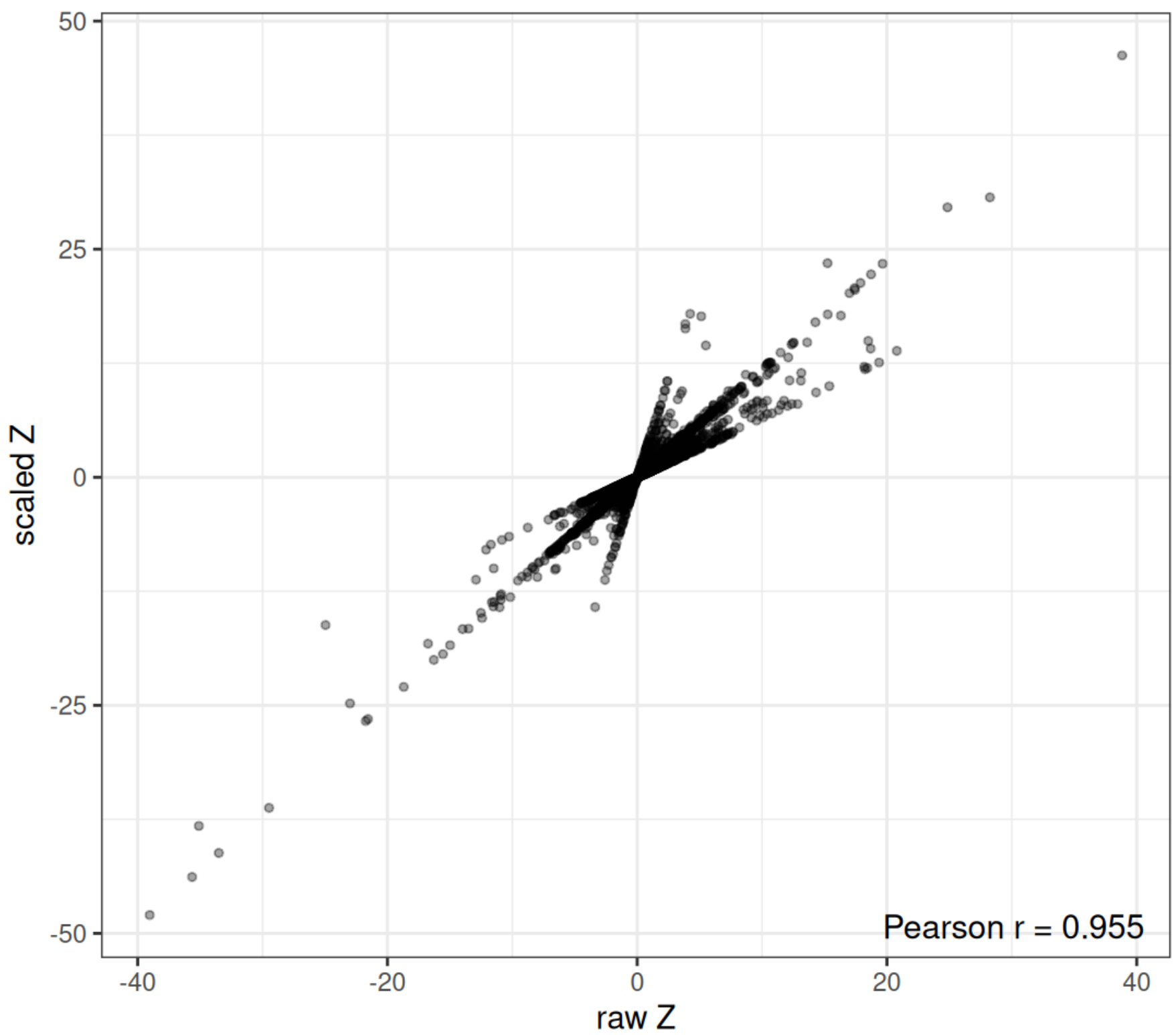

Supplementary Figure 13. Convergence evidence over iterations in bNMF

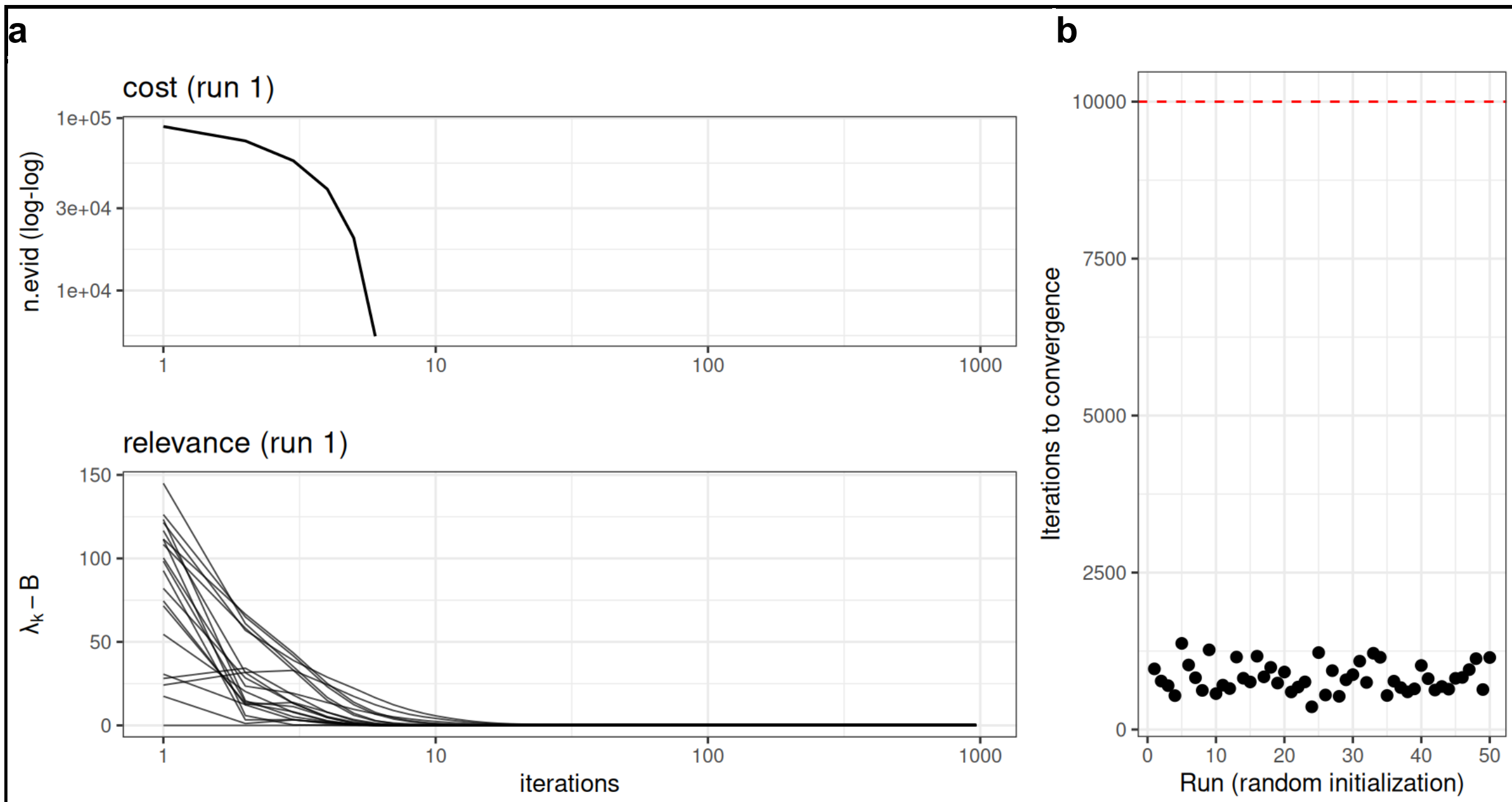

A) Top: evolution of the negative log-posterior (n.evid) across iterations (log–log scale). Bottom: trajectories of ARD relevance weights $\lambda_k - B$ across iterations (log-linear scale), where B denotes the baseline relevance level of pruned components. Both panels show convergence well before the maximum of 10,000 iterations.
B) Number of iterations required for convergence across 50 independent bNMF runs. All runs converged substantially before the maximum of 10,000 iterations (red dashed line), indicating that the iteration cap is conservative.
