# Supplementary material for: Genetic drivers of etiologic heterogeneity in thyroid cancer
Source: Nat Commun. 2026 Jul 10;17:8515. doi: 10.1038/s41467-026-75111-8 (PMC13483233; doi:10.1038/s41467-026-75111-8)
Supplement: Supplementary file 2 [file 41467_2026_75111_MOESM2_ESM.pdf]

Descriptions of Additional Supplementary Files

Supplementary Data 1: Supplementary methods describing summary statistic quality control, construction of the Z-score matrix, Bayesian nonnegative matrix factorization (bNMF) clustering, determination of the optimal number of clusters, and cluster-weight threshold selection.

Supplementary Data 2: Excel workbook containing Supplementary Tables 1-19, GWAS resources, enrichment analyses, Mendelian randomization results, and additional supporting datasets.
