# Supplementary material for: Genetic drivers of etiologic heterogeneity in thyroid cancer
Source: Nat Commun. 2026 Jul 10;17:8515. doi: 10.1038/s41467-026-75111-8 (PMC13483233; doi:10.1038/s41467-026-75111-8)
Supplement: Supplementary file 3 [file 41467_2026_75111_MOESM3_ESM.pdf]

Supplementary Information

Supplementary Data

Quality Control (QC)

GBMI Meta-Analysis & FinnGen_MVP_UKB Meta-Analysis

- Removed SNPs with inv_var_het_p < 0.05.
- Removed SNPs with leave_ukbb_inv_var_meta_het_p < 0.05.

Individual Biobank QC for FinnGen_MVP_UKB Meta-Analysis

Munging of FinnGen Summary Statistics

- Variants with imputation $r^2 < 0.6$ were discarded.

Munging of UKBB Summary Statistics

- Picard liftover was used to convert variants from GRCh37 to GRCh38 and align them to the reference genome.
- Variants with an allele frequency (AF) difference > 0.2 compared to the GnomAD NFE reference were discarded.

Munging of MVP Summary Statistics

- Variants with imputation $r^2 < 0.6$ were discarded.
- Variants with AF difference > 0.2 relative to the respective GnomAD ancestry reference were removed.
- Standard errors were recalculated from p-values and betas due to observed inflated standard errors for rare variants.

Individual Biobank QC: KCPS2, GHI, Latvian, PMBB

- GHI: Removed SNPs with average imputation quality (Rsq_ave) < 0.3.
- All biobanks:
    - Removed SNPs with minor allele frequency (MAF) < 0.01.
    - Removed SNPs with absolute Beta > 3.

bNMF pipeline

Construction of Z score matrix

To estimate standardized effect sizes for variant-trait associations, we calculated the ratio of the GWAS regression coefficient (beta) to its standard error using summary statistics. Given the variation in sample sizes across studies, we adjusted for these differences by scaling the standardized effect sizes by the square root of the study size, estimated as the mean sample size across all SNPs. This transformation resulted in the variant-trait association matrix $Z \in R^{N \times T}$ (66 variants × 151 traits).

Mathematically:

$$Z'_{ij} = \frac{Z_{ij}}{\sqrt{N_{ij}}} \times S, where\ S = \frac{1}{T}\sum_{j=1}^{T} \sqrt{median_i(N_{ij})}$$

- Z'_{ij} is the adjusted z-score for variant *i* and trait *j*.
- Z_{ij} is the original z-score.
- N_{ij} is the sample size for variant *i* and trait *j*.
- S is the scaling factor computed earlier

Each column of Z was further divided into two components: One capturing positive z-scores, and another capturing negative z-scores, which were multiplied by -1, with zeros assigned elsewhere. This modification produced the association matrix X (66 × 302), where traits were duplicated to account for both positive and negative associations with genetic variants. The bNMF algorithm then decomposed X into two matrices, W (66 × K) and H^T (302 × K), using an optimal rank K, following the approximation X ≈ WH. These matrices represent variant association patterns (W) and trait association patterns (H) for the identified clusters. This transformation allows bNMF to preserve both positive and negative associations, while ensuring the constraint of nonnegativity is maintained.

To assess robustness to the choice of standardization, we performed a sensitivity analysis comparing raw Z-scores and sample-size-adjusted Z-scores. The clustering results from bNMF were robust to different normalization choices (Supplementary Table 16, Supplementary Figure 11).

Determining the optimal Number of Clusters (K) in bNMF

A crucial aspect of bNMF is determining the appropriate number of clusters (**K**) while balancing data accuracy and model complexity. Traditional nonnegative matrix factorization (NMF) requires **K** to be predefined or determined after processing, whereas bNMF estimates the optimal **K** during analysis. This is achieved by balancing the reconstruction error ($\|\mathbf{X} - \mathbf{WH}\|^2$) with a complexity penalty, derived from a nonnegative half-normal prior applied to **W** and **H** (Tan and Févotte 2013; Kim et al. 2016; Kasar et al. 2015). Additionally, bNMF employs an automatic relevance determination method that iteratively eliminates less relevant components in **X**. More specifically, each column in **W** and each row in **H** is regulated by a shared relevance weight, which corresponds to the variance of the half-normal priors and is sampled from inverse-gamma hyperpriors.

The bNMF algorithm optimizes a posterior function that consists of two opposing terms: a likelihood function (Frobenius norm) and a regularization term (L2-norm of **W** and **H**, linked by relevance weights) (Tan and Févotte 2013). We used the Euclidean divergence, corresponding to a Gaussian noise model, which is appropriate for standardized GWAS association statistics. Alternative divergences (Kullback-Leibler or Itakura-Saito) assume count-based or multiplicative noise structures that are not consistent with the distributional properties of these data.

Through iterative refinement, bNMF suppresses uninformative components by reducing relevance weights, enabling optimal inference of the number of clusters (**K**) while ensuring a sparse representation of **W** and **H**. This process maintains a balance between capturing meaningful data structure (Frobenius norm) and avoiding excessive model complexity (regularization penalty). Each cluster is characterized by its most strongly associated traits, which naturally emerge from the bNMF analysis.

The bNMF algorithm was implemented in RStudio and executed for 10,000 iterations with varying initial conditions. In each bNMF run, the factor matrices W and H were initialized with nonnegative values from independently and identically distributed according to a uniform (0,1) distribution and scaled by the maximum value of the input matrix, following the original automatic relevance determination NMF formulation (Tan and Févotte, 2013) and prior applications in genetic clustering (Udler et al., 2018). The distribution of converged K values is shown below. The most probable **K** (determined as the most frequently observed **K** across 50 iterations) was selected for further analysis. After selection of the optimal **K**, bNMF was re-run using this fixed value to obtain the final factorization. The final clustering results provided cluster-specific weights for each variant (**W**) and trait (**H**).

Determining the optimal K from the bNMF run summary

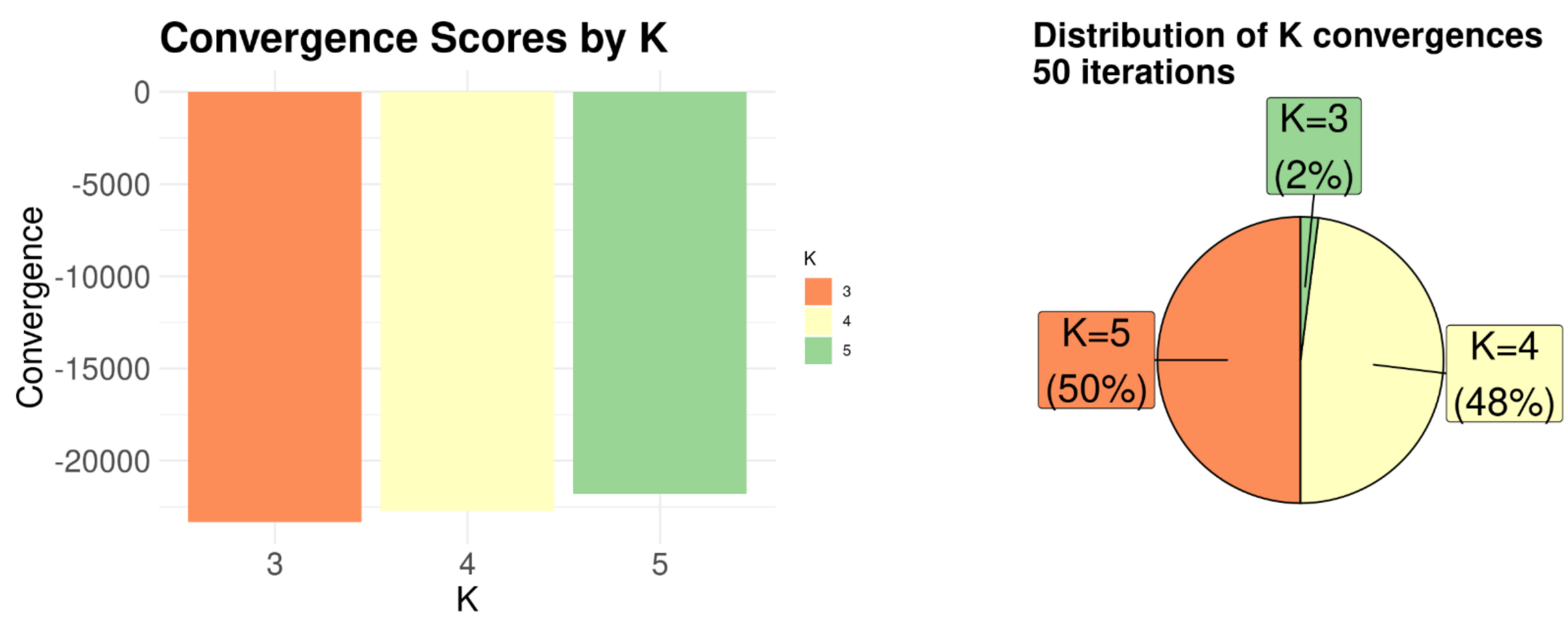

Determining the Optimal Cutoff for Clustering Weights

To establish the threshold separating informative from less informative variants, we followed a long-tail analysis of clustering weights, as previously described (Udler et al. 2018). We assessed the distribution of variant clustering weights across all clusters. First, we ranked clustering weight deltas in descending order and defined the "long tail" as beginning after the last significant change in clustering weight deltas. Top 5% of clustering weight deltas were considered significant, marking the threshold for variant inclusion within each cluster.

The last significant delta corresponded to a clustering weight of 1.057017, indicated by the red dot in the figure below.

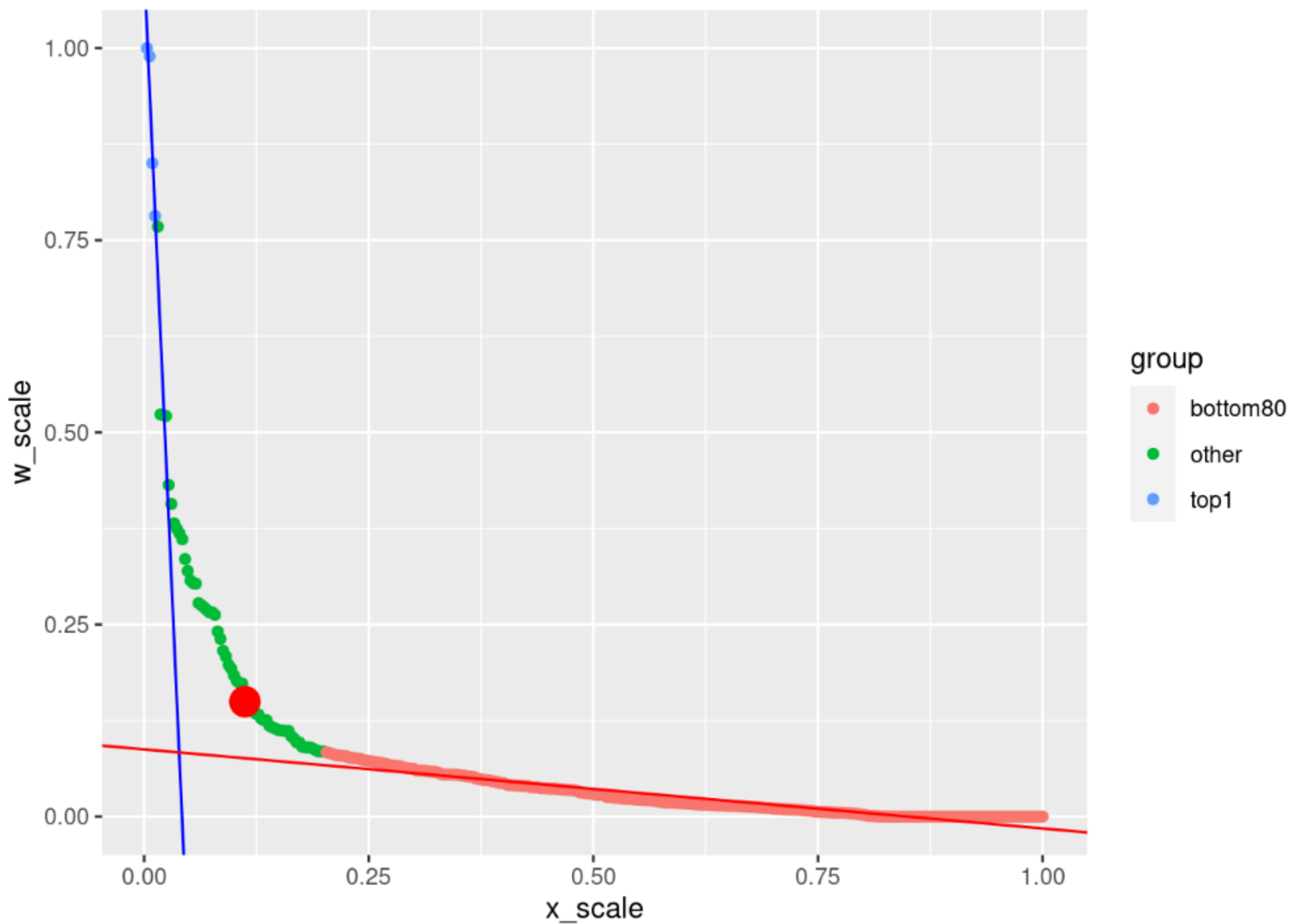

References

Kasar, S., J. Kim, R. Improgo, G. Tiao, P. Polak, N. Haradhvala, M. S. Lawrence, et al. 2015. “Whole-Genome Sequencing Reveals Activation-Induced Cytidine Deaminase Signatures during Indolent Chronic Lymphocytic Leukaemia Evolution.” *Nature Communications* 6 (1): 8866.

Kim, Jaegil, Kent W. Mouw, Paz Polak, Lior Z. Braunstein, Atanas Kamburov, David J. Kwiatkowski, Jonathan E. Rosenberg, Eliezer M. Van Allen, Alan D’Andrea, and Gad Getz. 2016. “Somatic ERCC2 Mutations Are Associated with a Distinct Genomic Signature in Urothelial Tumors.” *Nature Genetics* 48 (6): 600–606.

Tan, Vincent Y. F., and Cédric Févotte. 2013. “Automatic Relevance Determination in Nonnegative Matrix Factorization with the β-Divergence.” *IEEE Transactions on Pattern Analysis and Machine Intelligence* 35 (7): 1592–1605.

Udler, Miriam S., Jaegil Kim, Marcin von Grotthuss, Sílvia Bonàs-Guarch, Joanne B. Cole, Joshua Chiou, Christopher D. Anderson on behalf of METASTROKE and the ISGC, et al. 2018. “Type 2 Diabetes Genetic Loci Informed by Multi-Trait Associations Point to Disease Mechanisms and Subtypes: A Soft Clustering Analysis.” *PLoS Medicine* 15 (9): e1002654.
