# Supplementary material for: Genetic drivers of etiologic heterogeneity in thyroid cancer
Source: Nat Commun. 2026 Jul 10;17:8515. doi: 10.1038/s41467-026-75111-8 (PMC13483233; doi:10.1038/s41467-026-75111-8)
Supplement: Supplementary file 4 [file 41467_2026_75111_MOESM5_ESM.pdf]

nature portfolio

Corresponding author(s): Alicia Martin

Last updated by author(s): May 26, 2026

# Reporting Summary

Nature Portfolio wishes to improve the reproducibility of the work that we publish. This form provides structure for consistency and transparency in reporting. For further information on Nature Portfolio policies, see our Editorial Policies and the Editorial Policy Checklist.

## Statistics

For all statistical analyses, confirm that the following items are present in the figure legend, table legend, main text, or Methods section.

| n/a | Confirmed | |
|---|---|---|
| ☐ | ☒ | The exact sample size (*n*) for each experimental group/condition, given as a discrete number and unit of measurement |
| ☐ | ☒ | A statement on whether measurements were taken from distinct samples or whether the same sample was measured repeatedly |
| ☐ | ☒ | The statistical test(s) used AND whether they are one- or two-sided<br>*Only common tests should be described solely by name; describe more complex techniques in the Methods section.* |
| ☐ | ☒ | A description of all covariates tested |
| ☐ | ☒ | A description of any assumptions or corrections, such as tests of normality and adjustment for multiple comparisons |
| ☐ | ☒ | A full description of the statistical parameters including central tendency (e.g. means) or other basic estimates (e.g. regression coefficient) AND variation (e.g. standard deviation) or associated estimates of uncertainty (e.g. confidence intervals) |
| ☐ | ☒ | For null hypothesis testing, the test statistic (e.g. *F*, *t*, *r*) with confidence intervals, effect sizes, degrees of freedom and *P* value noted<br>*Give P values as exact values whenever suitable.* |
| ☐ | ☒ | For Bayesian analysis, information on the choice of priors and Markov chain Monte Carlo settings |
| ☐ | ☒ | For hierarchical and complex designs, identification of the appropriate level for tests and full reporting of outcomes |
| ☐ | ☒ | Estimates of effect sizes (e.g. Cohen's *d*, Pearson's *r*), indicating how they were calculated |

*Our web collection on statistics for biologists contains articles on many of the points above.*

## Software and code

Policy information about availability of computer code

| | |
|---|---|
| Data collection | No software was used in data collection. |
| Data analysis | The code used for meta-analysis and pleiotropy dissection has been deposited in Zenodo at https://doi.org/10.5281/zenodo.20433102 and is maintained at https://github.com/yonhojee/thyroid_cancer_bnmf (v1.0.0). Analyses were conducted using SAIGE (v0.29.5), METAL (v2020-05-05); autoreporting, https://github.com/FINNGEN/autoreporting (v2020-10-26); LDSC (v2015-02-12); bNMF clustering, https://github.com/gwas-partitioning/bnmf-clustering (v1); g:Profiler; coloc (v6); TwoSampleMR (v0.7); MR-PRESSO; R; and RStudio. |

For manuscripts utilizing custom algorithms or software that are central to the research but not yet described in published literature, software must be made available to editors and reviewers. We strongly encourage code deposition in a community repository (e.g. GitHub). See the Nature Portfolio guidelines for submitting code & software for further information.

## Data

Policy information about availability of data

All manuscripts must include a data availability statement. This statement should provide the following information, where applicable:

- Accession codes, unique identifiers, or web links for publicly available datasets
- A description of any restrictions on data availability
- For clinical datasets or third party data, please ensure that the statement adheres to our policy

The sources for GWAS summary statistics are provided in Supplementary Tables 1 and 4. The meta-analysis GWAS summary statistics are publicly available at

https://doi.org/10.5281/zenodo.18932445. For the meta-analysis of thyroid cancer, we combined GWAS summary statistics from two meta-analyses (the GBMI meta-analysis and a separate meta-analysis of FinnGen R12 and MVP) and four individual biobanks (the Korean Cancer Prevention Study-II Biobank, Latvian National Biobank, Penn Medicine BioBank, and Genomic Health Initiative). The GBMI (https://www.globalbiobankmeta.org/resources) and FinnGen-UKB-MVP meta-analysis data (leave-UKB-out) (https://mvp-ukbb.finngen.fi/) are publicly available, whereas summary statistics for the individual biobanks are available upon request. All data sources used for the clustering analysis are listed in Supplementary Table 4.

## Research involving human participants, their data, or biological material

Policy information about studies with human participants or human data. See also policy information about sex, gender (identity/presentation), and sexual orientation and race, ethnicity and racism.

| | |
|---|---|
| Reporting on sex and gender | Self-reported sex was used as a covariate in our analysis. |
| Reporting on race, ethnicity, or other socially relevant groupings | Individuals were grouped by genetic ancestry. We conducted multi-ancestry meta-analysis, combining GWAS summary statistics from two meta-analyses (the GBMI meta-analysis and a separate meta-analysis of FinnGen R12 and MVP) and four individual biobanks (the Korean Cancer Prevention Study-II Biobank, Latvian National Biobank, Penn Medicine BioBank, and Genomic Health Initiative) (Supplementary Table 1). |
| Population characteristics | We did not perform new recruitment in this study and used existing cohorts and datasets. The description of the dataset for each GWAS can be found in the original publications as listed in Supplementary Table 1 and 4.<br>Individual-level GWAS was performed using Korean Cancer Prevention Study-II Biobank (KCPS2), adjusting for age, sex, and the first ten genetic principal components in SAIGE. KCPS2 is a prospective cohort study based in Korea with genotype data and measurements of a wide range of phenotypes collected from 153,950 subjects. Mean age of participants at recruitment was 41.7 yr old, and 40% were female. All participants in the KCPS2 were genotyped using either the Illumina Global Screening Array (GSA) v2.0 (78,260 samples) or the Korean Chip array v1 (90,245 samples). For the other contributing biobanks included in the thyroid cancer GWAS meta-analysis, we used publicly available GWAS summary statistics or summary statistics generated by the corresponding studies. Description of the dataset for GWAS from other biobanks can be found in the original publications as listed in Supplementary Table 14.<br>We derived and validated partitioned polygenic scores using data from the participants All of Us Research Program (AoU). Briefly, AoU is longitudinal cohort study continuously enrolling adults in the US with genotype, self-reported, and linked health record data. The program places a strong emphasis on including diverse populations that have traditionally been underrepresented in biomedical research. |
| Recruitment | We did not recruit participants for this study; we used existing GWAS summary statistics and individual-level biobank data from the KCPS2 and the All of Us Research Program. |
| Ethics oversight | All KCPS2 participants provided written informed consent approved from ethics committees of Yonsei University. For the All of Us Research Program, informed consents for all the participants were conducted in person or through an eConsent platform. The protocol was reviewed by the Institutional Review Board (IRB) of the All of Us Research Program. Data were accessed through the All of Us Research Workbench, a secure cloud-based analytic platform. |

Note that full information on the approval of the study protocol must also be provided in the manuscript.

# Field-specific reporting

Please select the one below that is the best fit for your research. If you are not sure, read the appropriate sections before making your selection.

☒ Life sciences ☐ Behavioural & social sciences ☐ Ecological, evolutionary & environmental sciences

For a reference copy of the document with all sections, see nature.com/documents/nr-reporting-summary-flat.pdf

# Life sciences study design

All studies must disclose on these points even when the disclosure is negative.

| | |
|---|---|
| Sample size | Our meta-analysis of thyroid cancer in resulted in a total 16,167 cases and 2,430,374 controls. The clustering of thyroid was based on GWAS summary results for the independent thyroid-cancer index variants across 151 phenotypes (the sample sizes for each of these phenotypes are summarized in Supplementary Table 4). We prioritized multi-ancestry GWAS for ancillary trait summary statistics; however, when unavailable for a given trait, European-based GWASs were used. |
| Data exclusions | Samples were selected based on quality control criteria specific to each cohort, as detailed in Supplementary Data. |
| Replication | We evaluated the performance of our partitioned polygenic scores in a held-out samples of AoU. |
| Randomization | Randomization was not applicable in this study. |
| Blinding | Blinding was not employed because the study involved no interventions and focused solely on genotype-phenotype associations. |

# Reporting for specific materials, systems and methods

We require information from authors about some types of materials, experimental systems and methods used in many studies. Here, indicate whether each material, system or method listed is relevant to your study. If you are not sure if a list item applies to your research, read the appropriate section before selecting a response.

## Materials & experimental systems

| n/a | Involved in the study |
| --- | --- |
| ☒ | ☐ Antibodies |
| ☒ | ☐ Eukaryotic cell lines |
| ☒ | ☐ Palaeontology and archaeology |
| ☒ | ☐ Animals and other organisms |
| ☒ | ☐ Clinical data |
| ☒ | ☐ Dual use research of concern |
| ☒ | ☐ Plants |

## Methods

| n/a | Involved in the study |
| --- | --- |
| ☒ | ☐ ChIP-seq |
| ☒ | ☐ Flow cytometry |
| ☒ | ☐ MRI-based neuroimaging |

# Plants

| | |
| --- | --- |
| Seed stocks | *Report on the source of all seed stocks or other plant material used. If applicable, state the seed stock centre and catalogue number. If plant specimens were collected from the field, describe the collection location, date and sampling procedures.* |
| Novel plant genotypes | *Describe the methods by which all novel plant genotypes were produced. This includes those generated by transgenic approaches, gene editing, chemical/radiation-based mutagenesis and hybridization. For transgenic lines, describe the transformation method, the number of independent lines analyzed and the generation upon which experiments were performed. For gene-edited lines, describe the editor used, the endogenous sequence targeted for editing, the targeting guide RNA sequence (if applicable) and how the editor was applied.* |
| Authentication | *Describe any authentication procedures for each seed stock used or novel genotype generated. Describe any experiments used to assess the effect of a mutation and, where applicable, how potential secondary effects (e.g. second site T-DNA insertions, mosiacism, off-target gene editing) were examined.* |
