# Supplementary material for: Genetic drivers of etiologic heterogeneity in thyroid cancer
Source: Nat Commun. 2026 Jul 10;17:8515. doi: 10.1038/s41467-026-75111-8 (PMC13483233; doi:10.1038/s41467-026-75111-8)
Supplement: Supplementary file 5 [file 41467_2026_75111_MOESM6_ESM.pdf]

nature portfolio

Peer Review File

Genetic drivers of etiologic heterogeneity in thyroid cancer

Corresponding Author: Dr Alicia Martin

This file contains all reviewer reports in order by version, followed by all author rebuttals in order by version.

Version 0:

Reviewer comments:

Reviewer #1

(Remarks to the Author)
Jee et al. performed a large GWAS to analyze the genetic drivers of multiple forms of thyroid cancer. Particularly, they analyzed whether there are various genetic clusters. I have a number of concerns:

1.From previous thyroid cancer GWAS results it has already been clear that part of the variants are known thyroid hormone regulation genes, while others are more involved in general oncogenic pathways. The fact that formal clustering strategies identify these clusters is therefore not surprising at all. Furthermore, one wonders what the follow up analyses really add to our knowledge: The fact that the thyroid hormone rgeulation cluster is associated with thyroid function or other thyroid diseases is neither suprising (actually it is known that these variants associate with many thyroid related endpoints - see previous GWAS on thryoid (dys)function). The authors for example identify an association with thyroid autonomy suggesting that thyroid autonomy may play a role in developing thyroid cancer. To gain more insight into this statement, the authors should perform much more analyses to bolster this statement, for example testing whether other thyroid autonomy related variants do or do not associate with thyroid cancer, and if so, why. The same holds true for hyperthyroidism (what is the role of Graves variants next to thyroid autonomy variants).

2. The authors suggest that these pathways play a key role in thyroid cancer pathogenesis. What is the explained variance?

Minor: The authors state that they substantially increased sample size compared to previous GWAS by analyzing 2.5 M subjects. However the far majority are controls (2.4 M).

Reviewer #2

(Remarks to the Author)
The authors of “Genetic drivers of etiologic heterogeneity in thyroid cancer” propose a new analysis of a large cohort of patients. They propose using non-negative matrix factorization to identify clusters associated with specific genetic processes and construct polygenic scores to try to better understand the genetic architecture of thyroid cancer.

Overall, I find the paper well written and well structured. The analysis is supported by functional validation with tissue enrichment, pathway analysis, and replication of cluster-specific polygenic scores in an external cohort.

Main comments:

- Distributional assumptions: Ref. 115 provides several NMF estimation algorithms with automatic relevance determination of the rank K with the Itakura-Saito, Kullback-Leibler or Euclidean divergences. The authors used the Frobenius norm in this manuscript; however, this choice is not motivated. A discussion of which of these models is suitable for the data analyzed in this manuscript is lacking here. The underlying model is crucial when applying NMF as incorrect distributional assumptions can lead to the wrong number of clusters and not well-defined features.

- Convergence:
1) the initial conditions are another important factor for NMF. The manuscript states that the algorithm is executed for 10,000

iterations with varying initial conditions. How were the initial conditions chosen? More details would be helpful here and would also help with the reproducibility of the analysis.
2) 10,000 iterations are usually sufficient for convergence in most NMF applications. A supplementary figure showing that this is also true in this particular case would be appreciated.

- Visualization and interpretation of clusters:
1) NMF can often result in clusters that are difficult to interpret mainly because they share several of their key top-weighted components. Table 1 reports on the top weighted traits and loci for each cluster, it would be very helpful to have a visualization of such clusters to provide more insights into the separability of the observed clusters (e.g. by showing a PCA on the results of non-negative matrix factorization).
2) Reporting the number of variants per cluster before and after applying the cutoff for clustering weights would help readers in judging sparsity and interpretability of the clusters.

- Standardization: it is unclear whether results are robust to different normalizations of z-scores and why this procedure has been selected. Is this a standard procedure in the literature? In this case, I would suggest adding some references. Would it be possible to use standard variance-weighted Z-scores from GWAS?

- Removal of highly correlated traits (lines 569-571): the reason for this step is not clear to me. Please motivate this further.

- Procedure for handling missing data (lines 579-580): are any of the results from Supplementary Table 11 contradicting expectations or showing new interesting traits? I do not have the expertise to judge this, but a comment on whether or not imputation provided new insights on the clusters would also add value to this step of the analysis.

Minor comments:

- Despite being a heuristic approach, I believe the cutoff for clustering weights determination can be an efficient solution for interpretability of the clusters. My understanding is that this is defined with an “elbow”-like approach. Cross-validation could be a more rigorous alternative for this, it might be interesting to study whether this would provide a different threshold.

- In my view, Figure 3 and 4 are very dense. Figure 3 might benefit from either a re-organization or a clearer connection between panels a-c and panel d. Figure 4 could be moved to supplements and could be replaced with a schematic summary of the current figure with main takeaways.

- Notation: in the NMF literature “features” is usually referring to what is termed as “clusters” in this manuscript. I would suggest including a comment on this as it can be confusing at first glance. References to older works on NMF such as Lee and Seung 2004 can also be included in this context.

- The mathematical formulation of the standardized z-score in the supplements is quite blurry. Could be written in-text instead of adding an image.

Reviewer #3

(Remarks to the Author)
This manuscript presents a multi-ancestry genome-wide investigation of thyroid cancer susceptibility, combining data from 18 studies. The authors identified 66 independent signals across 51 loci and integrated cross-trait evidence from 151 immune, metabolic, endocrine, and hematopoietic phenotypes. Using Bayesian non-negative matrix factorization, they categorized the signals into five putative mechanistic clusters and developed cluster-specific polygenic scores, which were externally validated in the All of Us cohort. Complementary functional analyses, including tissue/cell-type enrichment, eQTL colocalization, and genomic annotation, implicated pathways related to telomere maintenance, DNA damage response, thyroid biology, and autoimmunity. Overall, while the study leverages impressive datasets and a broad analytical scope, it would benefit from stronger methodological justification, sensitivity analyses, and clearer integration of functional evidence to enhance both novelty and robustness.

Major Revisions:
1. Independence of signals was defined using a Finnish LD reference panel and a threshold T, with additional steps applied when $T < 0.02$—an unconventional approach. Please justify the choice of reference panel, include sensitivity analyses using cosmopolitan or ancestry-specific LD panels, and compare results with standard clumping or conditional analysis methods.
2. The use of fixed-effect meta-analysis may not be appropriate in the presence of cross-cohort or cross-ancestry heterogeneity. Please report Q statistics and I^2 values, perform leave-one-cohort-out analyses, and consider alternative methods such as MANTRA or MR-MEGA for cross-ancestry meta-analysis.
3. The cluster cutoff was set at 1.057017, while the pPGS used 0.9554 without clear justification. Please explain the rationale for these differing thresholds and conduct sensitivity analyses using alternative values (e.g., 0.90, 0.9554, 1.057017, and "top-N per cluster").
4. Chromatin enrichment analyses appear thyroid-specific, whereas expression-based enrichment is not; yet colocalization

signals are strongest in thyroid tissue. Please reconcile these findings and provide gene-level examples to clarify this apparent discrepancy.
5. Several genes were tested using very few cis variants, which may reduce the reliability of posterior probabilities and introduce bias. Please re-run analyses using standard cis windows and ancestry-matched LD references. Additionally, consider methods that relax the single-causal-variant assumption (e.g., coloc.susie, pwcoco), and include regional association and colocalization plots to support key findings.
6. Please clarify whether observed TSH effects reflect feedback mechanisms or gland growth pathways. To address potential reverse causation due to treatment-induced TSH changes, consider employing instrumental variable (IV) approaches with TSH-specific genetic instruments.
7. Avoid overinterpreting the "Mixed function" cluster. Incorporate single-cell or tissue-specific annotations and perform leave-one-locus-out analyses to determine whether associations are driven by a small number of influential signals.
8. Given the limited number of medullary thyroid cancer (MTC) cases, I recommend treating MTC-related findings as exploratory.
9. Please make the primary analysis code publicly available, including scripts for meta-analysis result processing and the bNMF implementation (with details on random seed and initialization).

Minor Revisions:
1. Clarify whether LD pruning was applied during pPGS construction. In addition to weighted summation, indicate whether sparse (e.g., lasso) or Bayesian shrinkage models were evaluated to reduce overfitting.
2. There is a percentage typo in the subtype overlap description. In the Results section, “71% (165/2,334)” should be corrected to “7.1% (165/2,334).”
3. Include sensitivity analyses restricted to “pure PTC” and “pure FTC” cases, given the observed co-diagnoses in certain datasets.
4. Provide ancestry-specific Manhattan and volcano plots, along with a heatmap showing effect-direction concordance for sentinel variants.
5. Correct the title of Table S8 to: “Table S8. The number of cases used for individual-level association tests in All of Us data across ancestry groups.”
6. Some fraction cells in supplementary tables are incorrectly displayed as dates due to automatic formatting (e.g., "2/8" appears as "8-Feb"). Please correct these formatting issues.

Version 1:

Reviewer comments:

Reviewer #1

(Remarks to the Author)
The authors have extensively taken all of my remarks into account, including revised interpretations of results and extra analyses, which his has now led to a well-balanced manuscript. I am happy with the current version, and have no additional suggestions.

(Remarks on code availability)

Reviewer #2

(Remarks to the Author)
I appreciate the authors answers to my concern and do not have any further comments on the manuscript.

(Remarks on code availability)

Reviewer #3

(Remarks to the Author)
I thank the authors for their careful revisions in response to my previous comments. Overall, I believe that my main scientific concerns have now been largely addressed. However, before the manuscript is accepted, I recommend a small number of necessary minor revisions to ensure consistency across figure legends, and terminology, and to further moderate a few statements.
1.Please revise the wording in Figure 4 and its legend. The Methods section now correctly states that the colocalization analysis used all tested variants within the standard GTEx v10 cis-window, but the figure legend still refers to “significant eQTLs,” which may create confusion.
2.Please standardize the cluster terminology throughout the manuscript. The current text appears to use both “Thyroid Gland Dysfunction” and “Thyroid Gland Dysregulation,” and a single term should be used consistently.
3.I suggest slightly softening the discussion regarding clinical stratification, personalized treatment, and precision oncology. The current data support genetic heterogeneity and biological subclassification well, but direct clinical applicability remains to be established.

(Remarks on code availability)

Open Access This Peer Review File is licensed under a Creative Commons Attribution 4.0 International License, which permits use, sharing, adaptation, distribution and reproduction in any medium or format, as long as you give appropriate credit to the original author(s) and the source, provide a link to the Creative Commons license, and indicate if changes were made.
In cases where reviewers are anonymous, credit should be given to 'Anonymous Referee' and the source.
The images or other third party material in this Peer Review File are included in the article's Creative Commons license, unless indicated otherwise in a credit line to the material. If material is not included in the article's Creative Commons license and your intended use is not permitted by statutory regulation or exceeds the permitted use, you will need to obtain permission directly from the copyright holder.
To view a copy of this license, visit https://creativecommons.org/licenses/by/4.0/

REVIEWER COMMENTS

Reviewer #1 (Remarks to the Author):

Jee et al. performed a large GWAS to analyze the genetic drivers of multiple forms of thyroid cancer. Particularly, they analyzed whether there are various genetic clusters. I have a number of concerns:

1.From previous thyroid cancer GWAS results it has already been clear that part of the variants are known thyroid hormone regulation genes, while others are more involved in general oncogenic pathways. The fact that formal clustering strategies identify these clusters is therefore not surprising at all. Furthermore, one wonders what the follow up analyses really add to our knowledge: The fact that the thyroid hormone rgeulation cluster is associated with thyroid function or other thyroid diseases is neither suprising (actually it is known that these variants associate with many thyroid related endpoints - see previous GWAS on thryoid (dys)function). The authors for example identify an association with thyroid autonomy suggesting that thyroid autonomy may play a role in developing thyroid cancer. To gain more insight into this statement, the authors should perform much more analyses to bolster this statement, for example testing whether other thyroid autonomy related variants do or do not associate with thyroid cancer, and if so, why. The same holds true for hyperthyroidism (what is the role of Graves variants next to thyroid autonomy variants).

Response:
We agree with the reviewer that prior thyroid cancer GWAS have already indicated etiologic heterogeneity, with some loci mapping to thyroid hormone regulation and others to general oncogenic pathways. Accordingly, the identification of distinct mechanistic clusters is not unexpected. However, we emphasize that our study extends this literature in several important ways.

First, to evaluate whether the hormonally related cluster reflects broader thyroid disease liability, we performed additional, newly implemented follow-up analyses using large-scale GWAS of hyperthyroidism (primarily Graves disease) from a FinnGen-MVP-UKBB meta-analysis (E4_THYTOXGOITDIF). Of 49 independent hyperthyroidism lead variants, 42 were present in our thyroid cancer meta-analysis, and 9 showed nominal replication ($P < 0.05$) with concordant direction of effect, indicating partial genetic overlap (**Supplementary Figure 6a**). However, two-sample Mendelian randomization analyses provided no evidence for a causal effect of genetically predicted hyperthyroidism on thyroid cancer risk, with all estimators yielding non-significant results (**Supplementary Figure 6b**). Together with substantial heterogeneity and evidence of horizontal pleiotropy, these findings suggest that the observed overlap reflects shared pleiotropic loci rather than a unidirectional causal relationship. We emphasize that pleiotropy and causality represent distinct questions; the focus of this study is on characterizing

pleiotropic genetic architecture. These analyses results have now been included in Results, Methods, and Discussion.

Results: *"To evaluate whether these clusters reflect broader thyroid disease liability, we performed follow-up analyses using large-scale GWAS of hyperthyroidism (primarily Graves disease). Of 49 independent hyperthyroidism lead variants, 42 were present in our thyroid cancer meta-analysis, and 9 showed nominal replication (P < 0.05) with concordant direction of effect, indicating partial genetic overlap (Supplementary Figure 6a). However, two-sample Mendelian randomization analyses provided no evidence for a causal effect of genetically predicted hyperthyroidism on thyroid cancer risk, with all estimators yielding non-significant results (*β_{IVW}=0.06, p=0.23; *Supplementary Figure 6b, Supplementary Table 19)."*

Methods: *"To assess the relationship between hyperthyroidism and thyroid cancer, we performed analogous two-sample MR analyses using genome-wide association summary statistics for hyperthyroidism (*E4_THYTOXGOITDIF; *primarily Graves disease) from a FinnGen-MVP-UK Biobank meta-analysis(Kurki et al. 2023; Verma et al. 2024; Karczewski et al. 2025). Independent genome-wide significant lead variants were selected as instruments and harmonized with thyroid cancer summary statistics. In addition to MR analyses, we evaluated overlap between hyperthyroidism lead variants and thyroid cancer association results by examining concordance of effect directions and nominal significance. MR estimates were calculated using IVW as the primary method, with MR-Egger, weighted median, and weighted mode approaches as sensitivity analyses. Heterogeneity and pleiotropy were assessed using Cochran's Q and the MR-Egger intercept test, respectively. Results of the hyperthyroidism overlap and MR analyses are presented in Supplementary Figure 6 and Supplementary Table 19."*

Discussion: *"In our evaluation of whether the thyroid-related clusters reflect liability to clinical hyperthyroidism, we observed partial overlap between hyperthyroidism risk loci and thyroid cancer–associated variants; however, Mendelian randomization analyses did not support a causal effect of genetically predicted hyperthyroidism on thyroid cancer risk. These findings suggest that the shared loci likely represent pleiotropic genetic architecture influencing thyroid hormone regulation and cancer susceptibility through partially independent biological pathways, rather than a direct etiologic effect of hyperthyroidism on malignancy."*

Supplementary Figure 6

a. Replication of hyperthyroidism lead SNPs in thyroid cancer GWAS

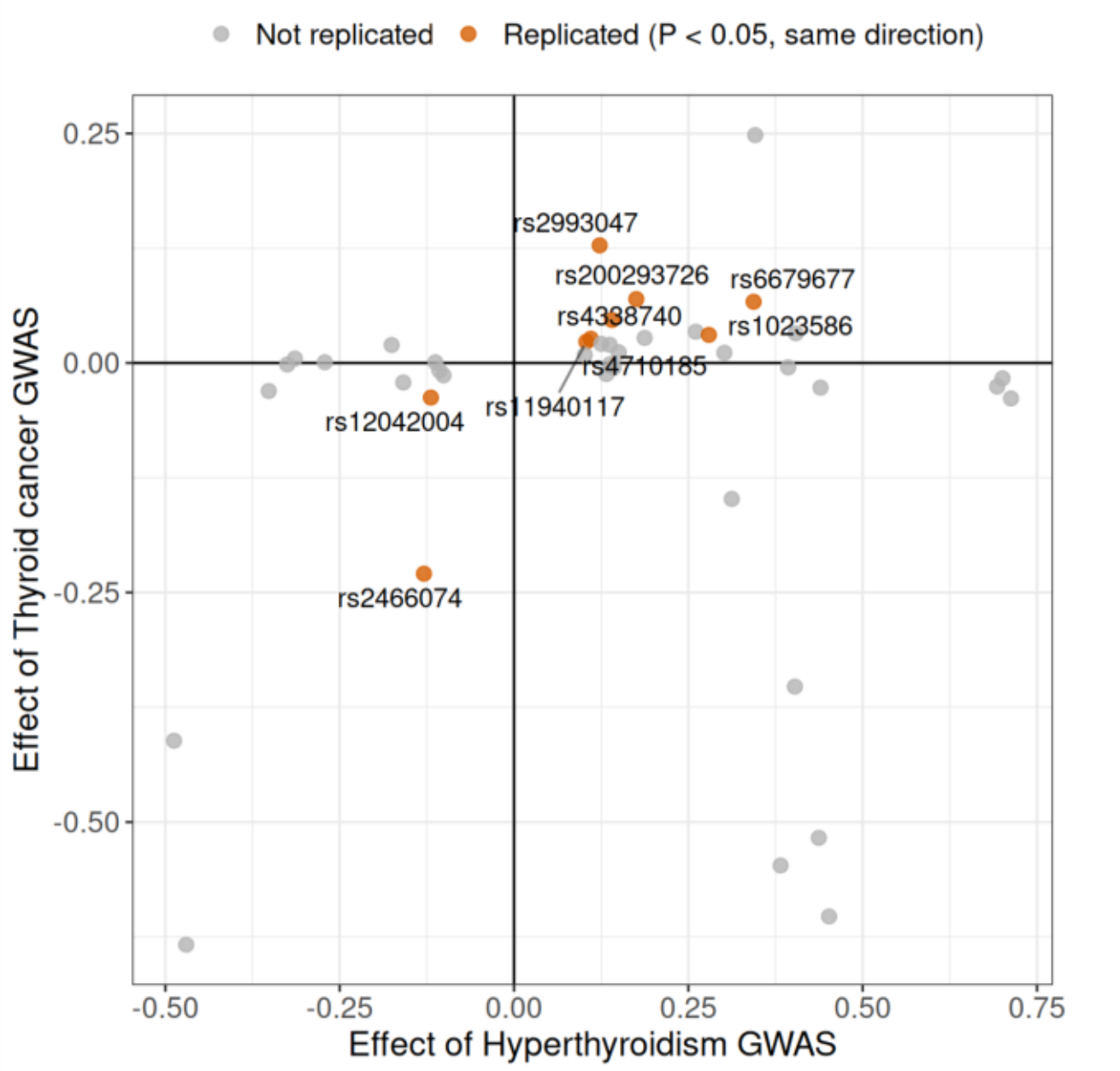

b. Mendelian randomization analysis between hyperthyroidism and thyroid cancer

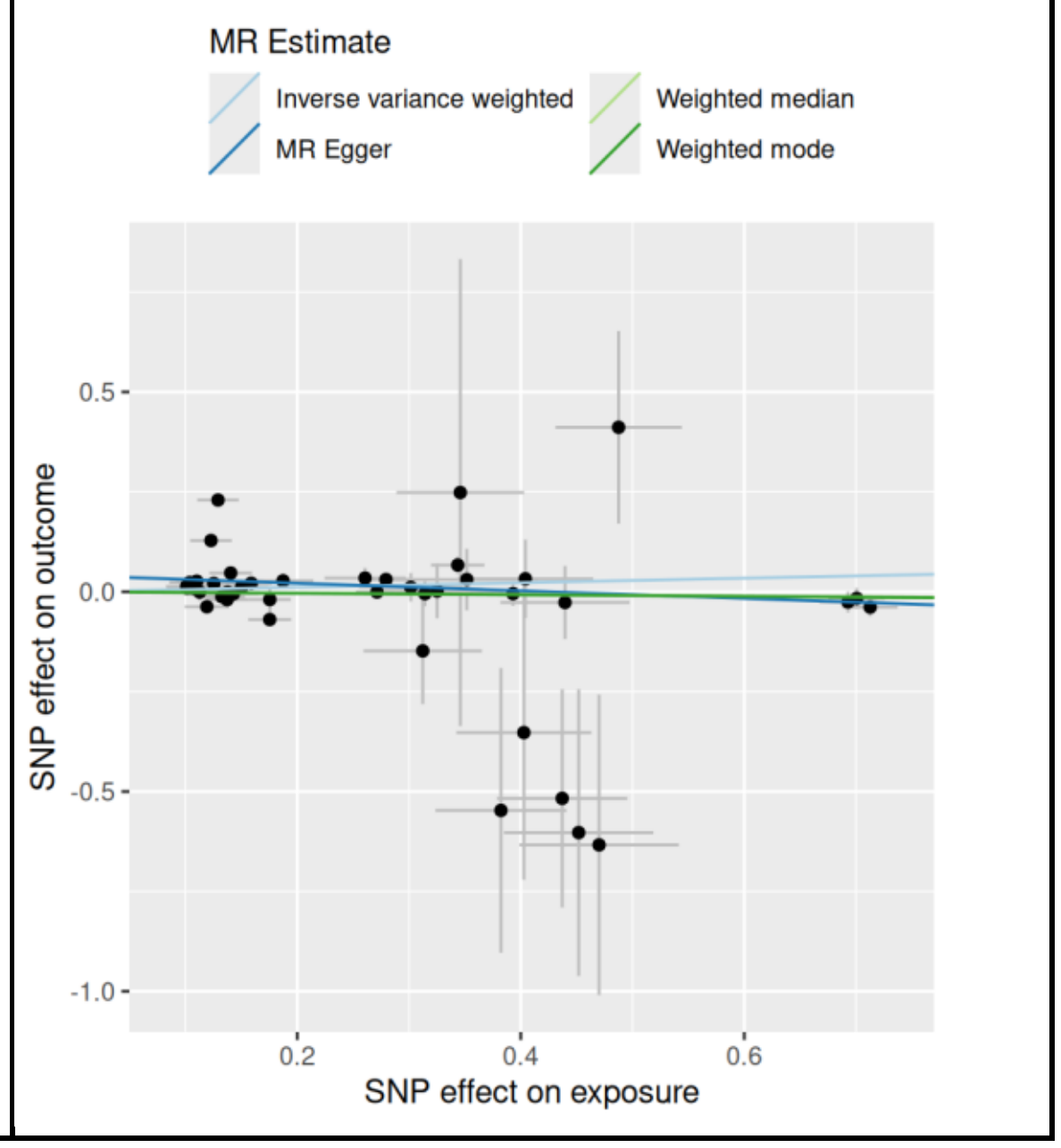

Second, while our findings are indeed consistent with those from previous studies, they are derived from a substantially larger and more diverse dataset. Our replication of hormonally related and oncogenic clusters across ancestries therefore provides additional support for the existence of distinct genetic drivers underlying etiologic heterogeneity in thyroid cancer, rather than reflecting study-specific artifacts.

Regarding thyroid autonomy, we agree that this concept requires careful interpretation. Thyroid autonomy refers to TSH-independent thyroid hormone production and is largely driven by somatic activating mutations (e.g., in *TSHR* or *GNAS*). As a result, thyroid autonomy is difficult to phenotype at scale; to our knowledge, no GWAS of thyroid autonomy is publicly available. Given these considerations, and the fact that Cluster 1 is primarily associated with decreased TSH rather than direct evidence of autonomous hormone production, we agree that our previous wording regarding thyroid autonomy could be interpreted as overstated. We have therefore revised the manuscript to remove statements implying a direct link to thyroid autonomy. The revised text now focuses on germline variants influencing thyroid hormone regulation and structural thyroid traits, without speculating on mechanistic interpretation.

> Removed: *"Collectively, this cluster likely captures structural thyroid changes associated with hormone overproduction, which may contribute to thyroid autonomy and tumor aggressiveness."*

2. The authors suggest that these pathways play a key role in thyroid cancer pathogenesis. What is the explained variance?

Response.

We appreciate the reviewer's question and the opportunity to clarify this point. By "key role," we do not mean that these pathways explain a large proportion of the total phenotypic variance in thyroid cancer risk. Rather, we use this term to reflect biological relevance and mechanistic importance, independent of the proportion of variance explained at the population level.

Using the 66 approximately independent lead SNPs, we estimated variance explained using the standard approximation $2p(1-p)\beta^2$, where p is the effect allele frequency and β is the log-odds ratio from logistic regression. For binary traits, this yields the genetic variance on the logit (linear predictor) scale. Summing across the 66 SNPs and converting to a pseudo-R^2 on the latent logistic scale, $R^2 = \frac{V_G}{V_G + \pi^2/3}$ gives an estimated variance explained of 15.95%.

In complex diseases, including cancer, individual loci or pathways can play critical roles in disease initiation or progression despite explaining only a small fraction of overall variance. A well-known example is *BRCA1/2* in breast cancer, which have profound biological and clinical importance while accounting for a modest proportion of population-level variance. Similarly, the pathways highlighted in our study are implicated based on consistent genetic associations, functional annotation, and prior experimental evidence, supporting their involvement in thyroid cancer biology even if their aggregate variance explained is limited.

Minor: The authors state that they substantially increased sample size compared to previous GWAS by analyzing 2.5 M subjects. However the far majority are controls (2.4 M).

Response.

We thank the reviewer for this point and agree that the total sample size should be interpreted in light of the case-control imbalance. Our statement is intended to reflect an increase in effective sample size and statistical power, not to imply that controls and cases contribute equally. While power gains diminish as the control-to-case ratio increases, additional controls still improve precision and contribute to the effective sample size.

For our study (16,167 cases and 2,430,374 controls), the effective sample size, calculated as $N_{eff} = \frac{4}{\frac{1}{N_{case}} + \frac{1}{N_{ctrl}}}$ is approximately 64,240. This is comparable to a balanced design with ~32,120 cases and is over double the effective sample size ~26,685 of the largest prior thyroid cancer meta-analysis from GBMI (Zhou et al. 2022). We have clarified this distinction in the text, as detailed below.

> Discussion: *"By increasing **effective** sample size and incorporating more diverse populations, …"*

Methods: *“The resulting effective sample size is approximately 64,240, which is more than double that of the next largest prior GWAS of thyroid cancer.33”*

Reviewer #2 (Remarks to the Author):

The authors of “Genetic drivers of etiologic heterogeneity in thyroid cancer” propose a new analysis of a large cohort of patients. They propose using non-negative matrix factorization to identify clusters associated with specific genetic processes and construct polygenic scores to try to better understand the genetic architecture of thyroid cancer.

Overall, I find the paper well written and well structured. The analysis is supported by functional validation with tissue enrichment, pathway analysis, and replication of cluster-specific polygenic scores in an external cohort.

Main comments:

1. Distributional assumptions: Ref. 115 provides several NMF estimation algorithms with automatic relevance determination of the rank K with the Itakura-Saito, Kullback-Leibler or Euclidean divergences. The authors used the Frobenius norm in this manuscript; however, this choice is not motivated. A discussion of which of these models is suitable for the data analyzed in this manuscript is lacking here. The underlying model is crucial when applying NMF as incorrect distributional assumptions can lead to the wrong number of clusters and not well-defined features.

Response:
The bNMF implementation used in this study uses the Euclidean divergence (Frobenius norm), which corresponds to a Gaussian noise model on the input matrix V (Tan and Févotte, 2013).

The input to the bNMF algorithm in this study was standardized GWAS summary statistics (z-scores), which are well-approximated by a Gaussian distribution, supporting the use of a Gaussian noise model.

Alternative divergences correspond to different implicit noise assumptions: the Kullback–Leibler divergence assumes Poisson-distributed count data, while the Itakura-Saito divergence assumes multiplicative noise and is scale-invariant. These assumptions are not well matched to the statistical properties of GWAS z-score matrices.

The Euclidean automatic relevance determination NMF model has been successfully applied to similar multi-trait genetic association matrices in prior work (Udler et al., 2018, Smith et al., 2024), supporting its suitability for the present analysis.

We have added a discussion of these distributional considerations and the rationale for using the Frobenius norm to the "Determining the optimal Number of Clusters (K) in bNMF" section in Supplementary Data:

> *"We used the Euclidean divergence, corresponding to a Gaussian noise model, which is appropriate for standardized GWAS association statistics. Alternative divergences (*Kullback–Leibler *or Itakura–Saito) assume count-based or multiplicative noise structures that are not consistent with the distributional properties of these data."*

2. Convergence:
1) the initial conditions are another important factor for NMF. The manuscript states that the algorithm is executed for 10,000 iterations with varying initial conditions. How were the initial conditions chosen? More details would be helpful here and would also help with the reproducibility of the analysis.
2) 10,000 iterations are usually sufficient for convergence in most NMF applications. A supplementary figure showing that this is also true in this particular case would be appreciated.

Response:
(1) Initialization

The final results were not based on a single initialization but on repeated bNMF runs of up to 10,000 iterations, with convergence monitored for each chain. Across 50 independent runs, the most frequently observed number of clusters (K) at convergence was selected as the final model, with support from posterior evidence. The distribution of converged K values is reported in the Supplementary Information. After selection of the optimal K, bNMF was re-run using this fixed value to obtain the final factorization.

In each bNMF run, the factor matrices W and H were initialized with nonnegative values from independently and identically distributed according to a uniform (0,1) distribution and scaled by the maximum value of the input matrix, following the original automatic relevance determination NMF formulation (Tan and Févotte, 2013) and prior applications in genetic clustering (Udler et al., 2018). These methodological details have been added to the "Determining the optimal Number of Clusters (K) in bNMF" section in Supplementary Data.

(2) Convergence
We thank the reviewer for this suggestion. We now include a convergence plot for the first bNMF run, showing stabilization of both the objective function and ARD relevance weights well before the 10,000-iteration limit, consistent with prior ARD-NMF work (Tan and Févotte, 2013) (**Supplementary Figure 13a**). In addition, a summary plot across all runs shows that convergence is achieved substantially earlier than 10,000 iterations for every random initialization, confirming that this iteration limit is conservative and sufficient (**Supplementary Figure 13b**). We have now added in the text:

> Methods: *"Convergence of the bNMF algorithm was assessed by monitoring the objective function and automatic relevance determination (ARD) relevance weights across iterations. In the primary run, both quantities stabilized well before the pre-*

specified 10,000-iteration limit, consistent with prior ARD-NMF implementations(Tan and Févotte 2013) (Supplementary Figure 13a). Across all random initializations, convergence was achieved substantially earlier than the maximum iteration cap, indicating that the 10,000-iteration limit was conservative and sufficient (Supplementary Figure 13b)."

Supplementary Figure 13

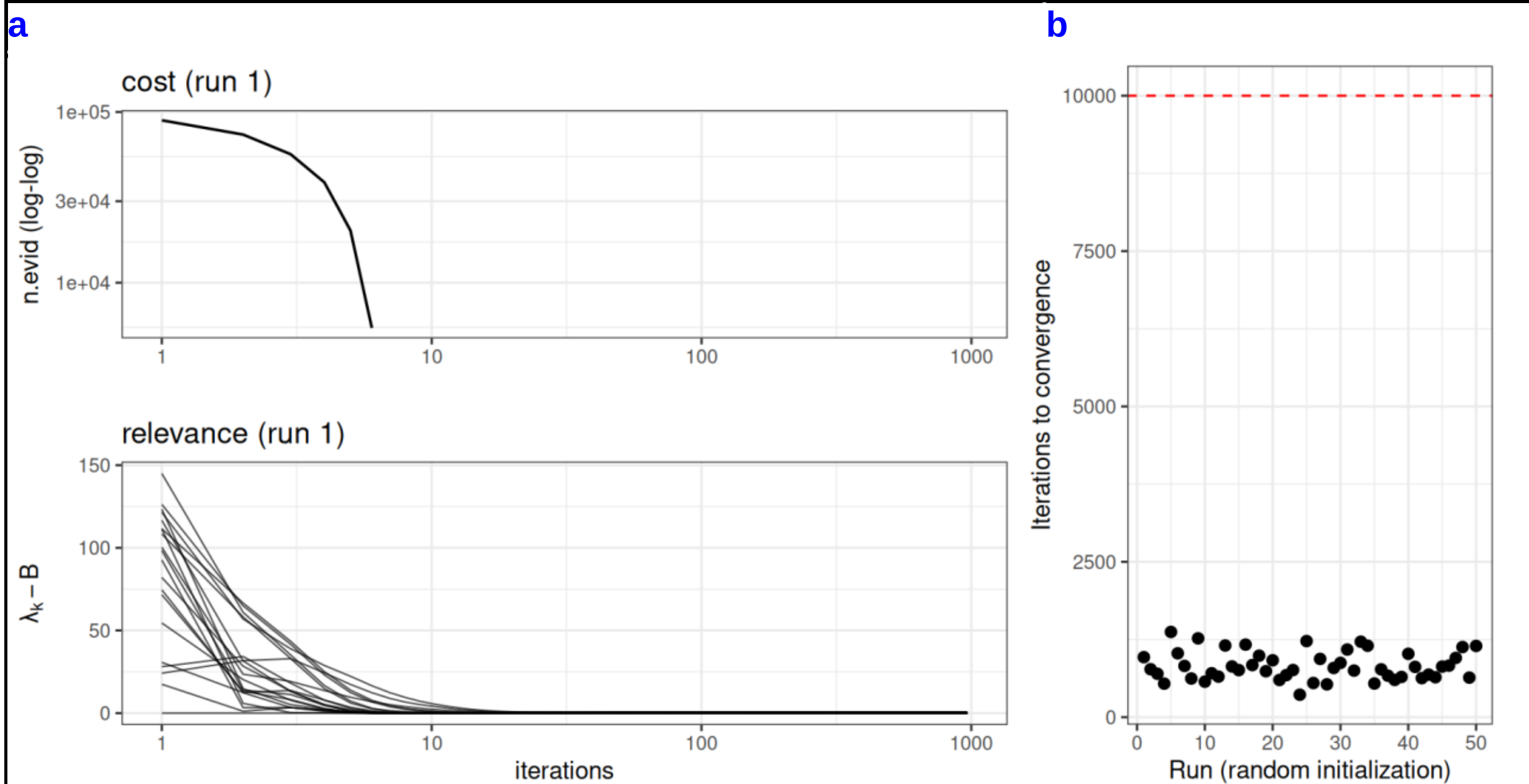

2A) Top: evolution of the negative log-posterior (n.evid) across iterations (log–log scale). Bottom: trajectories of ARD relevance weights λ_k − B across iterations (log-linear scale), where B denotes the baseline relevance level of pruned components. Both panels show convergence well before the maximum of 10,000 iterations. **2B)** Number of iterations required for convergence across 50 independent bNMF runs. All runs converged substantially before the maximum of 10,000 iterations (red dashed line), indicating that the iteration cap is conservative.

3. Visualization and interpretation of clusters:
1) NMF can often result in clusters that are difficult to interpret mainly because they share several of their key top-weighted components. Table 1 reports on the top weighted traits and loci for each cluster, it would be very helpful to have a visualization of such clusters to provide more insights into the separability of the observed clusters (e.g. by showing a PCA on the results of non-negative matrix factorization).
2) Reporting the number of variants per cluster before and after applying the cutoff for clustering weights would help readers in judging sparsity and interpretability of the clusters.

Response:
(1) Visualization

We agree with the reviewer that visualization is important for assessing cluster structure and interpretability. To address this, we now provide heatmap visualizations of both the variant-cluster (W) and trait-cluster (H) weight matrices derived from the bNMF. These heatmaps include hierarchical clustering and corresponding dendrograms, which allow direct assessment of similarity and separation across clusters.

As shown in these visualizations, variants and traits exhibit clear grouping by cluster, with limited overlap among the top-weighted components across clusters, supporting the separability suggested by the tabulated results in Table 1. While some degree of shared signal is expected given pleiotropy and correlated biological processes, the dominant structure is cluster-specific. We have added these heatmaps to the Supplementary Figure 4 and now explicitly reference them in the main text.

> Results: *"Because bNMF jointly factorizes the variant–trait association matrix into a variant loading matrix (W) and a trait loading matrix (H), each cluster is characterized by a specific pattern of contributions from both genetic loci and phenotypic traits. The loci with the highest weights in the W matrix and the traits with the highest weights in the H matrix define the dominant signals within each cluster and therefore provide insight into the putative underlying biological mechanisms (Supplementary Figure 4, Supplementary Table 5)."*

Supplementary Figure 4

a

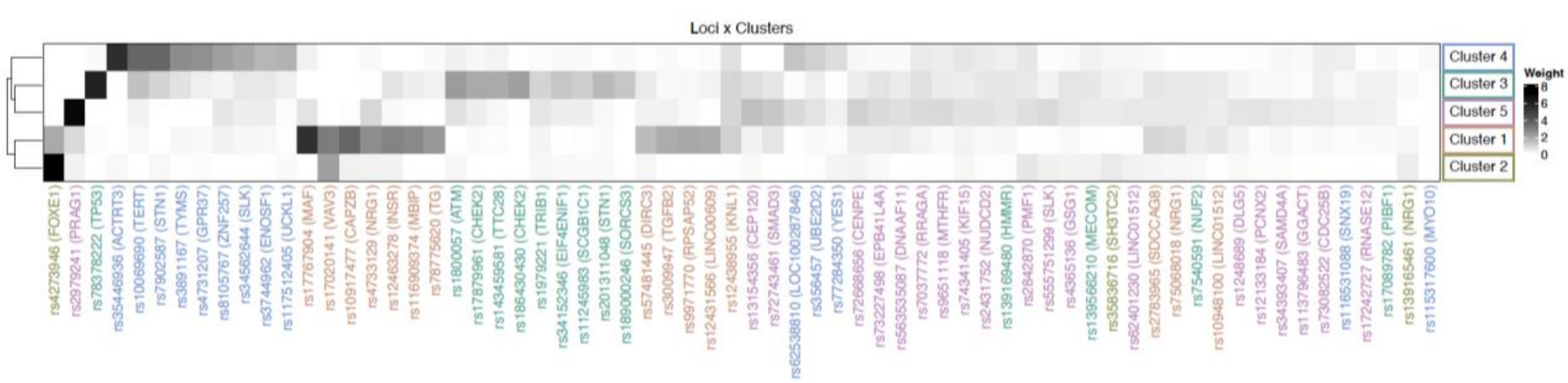

b*

Supplementary Figure 4

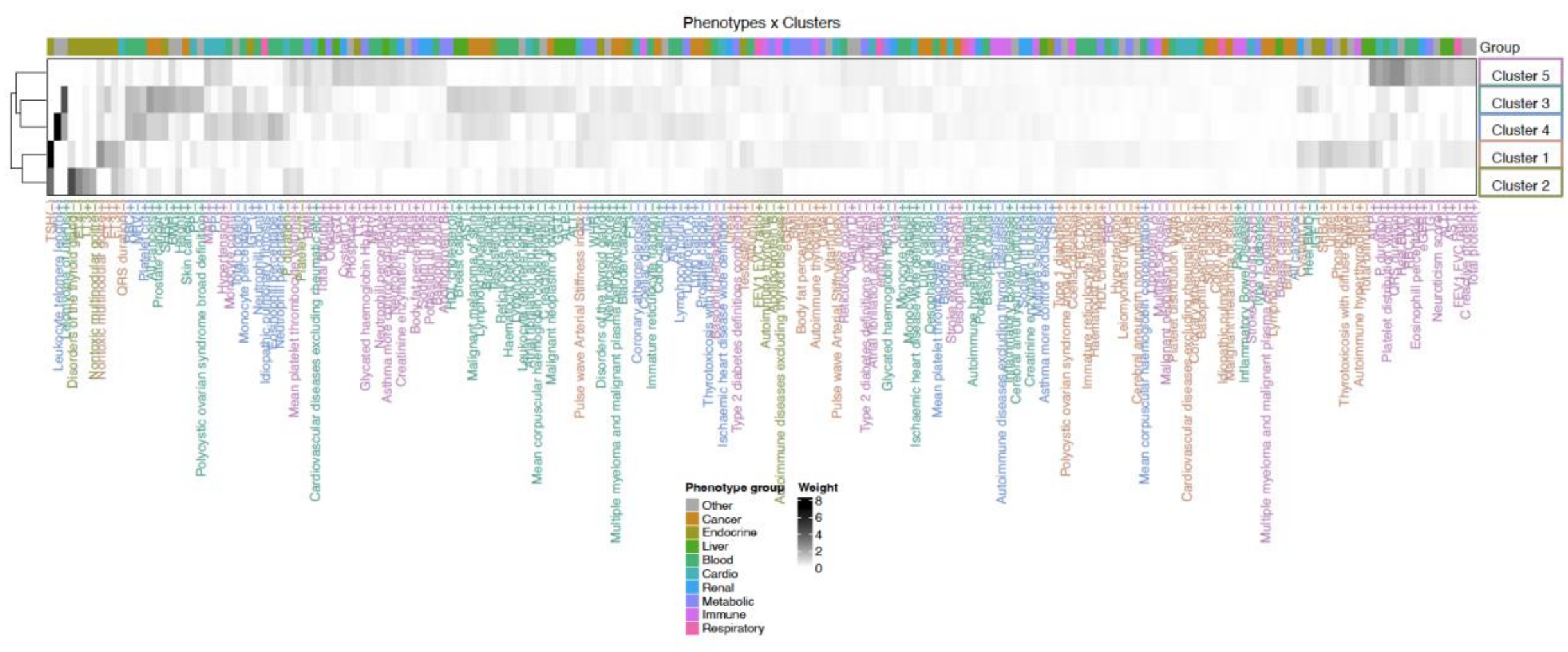

***In the main text (Supplementary Figure 4a–b), panel b is rotated to improve readability.**

(2) Cutoff

In Supplementary Table 5, we report the full variant-cluster weight matrix (W) for all thyroid cancer loci prior to thresholding. This table allows readers to identify, for each cluster, the number of variants with non-zero weights output directly by the bNMF algorithm, reflecting the soft-clustering structure before any post hoc filtering.

Cluster	#non-zero (before cut-off)	#above cut-off (1.057)
Cluster 1	60	12
Cluster 2	45	2
Cluster 3	60	10
Cluster 4	58	10
Cluster 5	54	3

4. Standardization: it is unclear whether results are robust to different normalizations of z-scores and why this procedure has been selected. Is this a standard procedure in the literature? In this case, I would suggest adding some references. Would it be possible to use standard variance-weighted Z-scores from GWAS?

Response:

We thank the reviewer for raising this important point regarding normalization and robustness of the standardized effect sizes used as input to the bNMF analysis.

The input matrix was constructed using standardized GWAS effect sizes defined as the ratio of the regression coefficient to its standard error ($\frac{\hat{\beta}_{ij}}{SE_{ij}}$), which corresponds to the standard Z-score widely used in GWAS summary-statistic analyses. This choice ensures comparability across traits while accounting for uncertainty in effect estimates.

Because the GWAS contributing to this study varied substantially in sample size, we further scaled these Z-scores by the square root of the effective study size, estimated as the mean sample size across SNPs for each trait. This adjustment reduces heterogeneity in signal magnitude driven purely by differences in sample size rather than by true genetic effects.

This normalization strategy follows established practice in the literature applying (Bayesian) NMF to GWAS summary statistics, including the original bNMF application to multi-trait genetic data (Udler et al., 2018, Smith et al., 2024), where sample-size-adjusted Z-scores were used to construct the variant-trait association matrix.

To assess robustness to the choice of normalization, we performed a sensitivity analysis comparing raw Z-scores and sample-size-adjusted Z-scores. As shown in the scatter plot below, these two representations were highly correlated (Pearson r > 0.95) (**Supplementary Figure 12**). Consistent with this, running bNMF using raw Z-scores yielded highly similar results, including the same number of inferred clusters and comparable biological interpretations (two thyroid-related clusters, two oncogenic clusters, and one mixed-function cluster), as shown in the table below (**Supplementary Table 16**). We have added the following to the main text:

> Discussion: *"To evaluate the robustness of our findings, we conducted several complementary sensitivity analyses. Specifically, we assessed, …, (iii) the effect of alternative normalization procedures for the variant–trait association matrix, observing stable cluster structure across scaling approaches (Supplementary Table 16, Supplementary Figure 12). These analyses indicate that the inferred pleiotropic architecture is robust to key analytic decisions inherent to bNMF-based clustering."*
> Methods: *"This standardization strategy follows established practice in the literature applying NMF to GWAS summary statistics, including the original bNMF application to multi-trait genetic data(Udler et al. 2018; Smith et al. 2024), where sample-size-adjusted Z-scores were used to construct the variant-trait association matrix (Supplementary Data). To assess robustness to the choice of normalization, we performed a sensitivity analysis comparing raw Z-scores and sample-size-adjusted Z-scores (Supplementary Table 16, Supplementary Figure 12)."*

Supplementary Figure 12. Scatter plot comparing raw Z scores and scaled Z scores. Values from 66x105 matrix after removing 46 low p-value/correlated trait values.

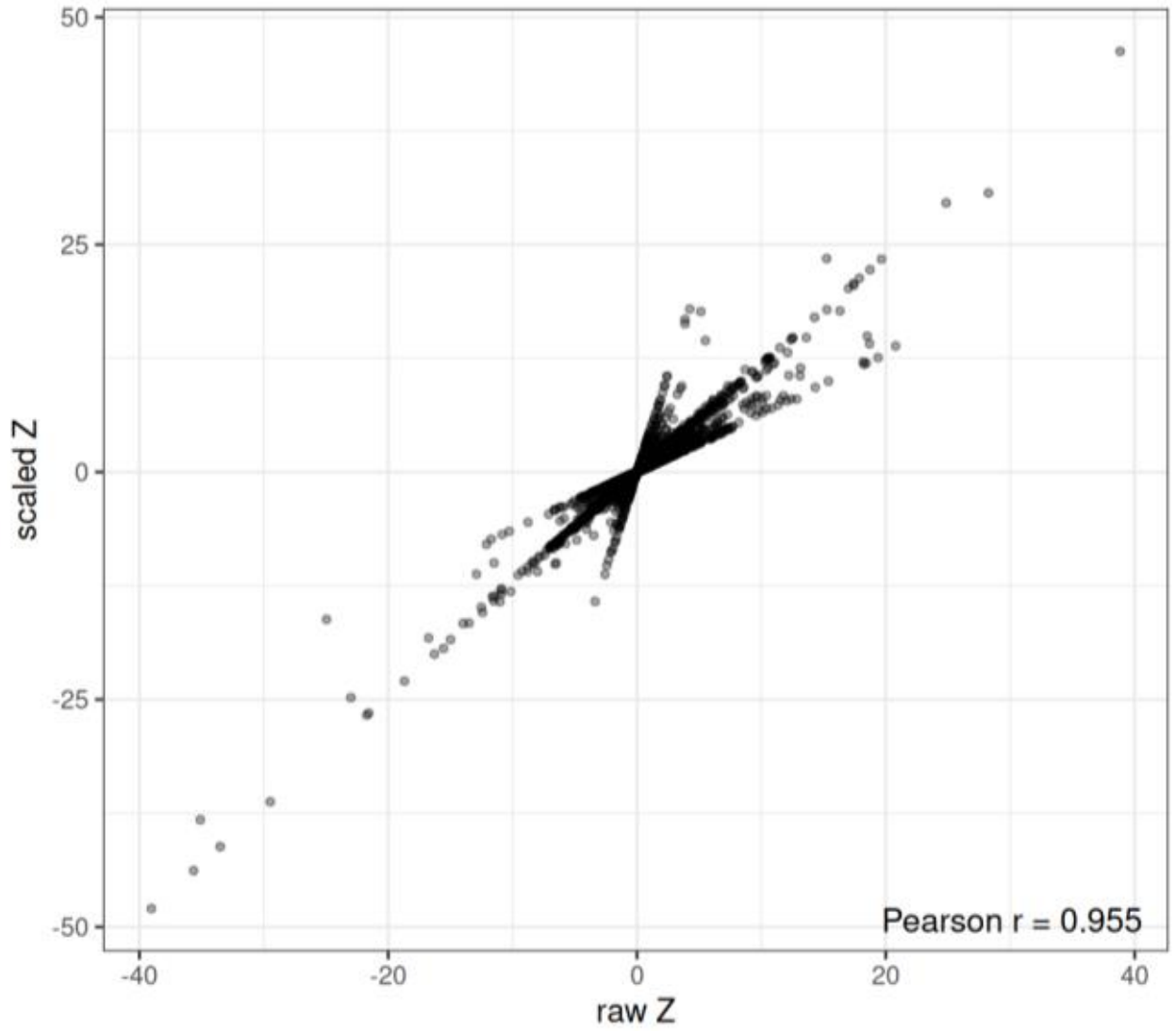

Supplementary Table 16. Clustering result using raw Z-score as input to bNMF

Cluster	#variants	Key.top.weighted.traits	Key.top.weighted.loci
Cluster1	11	TSH(-), Nontoxic.multinodular.goitre(+), FT4(+), Thyrotoxicosis.with.diffuse.goitre(+), Urate(-)	MAF, CAPZB, INSR, MBIP, TG, NRG1, VAV3, LINC00609, TGFB2, RPSAP52, DIRC3
Cluster2	2	TSH(-), Disorders.of.the.thyroid.gland(-), Nontoxic.multinodular.goitre(-), FT4(-)	FOXE1, VAV3
Cluster3	9	Leiomyoma.of.uterus(+), All.cancers(+), Skin.cancer(+), Breast.cancer(+), Height(+), Prostate.cancer(+), Basal.metabolic.rate(+), SHBG(-), Lymphoid.leukaemia(+), Polycystic.ovarian.syndrome..broad.definition(+), Malignant.melanoma.of.skin(+), Brain.cancer(+), Red.blood.cell..erythrocyte..count(+), Mean.reticulocyte.volume(-), Pulse.pressure..automated.reading(-)	TP53, CHEK2, TTC28, ATM, EIF4ENIF1, STN1, SORCS3
Cluster4	10	Leukocyte.telomere.length(+), Leiomyoma.of.uterus(+), Idiopathic.pulmonary.fibrosis(-), Mean.reticulocyte.volume(-), Platelet.crit(+), Red.blood.cell..erythrocyte..count(+), Hypertension(+), Eosinophill.percentage(-), Nontoxic.multinodular.goitre(+), Neutrophill.percentage(+), Total.protein(-), All.cancers(+), Monocyte.percentage(-)	ACTRT3, TERT, STN1, TYMS, GPR37, ZNF257, SLK, UCKL1, ENOSF1, LOC100287846
Cluster5	3	Alkaline.phosphatase(+), Platelet.distribution.width(+), Heel.bone.mineral.density..BMD.(+), Red.blood.cell..erythrocyte..distribution.width(+), Triglycerides(-), Hypertension(+),	PRAG1, SMAD3, CEP120

Supplementary Table 16. Clustering result using raw Z-score as input to bNMF

		Asthma..more.control.exclusions.(+), Estimated.glomerular.filtration.rate(+), Aspartate.aminotransferase(+), Eosinophill.percentage(+), Neuroticism.score(-), Alanine.aminotransferase(+), BMI(+), Gamma.glutamyltransferase(+), Mean.arterial.pressure..automated.reading(+)	

- Removal of highly correlated traits (lines 569-571): the reason for this step is not clear to me. Please motivate this further.

Response:
The bNMF framework aims to identify latent clusters that capture distinct patterns of variant-trait associations across multiple traits. Including highly correlated traits can introduce redundancy in the input matrix, effectively over-weighting a single biological signal and biasing the inferred factorization toward traits that are near-duplicates of one another.

When multiple traits are strongly correlated at the level of GWAS summary statistics, they often reflect overlapping phenotypic definitions, shared measurement constructs (eg, ratios), or closely related clinical endpoints. Treating such traits as independent inputs can artificially inflate their contribution to the reconstruction error term and distort cluster relevance weights. To mitigate this issue, we removed traits with high pairwise Pearson correlation ($r > 0.80$) computed across the set of index variants and retained the trait with the stronger overall genetic signal.

We have expanded the Methods section to better motivate this step and clarify its role in preventing redundancy-driven bias in the bNMF clustering:

> *"... Because the bNMF framework is designed to identify latent clusters representing distinct patterns of variant-trait associations, inclusion of highly correlated traits can introduce redundancy and effectively over-weight a single underlying biological signal, biasing the factorization."*

- Procedure for handling missing data (lines 579-580): are any of the results from Supplementary Table 11 contradicting expectations or showing new interesting traits? I do not have the expertise to judge this, but a comment on whether or not imputation provided new insights on the clusters would also add value to this step of the analysis.

Response:
We thank the reviewer for raising this point. Importantly, standard Bayesian non-negative matrix factorization does not accommodate missing values in the input matrix; therefore, in practice, some form of imputation or masking strategy is required to enable clustering when summary statistics are incomplete across traits. Our proxy-based imputation approach was implemented

to address this methodological constraint and to evaluate the robustness of the clustering solution to the handling of missing data.

The clustering structure obtained under proxy-based imputation was highly consistent with the primary analysis. Specifically, key biological clusters, including thyroid function, DNA damage response, telomere maintenance, and mixed metabolic/hematologic processes, were preserved. The majority of loci also mapped to analogous clusters across analyses, demonstrating robustness to the handling of missing data, as shown in **Supplementary Table 11**.

Minor comments:

- Despite being a heuristic approach, I believe the cutoff for clustering weights determination can be an efficient solution for interpretability of the clusters. My understanding is that this is defined with an “elbow”-like approach. Cross-validation could be a more rigorous alternative for this, it might be interesting to study whether this would provide a different threshold.

Response:
We agree with the reviewer that the thresholding of clustering weights is a heuristic choice primarily intended to improve interpretability. As described in the Supplementary Information, we adopted the long-tail analysis approach originally proposed by Udler et al. (2018), which examines the empirical distribution of bNMF weights and identifies a natural separation between strongly contributing and weakly contributing variants, analogous to an elbow-type criterion. This approach has been used in prior applications of bNMF and facilitates consistent interpretation of cluster-defining signals.

We considered cross-validation as a potential alternative; however, in the bNMF setting there is no well-defined predictive loss function for selecting a weight cutoff, as the factorization is unsupervised and the weights are not optimized for out-of-sample prediction. Moreover, the thresholding step does not affect the underlying factorization itself, but rather serves as a post hoc interpretability aid. For transparency, we therefore report the full, unthresholded clustering weights in the Supplementary Table 5, allowing readers to assess the robustness of cluster membership across a range of potential thresholds.

- In my view, Figure 3 and 4 are very dense. Figure 3 might benefit from either a re-organization or a clearer connection between panels a-c and panel d. Figure 4 could be moved to supplements and could be replaced with a schematic summary of the current figure with main takeaways.

Response:
We agree that the original presentation of **Figures 3 and 4** was dense. To improve clarity, we have re-organized **Figure 3** by splitting it into two figures and revising the legends and text to better explain the connection between panels.

We considered moving **Figure 4** to the Supplementary Information, but we retain it in the main text because it summarizes results central to the study's primary conclusions. However, we have simplified the figure to display only the results explicitly discussed in the manuscript. This revised figure now appears as **Figure 5**, and the complete set of original results has been moved to **Supplementary Figure 11**.

Figure 5 (previous Figure 4)

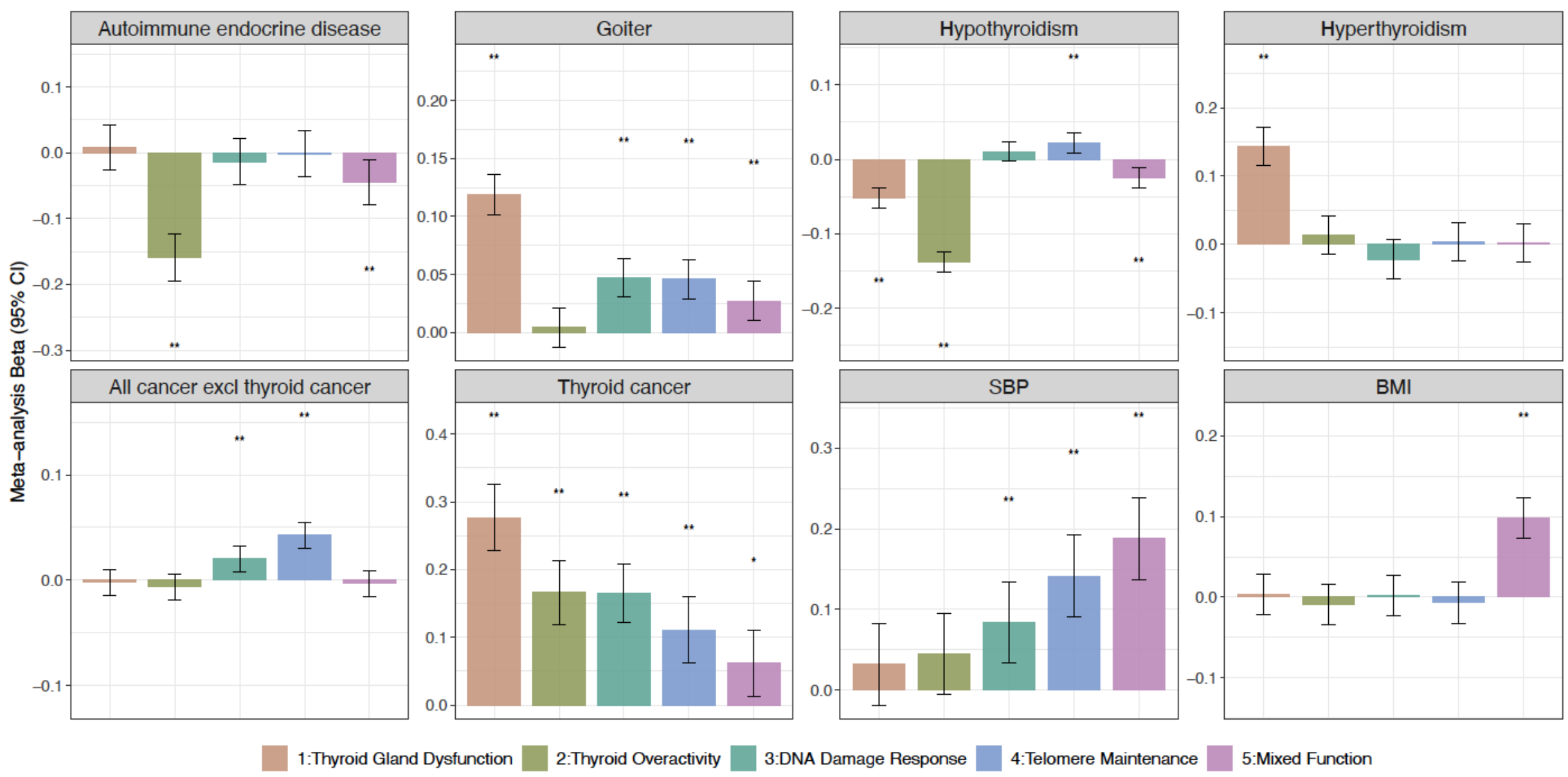

- Notation: in the NMF literature "features" is usually referring to what is termed as "clusters" in this manuscript. I would suggest including a comment on this as it can be confusing at first glance. References to older works on NMF such as Lee and Seung 2004 can also be included in this context.

Response:

We thank the reviewer for pointing out this potential source of confusion. To clarify this terminology, we have added an explicit statement in the Methods section noting that we use the term "cluster" throughout for interpretability in the genetic context:

> *"In the NMF literature, latent components are often referred to as 'features' (Lee and Seung, 1999); here, we use the term 'cluster' to reflect the biological interpretation of these components as groups of variants and traits with shared genetic associations."*

- The mathematical formulation of the standardized z-score in the supplements is quite blurry. Could be written in-text instead of adding an image.

Response:

We thank the reviewer for noting that the mathematical formulation of the standardized z-score in the Supplementary Materials is difficult to read. We have removed the blurry image and replaced it with an in-text equation for clarity.

> *… This transformation resulted in the variant-trait association matrix $Z \in R^{N \times T}$ (66 variants × 151 traits).*
>
> *Mathematically:*
>
> $$Z'_{ij} = \frac{Z_{ij}}{\sqrt{N_{ij}}} \times S, where\ S = \frac{1}{T}\sum_{j=1}^{T} \sqrt{median_i(N_{ij})}$$

- *Z'_{ij} is the adjusted z-score for variant i and trait j.*
- *Z_{ij} is the original z-score.*
- *N_{ij} is the sample size for variant i and trait j.*
- *S is the scaling factor computed earlier*

Reviewer #3 (Remarks to the Author):

This manuscript presents a multi-ancestry genome-wide investigation of thyroid cancer susceptibility, combining data from 18 studies. The authors identified 66 independent signals across 51 loci and integrated cross-trait evidence from 151 immune, metabolic, endocrine, and hematopoietic phenotypes. Using Bayesian non-negative matrix factorization, they categorized the signals into five putative mechanistic clusters and developed cluster-specific polygenic scores, which were externally validated in the All of Us cohort. Complementary functional analyses, including tissue/cell-type enrichment, eQTL colocalization, and genomic annotation, implicated pathways related to telomere maintenance, DNA damage response, thyroid biology, and autoimmunity. Overall, while the study leverages impressive datasets and a broad analytical scope, it would benefit from stronger methodological justification, sensitivity analyses, and clearer integration of functional evidence to enhance both novelty and robustness.

Major Revisions:

1. Independence of signals was defined using a Finnish LD reference panel and a threshold T, with additional steps applied when T < 0.02—an unconventional approach. Please justify the choice of reference panel, include sensitivity analyses using cosmopolitan or ancestry-specific LD panels, and compare results with standard clumping or conditional analysis methods.

Response:

We appreciate the reviewer's request for clarification regarding the LD reference panel and the dynamic thresholding strategy used to define independent signals.

As an initial comparison, we applied standard LD clumping using a fixed threshold ($r^2 > 0.1$) and the 1000 Genomes Phase 3 ALL (cosmopolitan) reference panel. This approach identified 186 lead variants across 48 loci (Table 1). However, at loci with extremely strong associations, fixed-threshold clumping produced an implausibly large number of putatively independent signals. For example, at the *FOXE1* locus, 36 "independent" variants were identified, despite all associations being driven by a single lead variant (rs4273946; $p = 6.07 \times 10^{-235}$). This pattern is consistent with residual LD around exceptionally strong GWAS peaks rather than true allelic heterogeneity, a known limitation of fixed-threshold clumping in high-powered GWAS.

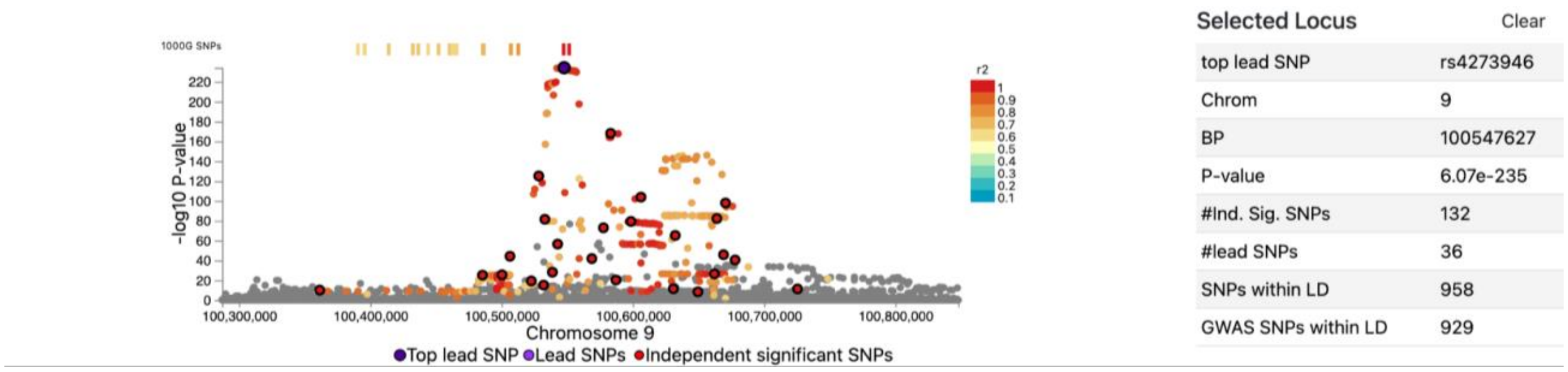

To mitigate this issue, we adopted a dynamic LD thresholding strategy in which the stringency of LD pruning scales with the strength of the primary association signal. This approach substantially reduces LD-induced secondary associations while retaining genuinely independent effects. Similar strategies have been applied in recent large-scale clustering and fine-mapping studies (e.g., Reeve et al., 2024), supporting its methodological appropriateness in settings with extreme association statistics.

Dynamic clumping was implemented using the autoreport software developed by the FinnGen consortium, which is specifically designed for this purpose and optimized for use with the Finnish LD reference panel. Resolution of independence is particularly important in our study because the resulting set of lead variants directly informs downstream pleiotropy-based clustering; inflation of secondary signals due to strong LD with lead variants can propagate into spurious clusters and misleading biological interpretations. We therefore prioritized minimizing false-positive independent signals at this stage.

The Finnish LD reference panel was chosen for two reasons. First, autoreport is calibrated for this panel, ensuring stable and internally consistent LD estimates under the dynamic thresholding framework. Second, a substantial proportion of the contributing GWAS data are from FinnGen–it contributes 18% of the total effective sample size, the 2nd largest contribution.

We acknowledge that ancestry-specific LD references would be ideal for a multi-ancestry meta-analysis. However, this is not practically feasible here, as discovery statistics were obtained from a combined meta-analysis of cohorts with differing ancestry compositions (e.g., FinnGen, UK Biobank, and the multi-ancestry MVP), precluding consistent application of ancestry-stratified LD across all variants.

To directly address the reviewer's concern, we performed sensitivity analyses using alternative LD references and clumping strategies. Specifically, we compared dynamic clumping results to fixed-threshold clumping using the 1000 Genomes Phase 3 ALL panel under two thresholds: (i) the standard $r^2 > 0.1$, and (ii) a stringent threshold ($r^2 = 0.005$) corresponding to the minimum LD threshold induced by the strongest signal in the dynamic framework (rs4273946). As shown in **Supplementary Table 17**, dynamic clumping markedly reduces spurious secondary signals at highly significant loci while preserving locus-level discoveries.

We further reran the full clustering analysis using 85 lead variants obtained from fixed-threshold clumping with $r^2 = 0.005$ and the 1000 Genomes ALL reference panel. This sensitivity analysis yielded a highly concordant clustering structure (**Supplementary Table 18**) compared with the primary analysis (**Table 1**). Specifically, five clusters were again identified, including two thyroid-related clusters, two cancer-related clusters (DNA damage response and telomere maintenance), and one mixed-function cluster. The top-weighted traits and core driver loci within the thyroid and cancer-related clusters were largely preserved, including canonical genes such as *FOXE1, TERT, STN1, TP53, and CHEK2*. The principal difference relative to the primary results is observed in Cluster 5 (mixed-function). Under the stringent fixed-threshold clumping strategy, this cluster includes additional loci (e.g., *MTMR9, MSRA, ERI1, DEFB134, TNKS, XKR6*), reflecting modest redistribution of variants across clusters. Notably, however, the overall biological interpretation of this cluster—characterized by metabolic, hematologic, and systemic biomarker traits—remains consistent with the primary analysis.

These results demonstrate that the qualitative conclusions of our clustering analyses are robust to the choice of LD reference panel and clumping strategy. We have clarified the rationale for our LD reference selection and thresholding strategy in the Methods and now explicitly report the results of these sensitivity analyses, as follows:

> Methods: *"LD was estimated using the Finnish LD reference panel (SISu v4.2), for which the autoreport framework is calibrated and optimized. To assess robustness to LD reference choice and signal definition strategy, we conducted sensitivity analyses using standard fixed-threshold LD clumping with the 1000 Genomes Phase 3 ALL (cosmopolitan) reference panel under both conventional ($r^2 = 0.1$) and stringent ($r^2 = 0.005$) thresholds. We additionally reran downstream clustering analyses using lead variants defined under the stringent fixed-threshold approach. These sensitivity analyses yielded qualitatively similar locus-level and clustering results, indicating that our primary conclusions are robust to the choice of LD reference panel and clumping strategy (Supplementary Tables 17-18)."*

**Supplementary Table 17**			
	**Clumping method**		
**Reference panel**	**Standard (fixed LD r2>0.1)**	**Dynamic**	**Standard (fixed LD r2>0.005)**
**FinnGen**	205 lead variants (52	66 lead variants (51	90 lead variants (52

Supplementary Table 17			
	loci)	loci)*	loci)
**1000 Genome Phase 3 ALL (Cosmopolitan)**	194 lead variants (50 loci)	130 lead variants (58 loci)	85 lead variants (50 loci)

*Includes additional refinement through fine-mapping analyses in FinnGen to further streamline the list.

Supplementary Table 18			
**Table 2B**			
Cluster (Original cluster)	Number.of. variants	Key.top.weighted.traits	Key.top.weighted.loci
Cluster4	10	Leukocyte.telomere.length(+), Leiomyoma.of.uterus(+), Platelet.crit(+), Idiopathic.pulmonary.fibrosis(-), Mean.reticulocyte.volume(-), Red.blood.cell..erythrocyte..count(+), Eosinophill.percentage(-), Skin.cancer(+), Nontoxic.multinodular.goitre(+), Brain.cancer(+), Hypertension(+), All.cancers(+), Total.protein(-), Lymphoid.leukaemia(+), Lung.cancer(+), Neutrophill.count(+)	ACTRT3, TERT, STN1, TYMS, GPR37, LRRC31, ZNF257, UCKL1, LOC100287846
Cluster2	7	Disorders.of.the.thyroid.gland(-), TSH(-), Nontoxic.multinodular.goitre(-), FT4(-), Height(+)	FOXE1, VAV3, ANP32B, HABP4, TMOD1
Cluster3	11	Leiomyoma.of.uterus(+), All.cancers(+), Skin.cancer(+), Height(+), Breast.cancer(+), Basal.metabolic.rate(+), Prostate.cancer(+), SHBG(-), Polycystic.ovarian.syndrome..broad.definition(+), Lymphoid.leukaemia(+), Malignant.melanoma.of.skin(+), Mean.reticulocyte.volume(-), Brain.cancer(+), Atrial.fibrillation.and.flutter(+), Pulse.pressure..automated.reading(-), Red.blood.cell..erythrocyte..count(+)	TP53, CHEK2, C11orf65, TTC28, EXO1, EMID1, ZNRF3, SORCS3, STN1, HABP4
Cluster1	13	TSH(-), Nontoxic.multinodular.goitre(+), Thyrotoxicosis.with.diffuse.goitre(+), FT4(+), SHBG(+), Urate(-)	MAF, INSR, CAPZB, TG, VAV3, NRG1, TGFB2, RPSAP52, LINC00609, MBIP, DIRC3
Cluster5	7	Alkaline.phosphatase(+), Red.blood.cell..erythrocyte..distribution.width(+), Hypertension(+), Triglycerides(-), Estimated.glomerular.filtration.rate(+), BMI(+), Neuroticism.score(-), Eosinophill.percentage(+), Asthma..more.control.exclusions.(+), Nontoxic.multinodular.goitre(+), Diastolic.blood.pressure..automated.reading(+), Gamma.glutamyltransferase(+),	MTMR9, MSRA, PRAG1, ERI1, DEFB134, TNKS, XKR6

Supplementary Table 18			
		Type.2.diabetes..definitions.combined(+), Total.protein(+), Glycated.haemoglobin..HbA1c.(+), Basal.metabolic.rate(+), Phosphate(+), Mean.platelet..thrombocyte..volume(+), Neutrophill.count(-), FEV1.FVC.ratio(-), Height(-), Monocyte.count(-), Red.blood.cell..erythrocyte..count(+), Mean.reticulocyte.volume(+), Pulse.pressure..automated.reading(+)	

2. The use of fixed-effect meta-analysis may not be appropriate in the presence of cross-cohort or cross-ancestry heterogeneity. Please report Q statistics and I^2 values, perform leave-one-cohort-out analyses, and consider alternative methods such as MANTRA or MR-MEGA for cross-ancestry meta-analysis.

Response:
We appreciate the reviewer's concern regarding potential heterogeneity in fixed-effect meta-analysis. To directly address this, we have implemented additional heterogeneity assessments and clarify the rationale for our chosen meta-analytic framework.

First, to quantify heterogeneity, we now report Cochran's Q statistics and I^2 values for the lead variants at each locus (**Supplementary Table 2**). Across 66 lead variants, heterogeneity was generally limited. Most loci showed no evidence of heterogeneity (53/66, 80.3% with HetPVal>0.05; 61/66, 92.4% with HetPVal>0.01). The median I^2 was 0% (IQR 0–50.8%), with 43/66 (65.2%) exhibiting low heterogeneity ($I^2 < 25\%$). The updated heterogeneity metrics are included in the revised **Supplementary Table 2**.

Second, we agree that leave-one-cohort-out analyses are useful for assessing cohort-driven effects. However, such analyses are not feasible in our study, as individual biobank–level summary statistics are not uniformly available for all contributing cohorts. In several instances, only meta-analyzed summary statistics are accessible, precluding systematic exclusion of individual cohorts while preserving consistent variant coverage and effect estimates.

Regarding alternative cross-ancestry meta-analysis methods, we acknowledge their utility in modeling ancestry-related heterogeneity. However, these methods require ancestry-specific summary statistics and/or allele frequency information across multiple ancestral strata, which are not available in a harmonized form for our contributing datasets. For example, our analysis integrates results from FinnGen, UK Biobank (only European ancestry), and MVP (multi-ancestry), where ancestry-specific GWAS results are not consistently available across all cohorts (https://finngen.gitbook.io/documentation/methods/meta-analysis).

Finally, we emphasize that the fixed-effect model is a valid test even in the presence of heterogeneity and has better or comparable power to ancestry-aware methods in the presence of low or moderate heterogeneity, as suggested by the observed Q and I^2 statistics. Importantly, multiple studies have estimated that cross-ancestry effect sizes are highly consistent for the same trait using different methods ($r_g > 0.95$ on average) (Hou et al. *Nat Gen* 2023. PMID: 36941441; Hu et al. *Nat Gen* 2025. PMID: 39901012). While ancestry-aware meta-analysis methods can provide additional insights when suitable inputs are available, their application is beyond the scope of the present study given current data constraints.

3. The cluster cutoff was set at 1.057017, while the pPGS used 0.9554 without clear justification. Please explain the rationale for these differing thresholds and conduct sensitivity analyses using alternative values (e.g., 0.90, 0.9554, 1.057017, and "top-N per cluster").

Response:
The clustering weight cutoff of 1.057017 is the correct and consistently applied threshold throughout the analysis. The value 0.9554 reported was a typo, which we have now corrected in the revised manuscript.

As described in the Supplementary Information, the cutoff of 1.057017 was determined using a long-tail ("elbow"-type) analysis of the distribution of clustering weight deltas, following the approach introduced by Udler et al. (2018). This procedure identifies a natural separation between strongly contributing variants and those with marginal contributions and is intended as a pragmatic interpretability step for soft bNMF clusters.

The same cutoff was used consistently both for defining cluster membership and for constructing cluster-specific polygenic scores, ensuring methodological coherence between the clustering and downstream analyses. Importantly, to ensure transparency and flexibility, clustering weights for all thyroid cancer lead variants are provided in Supplementary Table 5, enabling other researchers to apply alternative thresholds or top-N per-cluster selection strategies according to the needs of their downstream analyses.

We agree that alternative thresholds could in principle be explored. However, the cutoff does not affect the underlying bNMF factorization or the number of clusters inference, which are determined independently by the automatic relevance determination framework. Rather, it influences which variants are highlighted as primary contributors for interpretation and downstream summarization. We have clarified this in the manuscript as follows:

> *"The cluster membership cutoff (1.057017) was determined using a long-tail ('elbow') analysis of clustering weight deltas and was applied consistently across cluster definition, enrichment analysis, and pPGS construction; this threshold affects interpretative labeling of strongly contributing variants but does not alter the underlying bNMF factorization or cluster inference (Supplementary Data)."*

4. Chromatin enrichment analyses appear thyroid-specific, whereas expression-based enrichment is not; yet colocalization signals are strongest in thyroid tissue. Please reconcile these findings and provide gene-level examples to clarify this apparent discrepancy.

Response:
We thank the reviewer for highlighting these apparent differences. This apparent discrepancy reflects differences in the underlying data types and analytical frameworks rather than a biological inconsistency.

First, our functional enrichment analyses are annotation-based and use annotations generated via LD Score regression with Specifically Expressed Genes (LDSC-SEG) (Finucane et al., *Nat Gen* 2015). These analyses test whether cluster-specific thyroid cancer index variants are enriched within thyroid-relevant regulatory annotations derived from independent epigenomic datasets. They do not assess whether individual variants quantitatively alter chromatin accessibility; instead, they evaluate whether associated variants preferentially overlap regulatory regions active in specific tissues. Thus, chromatin enrichment reflects tissue-specific regulatory context rather than variant-level functional effects measured in the same individuals.

Differences between chromatin-based and expression-based enrichment results are therefore expected and may also reflect differences in statistical power across annotation datasets, particularly when regulatory effects are modest or heterogeneous.

Additionally, the scope of variants differs across analyses: functional enrichment was performed on cluster-specific subsets of thyroid cancer index variants (N=2-12 per cluster), whereas colocalization analyses were conducted across all 66 index variants. This difference in variant set size likely contributes to the observation of strong thyroid colocalization signals without significant expression-based enrichment in the smaller Thyroid Gland Dysfunction cluster, which includes only 12 variants.

5. Several genes were tested using very few cis variants, which may reduce the reliability of posterior probabilities and introduce bias. Please re-run analyses using standard cis windows and ancestry-matched LD references. Additionally, consider methods that relax the single-causal-variant assumption (e.g., coloc.susie, pwcoco), and include regional association and colocalization plots to support key findings.

Response:
We thank the reviewer for this suggestion, which helped us identify an error in our original analysis. In the original analysis, colocalization was performed using GTEx v10 significant cis-eQTL pairs, which include only variants passing GTEx significance thresholds and can result in a limited number of cis variants for some gene-tissue pairs. To address this concern, we re-ran colocalization analyses using the full set of cis-eQTL summary statistics from GTEx v10, which include all variants tested within the standard GTEx cis window. In our new analyses, we explicitly restricted variants to those located within ±1 Mb of the transcription start site,

consistent with the GTEx definition of cis regulation and standard practice in colocalization studies. This substantially increased the number of variants contributing to each test and improved the stability of posterior probability estimates. We have updated the colocalization results (**Figure 4**; previous Figure 3) in the manuscript and **Supplementary Figures 8-10** (previous Supplementary Figures 5-7) and **Supplementary Tables 13-15**.

Regarding LD, the coloc framework we applied corresponds to the original Bayesian colocalization method (Giambartolomei et al. *PLoS Genet* 2014), which operates directly on summary statistics and does not require LD information as input. It assumes at most one causal variant per trait within the tested region and evaluates the posterior probability that the same variant underlies association signals in both traits. In contrast, newer fine-mapping approaches such as SuSiE—and extensions such as coloc.susie that integrate multi-variant fine-mapping into colocalization—explicitly model multiple causal variants and require an LD matrix from an ancestry-matched reference panel. Our primary analyses therefore relied on the summary-statistic-based coloc implementation, which does not depend on external LD reference panels.

We further emphasize that our objective is comparative colocalization across tissues using a uniform framework, rather than locus-specific causal inference. Accordingly, we applied the same colocalization method and cis-window definition consistently across all tissues to ensure comparability. While methods that relax the single-causal-variant assumption (e.g., coloc.susie) are valuable for fine-mapping, they require ancestry-matched LD reference panels. Given that our thyroid cancer GWAS meta-analysis is multi-ancestry, selecting an appropriate LD reference would be non-trivial and could introduce additional uncertainty. As fine-mapping is outside the scope of the present study, we have therefore focused on standard coloc analyses using full cis summary statistics.

6. Please clarify whether observed TSH effects reflect feedback mechanisms or gland growth pathways. To address potential reverse causation due to treatment-induced TSH changes, consider employing instrumental variable (IV) approaches with TSH-specific genetic instruments.

Response:
We agree that observational associations between TSH and thyroid cancer may reflect feedback mechanisms related to tumor growth or treatment-induced changes in TSH rather than a direct etiologic effect. To address this concern and reduce the potential for reverse causation, we performed a two-sample Mendelian randomization analysis using genetic instruments specific to circulating TSH levels derived from the ThyroidOmics consortium (Sterenborg et al., Nat Commun 2024), which are fixed at conception and therefore not influenced by disease status or treatment. Using these instruments, genetically proxied higher TSH levels were associated with a lower risk of thyroid cancer, with a significant inverse effect observed using inverse-variance weighted analysis ($\beta = -0.53$, SE = 0.08, p = 4.79E-11) (**Supplementary Figure 5, Supplementary Table 19**). This association was consistent across pleiotropy-robust estimators, including the weighted median ($\beta = -0.17$, p = 8.95E-03), weighted mode ($\beta = -0.39$, $p = 2.8 \times 10^{-5}$), and MR-Egger regression ($\beta = -0.70$, p = 1.51E-06), supporting an

effect of lifelong TSH variation on thyroid cancer risk and arguing against reverse causation as the primary explanation. The inverse causal association between TSH and thyroid cancer has been reported previously (OR_{IVW}=0.55, 95% CI 0.40-0.74) (Zhou et al. *Nat Comm* 2020). These results have now been incorporated into the Results, Methods, and Discussion.

Results: *"Consistent with this pattern, our Mendelian randomization analyses demonstrated an inverse causal association between genetically proxied TSH levels and thyroid cancer risk (βIVW=−0.53, p=4.79E-11; Supplementary Figure 5, Supplementary Table 19), in line with prior studies(Zhou et al. 2020), providing an independent line of evidence that the negative contribution of TSH within these clusters reflects biologically meaningful variation rather than reverse causation."*

Methods: *"Mendelian randomization analysis of TSH and thyroid cancer*
We conducted two-sample Mendelian randomization (MR) analyses to evaluate potential causal relationships between thyroid function–related traits and thyroid cancer risk. For circulating thyroid-stimulating hormone (TSH), genetic instruments were obtained from the ThyroidOmics consortium(Sterenborg et al. 2024), which provides genome-wide association summary statistics for TSH measured in individuals without diagnosed thyroid disease. Restricting to euthyroid individuals reduces potential bias related to disease status or treatment. Independent genome-wide significant variants were selected as instruments using the conditionally independent lead variants reported in the original GWAS publication. Thyroid cancer outcome summary statistics were derived from the meta-analysis performed in the present study. Primary causal estimates were obtained using the inverse-variance weighted (IVW) method under a multiplicative random-effects model. Sensitivity analyses were performed using MR-Egger regression, weighted median, and weighted mode estimators to assess robustness to violations of instrumental variable assumptions. Horizontal pleiotropy was further evaluated using the MR-Egger intercept test. To identify and correct for potential outlier instruments, we applied the MR-PRESSO(Verbanck et al. 2018) framework and repeated analyses after exclusion of detected outliers. Heterogeneity across instruments was assessed using Cochran's Q statistic. Instrument details and full sensitivity results are provided in Supplementary Figure 5 and Supplementary Table 19."

Discussion: *"Importantly, Mendelian randomization analyses demonstrated a robust inverse association between genetically proxied TSH levels and thyroid cancer risk, consistent with prior studies (Zhou et al. 2020). This finding provides orthogonal support that the negative loading of TSH within these clusters reflects biologically meaningful variation in thyroid hormone regulation rather than reverse causation driven by disease or treatment effects. In this context, the Thyroid Gland Dysregulation cluster may capture genetic perturbations that alter glandular homeostasis and proliferative signaling under conditions of suppressed TSH, potentially predisposing to neoplastic transformation."*

Supplementary Figure 5

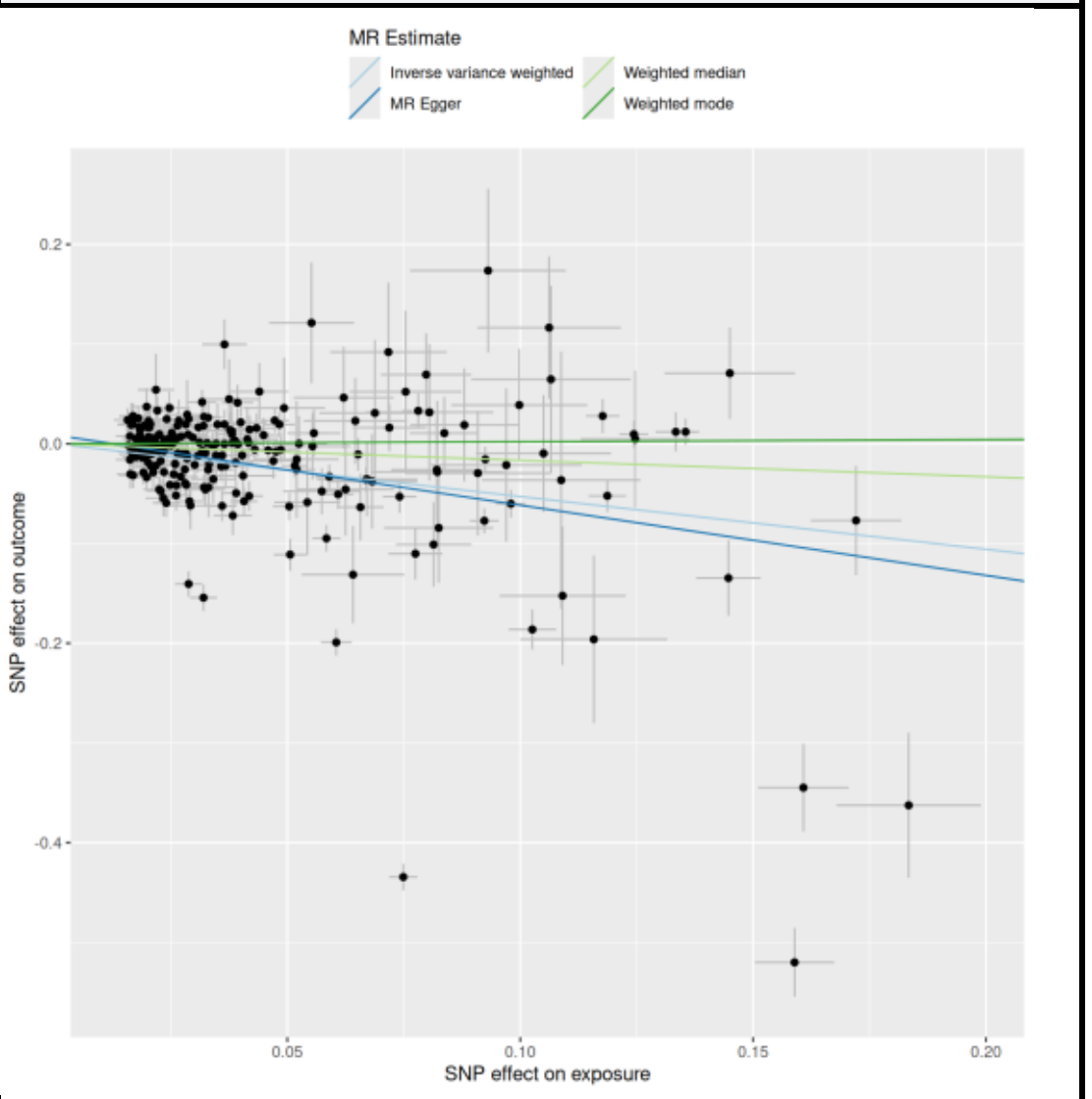

Supplementary Table 19			
**MR method**	**β estimate**	**SE**	**p-value**
Inverse-variance weighted	-0.52901583	0.08042927	4.79E-11
MR-Egger (slope)	-0.70420404	0.14257304	1.51E-06
Weighted median	-0.16536616	0.06325849	8.95E-03
Weighted mode	0.02097389	0.07509989	7.80E-01

7. Avoid overinterpreting the "Mixed function" cluster. Incorporate single-cell or tissue-specific annotations and perform leave-one-locus-out analyses to determine whether associations are driven by a small number of influential signals.

Response:

We agree and have revised the Discussion to more explicitly limit inference regarding the Mixed Function cluster and to clarify that the significant pathway enrichment result (nuclear mineralocorticoid receptor binding) was largely driven by a single implicated gene. We therefore reduced mechanistic and translational speculation and reframed this cluster as a hypothesis-generating signal requiring additional validation.

Regarding leave-one-locus-out analyses, our clustering framework assigns loci to clusters probabilistically (i.e., each locus can contribute to multiple clusters rather than being uniquely assigned). As a result, a direct leave-one-locus-out analysis of “cluster membership” is not well-defined in the same way as it would be for hard cluster assignments, because removing one locus does not correspond to removing a single cluster-specific component. We now explicitly note this limitation and instead outline locus-level sensitivity checks as a future direction

alongside incorporating thyroid-relevant tissue and single-cell functional annotations to assess whether the cluster signal is supported in relevant cellular contexts.

8. Given the limited number of medullary thyroid cancer (MTC) cases, I recommend treating MTC-related findings as exploratory.

Response:
We agree with the reviewer and have revised the Results text to explicitly describe MTC-related findings as exploratory. We now emphasize the limited statistical power due to the small number of MTC cases and frame any nominal associations as hypothesis-generating rather than definitive.

> *"In contrast, effect sizes in MTC were generally attenuated or absent, and should be interpreted as exploratory, given both its distinct cellular origin from parafollicular C cells and the limited number of cases (n=75), which constrains statistical power."*

9. Please make the primary analysis code publicly available, including scripts for meta-analysis result processing and the bNMF implementation (with details on random seed and initialization).

Response:
We have made the primary analysis code publicly available at: https://github.com/yonhojee/thyroid_cancer_bnmf.

The repository includes fully documented scripts covering the complete analysis pipeline, including:

1. Meta-analysis processing
2. Summary statistics retrieval and harmonization
3. Construction of the variant–trait Z-score matrix
4. Implementation of bNMF clustering
5. Downstream enrichment analyses
6. Mendelian randomization analyses

For the bNMF implementation, we provide details on random seed specification and initialization procedures to ensure reproducibility. In addition, we include convergence diagnostics for each run, with scripts generating convergence plots based on the objective function (n.error) and automatic relevance determination parameters (n.lambda). These diagnostics confirm stabilization of the model well before the 10,000-iteration limit across random initializations.

Minor Revisions:
1. Clarify whether LD pruning was applied during pPGS construction. In addition to weighted summation, indicate whether sparse (e.g., lasso) or Bayesian shrinkage models were evaluated to reduce overfitting.

Response:

No LD pruning or shrinkage methods were applied during pPGS construction. Cluster-specific pPGS were computed as weighted sums of variants selected based on bNMF clustering weights.

We did not apply sparse regression (e.g., lasso) or Bayesian shrinkage models because the goal of the pPGS was not prediction optimization but biological interpretation of cluster-specific genetic profiles. We have clarified this explicitly in the Methods section to avoid ambiguity.

2. There is a percentage typo in the subtype overlap description. In the Results section, “71% (165/2,334)” should be corrected to “7.1% (165/2,334).”

Response:
The value has been corrected to “7.1% (165/2,334)” in the Results section.

3. Include sensitivity analyses restricted to “pure PTC” and “pure FTC” cases, given the observed co-diagnoses in certain datasets.

Response:
We thank the reviewer for this helpful suggestion. We conducted sensitivity analyses restricting to “pure PTC” (N=2,284) and “pure FTC” cases (N=212) (i.e., excluding individuals with co-diagnoses across histological subtypes) in FinnGen R12. Reassuringly, effect estimates from these restricted analyses were highly consistent with those from the primary, inclusive analyses reported in the manuscript, with the same direction of effect and comparable magnitudes across loci.

In particular, loci that showed strong and consistent associations in the primary analysis remained significant in both restricted PTC and FTC analyses. For example, *CHEK2* (rs186430430) showed positive associations with both subtypes (pure PTC $\beta = 1.31$, $p = 1.15 \times 10^{-28}$; pure FTC $\beta = 1.02$, $p = 0.016$), as did *EIF4ENIF1* (rs34152346; pure PTC $\beta = 0.77$, $p = 7.41 \times 10^{-12}$; pure FTC $\beta = 0.88$, $p = 0.012$). Similarly, the well-established *PTCSC2/FOXE1* locus (rs4273946) showed consistent protective effects in both subtypes (pure PTC $\beta = -0.46$, $p = 8.00 \times 10^{-54}$; pure FTC $\beta = -0.28$, $p = 0.0029$). These estimates closely mirror those reported in the main analysis using inclusive case definitions.

Together, these results indicate that the observed subtype-specific and shared genetic associations are not driven by co-diagnosed cases and are robust to restriction to histologically pure PTC and FTC. We have added these results to the **Supplementary Table 3** and **Supplementary Figure 2** and now explicitly reference them in the main text.

> Results: *“Sensitivity analyses restricted to histologically exclusive PTC and FTC cases yielded effect estimates highly consistent with the primary analyses, with the same direction and similar magnitudes across loci, indicating that the observed subtype-specific patterns were not driven by co-diagnosed cases (Supplementary Figure 2b, Supplementary Table 3).”*

Methods: *“To assess heterogeneity of germline risk across thyroid cancer subtypes, we used subtype-specific thyroid cancer GWAS summary statistics from FinnGen R12110 to compare effect sizes for 66 lead variants identified in the multi-ancestry meta-analysis. Subtype-specific summary statistics were available for papillary thyroid carcinoma (PTC), follicular thyroid carcinoma (FTC), and medullary thyroid carcinoma (MTC); summary statistics for anaplastic thyroid carcinoma (ATC) were not available, likely due to the small number of cases. Effect sizes (log-odds ratios) were compared across subtypes to evaluate concordance and heterogeneity of genetic associations. We quantified overlap in subtype diagnoses to characterize co-diagnosis across histologies (Supplementary Figure 2a). Given the observed co-diagnoses, we conducted sensitivity analyses restricting to histologically exclusive PTC (N=2,284) and FTC cases (N=212), defined as individuals with only a single histological subtype diagnosis (Supplementary Figure 2b).”*

Supplementary Figure 2b

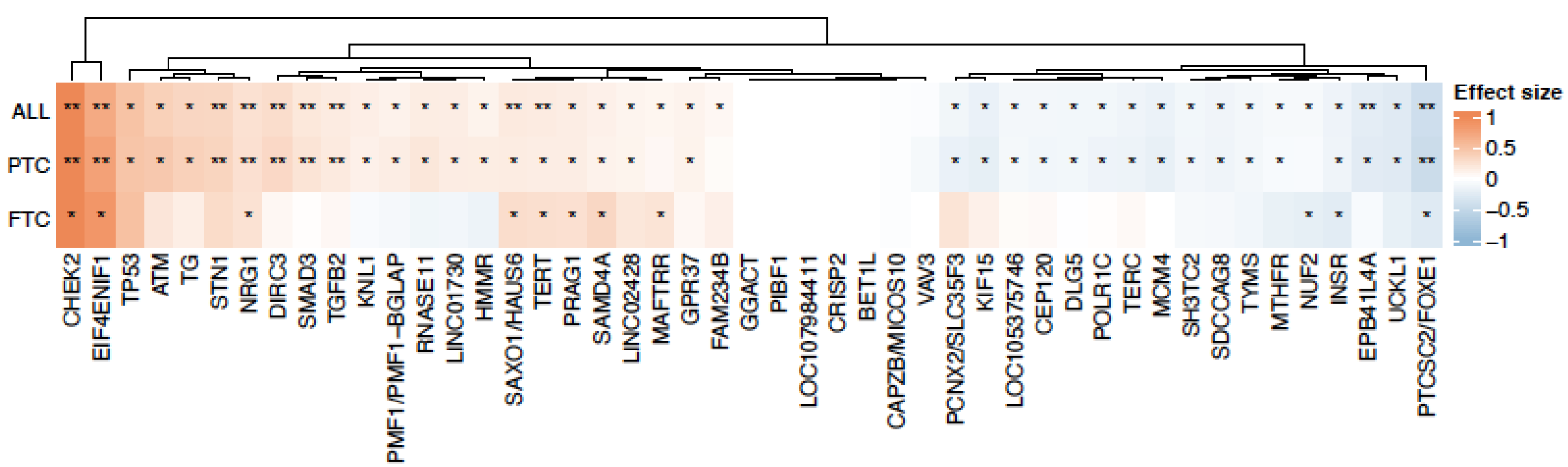

4. Provide ancestry-specific Manhattan and volcano plots, along with a heatmap showing effect-direction concordance for sentinel variants.

Response:

As discussed above, fully ancestry-specific association analyses (and corresponding Manhattan or volcano plots) were not feasible in this study because the results are derived from a meta-analysis across multiple biobanks with differing ancestry compositions and without access to uniformly stratified summary statistics.

To address the reviewer’s underlying concern regarding cross-ancestry consistency, we instead quantified heterogeneity across contributing studies by reporting Cochran’s Q statistics and I^2 values for the lead variant at each locus. In addition, we have included a “Direction” column in the revised **Supplementary Table 2**, derived from the meta-analysis, which reports the sign of effect estimates across contributing studies and provides a direct summary of effect-direction concordance.

5. Correct the title of Table S8 to: “Table S8. The number of cases used for individual-level association tests in All of Us data across ancestry groups.”

Response:
The title of Table S8 has been corrected.

6. Some fraction cells in supplementary tables are incorrectly displayed as dates due to automatic formatting (e.g., "2/8" appears as "8-Feb"). Please correct these formatting issues.

Response:
The formatting has been corrected.

REVIEWERS' COMMENTS

Reviewer #1 (Remarks to the Author):

The authors have extensively taken all of my remarks into account, including revised interpretations of results and extra analyses, which his has now led to a well-balanced manuscript. I am happy with the current version, and have no additional suggestions.

Reviewer #2 (Remarks to the Author):

I appreciate the authors answers to my concern and do not have any further comments on the manuscript.

Reviewer #3 (Remarks to the Author):

I thank the authors for their careful revisions in response to my previous comments. Overall, I believe that my main scientific concerns have now been largely addressed. However, before the manuscript is accepted, I recommend a small number of necessary minor revisions to ensure consistency across figure legends, and terminology, and to further moderate a few statements.

1.Please revise the wording in Figure 4 and its legend. The Methods section now correctly states that the colocalization analysis used all tested variants within the standard GTEx v10 cis-window, but the figure legend still refers to “significant eQTLs,” which may create confusion.

Response: We thank the reviewer for noting this inconsistency. We have revised the Figure 4 legend to remove the phrase “significant eQTLs” and to clarify that the colocalization analyses used all variants tested within the standard GTEx v10 cis-window for each gene-tissue pair, restricted to variants within ±1 Mb of the transcription start site. The revised legend is now consistent with the Methods section.

2.Please standardize the cluster terminology throughout the manuscript. The current text appears to use both “Thyroid Gland Dysfunction” and “Thyroid Gland Dysregulation,” and a single term should be used consistently.

Response: We thank the reviewer for this helpful comment. We have standardized the cluster terminology throughout the manuscript and now consistently use the term “Thyroid Gland Dysfunction” in place of “Thyroid Gland Dysregulation.”

3.I suggest slightly softening the discussion regarding clinical stratification, personalized treatment, and precision oncology. The current data support genetic heterogeneity and biological subclassification well, but direct clinical applicability remains to be established.

Response: We thank the reviewer for this thoughtful suggestion. We have revised the Discussion to soften statements regarding clinical stratification, personalized treatment, and precision oncology. Specifically, we now emphasize that our findings support genetic heterogeneity and biologically informed subclassification, while clarifying that direct clinical applicability for risk stratification, treatment selection, or precision oncology remains to be established.

> ***"These findings suggest that genetic clustering may <u>provide a framework for characterizing biological heterogeneity among individuals with genetic predisposition to thyroid cancer</u>. … <u>Although our findings support the etiologic relevance of genetically informed subclassification, direct clinical utility for risk stratification, treatment selection, or precision oncology remains to be established</u>. Future studies <u>integrating germline genetic clustering with tumor molecular profiles, treatment exposures, and longitudinal clinical outcomes will be needed to determine</u> whether patients stratified by genetic clusters exhibit differential prognosis or responses to targeted therapies."***
